# A mitochondria-driven quality control mechanism for peroxisomal membrane proteins

**DOI:** 10.1038/s41467-026-74117-6

**Published:** 2026-06-10

**Authors:** Sarin Segev-Nakar, Itay Koren

**Affiliations:** The Mina and Everard Goodman Faculty of Life Sciences, Bar-Ilan University, Ramat-Gan, Israel

**Keywords:** Peroxisomes, Ubiquitin ligases

## Abstract

Peroxisomes are essential organelles involved in lipid and reactive oxygen species metabolism, and their function requires proper targeting of peroxisomal membrane proteins (PMPs). When peroxisome biogenesis fails, as occurs in peroxisome biogenesis disorders, PMP levels decrease markedly, yet the underlying mechanisms remain unclear. Here, using quantitative proteomics and transcriptomics in peroxisome-deficient cells, we observe widespread post-transcriptional downregulation of PMPs driven by increased protein turnover via ubiquitination and proteasomal degradation. An unbiased CRISPR screen uncovers a mitochondrial quality control axis. PMPs that fail to reach their native peroxisomal destination are rerouted to mitochondria, where the mitochondrial outer membrane E3 ligases MUL1 and MARCH5 act redundantly to promote their degradation. Importantly, the transmembrane domain of PMPs is sufficient to drive their mitochondrial turnover. Functionally, simultaneous loss of peroxisomes and mitochondrial E3 ligases severely impairs cell proliferation, underscoring the essential role of this pathway. Together, these findings provide insight into the pathology of organelle dysfunction and reveal an inter-organelle quality control axis in which mitochondria act as a surveillance hub to clear PMPs and maintain cellular proteostasis when peroxisomes are absent.

## Abstract (web summary)

Mitochondria and peroxisomes share functional links to maintain cell viability. Here, the authors reveal a mitochondria-driven quality control mechanism where mitochondrial E3 ligases clear mistargeted peroxisomal membrane proteins during peroxisome biogenesis failure, preventing cellular toxicity.

## Introduction

Proteins serve as molecular machines that drive most cellular processes, and thus maintenance of protein homeostasis (proteostasis) is crucial for cellular and organismal health^1^. Accordingly, when proteostasis is disrupted, it can contribute to the development and progression of a variety of pathological disorders, including inflammation, neurodegeneration and cancer^2^. To preserve proteostasis, cells have evolved elaborate protein quality control (PQC) machinery that monitors and maintains a healthy proteome by refolding, sequestering, or degrading aberrant proteins. Key components of PQC include chaperones and proteolytic pathways, with the ubiquitin-proteasome system (UPS) playing a central role in the degradation of a significant portion of intracellular proteins^3^.

The UPS employs a three-step cascade to mediate selective substrate ubiquitination, which in turn promotes substrate degradation. The E1 ubiquitin-activating enzymes activate ubiquitin molecules, which are then transferred to E2 ubiquitin-conjugating enzymes (E2s) and eventually to substrates via E3 ligases^4^. E3 ligases are of crucial importance for establishing the specificity of protein degradation via the UPS, as they select the substrates to be ubiquitinated. In most cases, E3 ligases can directly associate with substrates; however, in some cases, chaperones or other cellular factors bridge between the substrates and an E3 ligase^5^.

PQC mechanisms deal with different types of aberrant proteins within cells, such as misfolded, orphaned and mislocalized proteins, to limit their proteotoxic effect^1,6–9^. Mislocalized proteins, those that fail to reach their correct cellular destination, are managed by several dedicated quality control mechanisms in mammals. One of the primary systems involves the chaperone BAG6, which primarily targets mislocalized membrane proteins, especially those destined for the endoplasmic reticulum (ER). When these proteins are mislocalized to the cytoplasm, their hydrophobic transmembrane domains (TMDs) are exposed, triggering BAG6 to recognize them and recruit the E3 ligase RNF126 to mediate the degradation of the mislocalized proteins via the proteasome^10–12^. A parallel pathway operates for certain classes of mislocalized mitochondrial membrane proteins. Ubiquilin proteins recognize exposed hydrophobic domains in these proteins and promote their degradation, although the specific E3 ligase involved remains unidentified^13,14^. For secretory proteins with a less hydrophobic signal peptide, another PQC mechanism exists. When these proteins fail to translocate across the ER membrane, an N-degron within the signal peptide is exposed by DPP8/9 proteases, enabling UBR-family E3 ligases to target them for degradation^15^. Importantly, the recognition and clearance of mislocalized proteins is not restricted to membrane-associated proteins. For mitochondrial matrix proteins that are mislocalized to the cytoplasm, the E3 ligase UBR4 mediates their degradation based on recognition of their mitochondrial targeting signals^16,17^.

Overall, the study of PQC mechanisms highlights two key aspects. First, mislocalized proteins, especially hydrophobic ones, that are not efficiently cleared can aggregate via their exposed hydrophobic domains, disrupting cytoplasmic protein function and compromising cellular integrity. This underscores the critical role of PQC systems in preventing proteotoxic stress and maintaining cellular homeostasis. Second, although distinct PQC mechanisms involve different components, in all cases the targeting signal plays a dual role, governing both localization and degradation. We hypothesize that similar mechanisms may also act on mislocalized proteins from other organelles, such as peroxisomes, by recognizing their targeting signals.

Peroxisomes are vital metabolic organelles involved in key cellular processes, such as fatty acid β-oxidation, ether lipid (plasmalogen) synthesis, and redox homeostasis^18^. Like other organelles, peroxisomes rely on specialized protein import systems for their biogenesis and maintenance^19,20^. Specifically, PEX3 and PEX19 play pivotal roles in coordinating the targeting and import of peroxisomal membrane proteins (PMPs). PEX19 acts as a cytosolic chaperone and receptor, forming a complex with newly synthesized PMPs and guiding them to the peroxisome. PEX3 functions as a docking site on the peroxisomal membrane for these PEX19-PMP complexes. Upon docking, PEX19 releases its cargo, allowing the PMPs to integrate into the peroxisomal membrane, and is then recycled back to the cytosol. Given their essential roles in PMP biogenesis, it is not surprising that loss of function in *PEX3* or *PEX19* leads to the most severe forms of peroxisome biogenesis disorders (PBDs), including Zellweger spectrum disorders (ZSDs)^21^.

Here, we investigate the PQC mechanisms responsible for recognizing and degrading mislocalized PMPs. To induce PMP mislocalization, we disrupt peroxisome biogenesis by ablating *PEX3* or *PEX19* in human cells. Proteomic and transcriptomic analyses in *PEX3*- or *PEX19*-deficient cells reveal marked post-transcriptional downregulation of PMP levels. To explore the involvement of the UPS in this process, we generate green fluorescent protein (GFP)-tagged reporters and analyze their stability in cells lacking *PEX3* or *PEX19*, demonstrating ubiquitin- and proteasome-dependent degradation of mislocalized PMPs. To identify the E3 ligases responsible, we perform a CRISPR-based screen targeting all known human E3 ligases and identify the mitochondrial E3 ligases MUL1 and MARCH5 as key regulators of PMP stability. Through microscopy and subcellular fractionation in cells lacking peroxisomes, we demonstrate that loss of *MUL1* and *MARCH5* leads to mitochondrial accumulation of mislocalized PMPs, whereas proteasome inhibition results in their cytosolic accumulation, supporting a model in which mitochondrial E3 ligases promote ubiquitination of PMPs at mitochondria prior to their extraction from the membrane and subsequent proteasomal degradation in the cytosol. Moreover, the TMD of PMPs is sufficient to confer susceptibility to this mitochondrial quality control pathway.

Collectively, these findings reveal a PQC pathway in which disruption of PMP targeting results in their rerouting to mitochondria and elimination via mitochondrial E3 ligase–mediated degradation. This mechanism highlights inter-organelle cooperation and a role for mitochondria in maintaining proteostasis when peroxisome biogenesis is impaired, with potential disease relevance, as the accumulation of mitochondria-localized PMPs may further exacerbate mitochondrial dysfunction in PBDs.

## Results

### Peroxisome loss leads to broad downregulation of peroxisomal proteins

To investigate the adaptive response of cells to defects in PMP biogenesis, we generated HEK293T cells lacking the key biogenesis factors *PEX3* or *PEX19* using CRISPR/Cas9-mediated gene disruption. Cells edited with an sgRNA targeting the *AAVS1* safe-harbor locus were used as a control knockout (KO), an established negative control for CRISPR-based experiments^22^. Successful KO of these proteins was confirmed by genome sequencing (Supplementary Fig. 1a) and immunoblotting (Supplementary Fig. 1b, top panel). As a functional readout, we expressed GFP fused to the peroxisomal matrix protein catalase (CAT), which contains a peroxisomal targeting signal type 1 (PTS1), a well-established reporter for monitoring peroxisome integrity and localization in cells^23^. In wild-type HEK293T cells, GFP-CAT exhibited a punctate fluorescence pattern, consistent with its accumulation in functional peroxisomes. In contrast, *PEX3* or *PEX19* KO cells showed a complete loss of GFP-CAT puncta; instead, GFP-CAT displayed diffuse cytoplasmic fluorescence, indicating a substantial loss of peroxisomes (Supplementary Fig. 1c). Consistently, a recently developed peroxisome-specific probe PeroxiSPY650^24^ confirmed the complete absence of peroxisomes in these KO cells (Supplementary Fig. 1d, e).

**Fig. 1:**
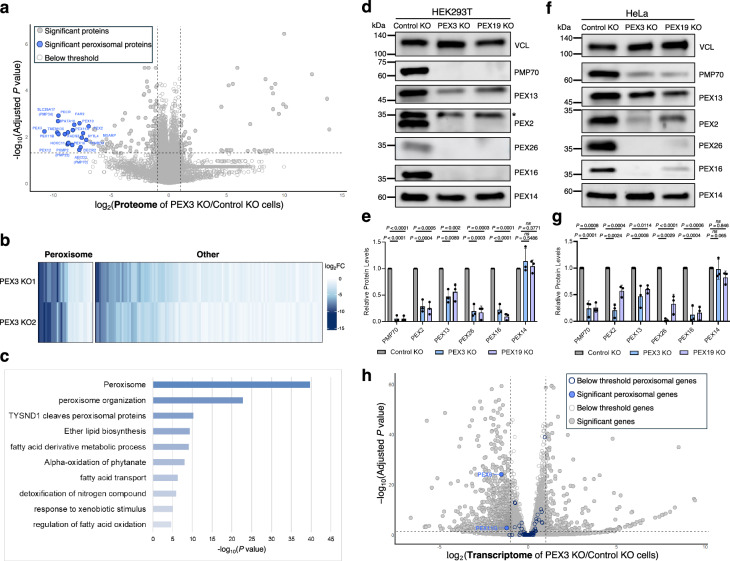
Loss of peroxisome biogenesis factors induces selective post-transcriptional downregulation of peroxisomal membrane proteins (PMPs). **a** Volcano plot displaying the log_2_ fold change (FC) versus -log_10_(*P* value) from MS proteomics analysis of the total proteome, comparing control KO cells to two independent *PEX3* KO cells. Significance thresholds (dashed lines) were set at a log₂FC ≥ 1 or ≤ −1 (representing a ≥ 2-fold change in either direction) and an adjusted *P* < 0.05, as determined by a two-sided Welch’s *t*-test. Gray filled circles indicate proteins with significant changes in abundance between the control and *PEX3* KO proteomes. Empty gray circles represent proteins that did not meet the FC or statistical significance thresholds. Significantly downregulated peroxisomal proteins are highlighted in blue and labeled with their gene names. **b** Heatmap showing the log₂FC differences between control KO cells and two independent *PEX3* KO cell lines for significantly downregulated proteins, highlighting the pronounced reduction of peroxisomal proteins compared to non-peroxisomal (‘Other’) proteins upon *PEX3* loss. **c** Functional enrichment analysis of the significantly downregulated proteins, confirming a strong enrichment for peroxisomal components and pathways. Statistical significance of the enrichment was determined by Metascape using a one-sided hypergeometric test, with *P* values adjusted for multiple comparisons using the Benjamini-Hochberg procedure to control the false discovery rate (FDR). **d–g** Immunoblot analysis of endogenous PMPs (PMP70, PEX13, PEX2, PEX26, PEX16, PEX14) in HEK293T (**d**, **e**) and HeLa (**f**, **g**) cells lacking *PEX3* or *PEX19*. Vinculin (VCL) serves as a loading control. * denotes non-specific band observed in PEX2 blots. Representative immunoblots (**d**, **f**) and quantitative analyses (mean ±  s.d.) from *n* = 3 biological replicates are shown (**e**, **g**). *P* values determined by two-tailed unpaired *t*-test and are indicated in the figure. **h** Volcano plot showing the log₂FC versus -log_10_(*P* value) from RNA-seq comparing control KO cells to two independent *PEX3* KO lines. Differential expression was analyzed independently for each *PEX3* KO line using two-sided Wald test. *P* values were combined by Fisher’s method and adjusted for multiple testing using the Benjamini–Hochberg FDR procedure. Significance thresholds (dashed lines) were set at a log₂FC ≥ 1 or ≤ −1 and an adjusted *P* < 0.05. Filled gray circles represent genes with significant expression changes, while empty gray circles indicate genes not meeting FC or statistical thresholds. Peroxisomal genes are highlighted in blue: empty blue circles denote unchanged peroxisomal genes, and filled blue circles indicate significantly downregulated peroxisomal genes, labeled with their gene names. Source data are provided as a Source Data file.

Next, we performed label-free mass spectrometry (MS)-based proteomic analysis on *PEX3* KO HEK293T cell lines. To minimize the potential influence of CRISPR/Cas9 off-target effects, we used two independently derived clones for this analysis (Supplementary Fig. 1b, bottom panel). Principal component analysis (PCA) revealed distinct proteomic profiles between the *PEX3* KO and control KO cells (Supplementary Fig. 2a). Of 9599 quantified proteins, 149 were significantly downregulated and 63 were upregulated in *PEX3*-deficient cells compared to control cells (Welch’s *t*-test, *p* < 0.05; log_2_ fold change < −1 for downregulated and > +1 for upregulated proteins) (Fig. 1a, b and Supplementary Data 1).

As expected, PEX3 was among the most strongly reduced proteins (Fig. 1a and Supplementary Fig. 2b). Notably, functional enrichment analysis^25^ revealed that the most significantly decreased proteins were enriched for peroxisomal components and pathways (Fig. 1c), accounting for approximately 25% of all downregulated proteins (Supplementary Data 1). While the majority of these are PMPs, several matrix proteins, including PECR and DECR2, were also significantly reduced (Supplementary Fig. 2b). Importantly, of the 69 established peroxisomal proteins^26^ confidently detected in our MS dataset, over 50% were significantly decreased. Notably, nearly all PMPs were affected, with downregulation observed for every detected PMP except PEX14 (Supplementary Fig. 2b,c; Supplementary Data 1).

Among the downregulated non-peroxisomal proteins, ~35% of this group were metabolic enzymes and ~18% are mitochondrial proteins, consistent with significant pathway enrichments in carboxylic acid metabolism, biological oxidations, and sulfur compound metabolic processes (Supplementary Fig. 2d and Supplementary Data 1).

To validate the proteomics findings, we performed western blot analysis using specific antibodies against endogenous representative PMPs in the parental CRISPR-edited *PEX3* and *PEX19* KO HEK293T cell populations (Supplementary Fig. 1b, top panel). In line with the MS data, the PMPs PMP70 (encoded by *ABCD3*), PEX26, PEX16, and PEX2 were undetected in both *PEX3* and *PEX19* KO HEK293T cells, while PEX13 protein levels were markedly reduced (Fig. 1d, e). In contrast, PEX14 levels remained unchanged (Fig. 1d, e), consistent with previous reports^27,28^. Importantly, this phenotype is not cell-line specific, as similar patterns were observed in HeLa *PEX3* and *PEX19* KO cells (Fig. 1f, g and Supplementary Fig. 1b). Notably, the upper band observed in the PEX2 immunoblot represents a nonspecific signal, as confirmed in CRISPR-generated *PEX2* KO cells (Supplementary Fig. 1f).

To assess whether the observed protein-level changes were transcriptionally driven, we performed RNA sequencing (RNA-seq) on the same cell backgrounds (Supplementary Data 2). Except for *PEX6* and the PMP *PEX11G*, all peroxisomal genes showed unchanged mRNA levels (Fig. 1h). Similarly, 82% of non-peroxisomal genes that were downregulated at the protein level, also showed no change at the transcript level (Supplementary Data 2).

Importantly, only a small fraction of the proteome (~1%) was downregulated in *PEX3* KO cells, and levels of established short-lived proteins (half-life <8 h), such as REST, JUN, and PRELID3B^29^ remained unaffected (Supplementary Data 1). Notably, only four of the downregulated proteins (LONP2, SAMD11, EGFL7, and GPC3) (Supplementary Data 1) are known to be short-lived^29^, suggesting that most of the downregulated proteins are normally long-lived, and that the absence of peroxisomes specifically triggers a reduction in their stability. Collectively, these results suggest that neither a general stress response nor a global inhibition of protein synthesis is induced. Instead, the data support a model in which peroxisome biogenesis failure triggers selective, post-transcriptional regulation of peroxisomal proteins, likely via UPS-mediated degradation.

Based on these findings, we next focused on PMPs to investigate whether they are specifically degraded in response to peroxisome biogenesis failure.

### Fluorescent reporter-based analysis of PMP turnover in peroxisome-deficient cells

**Fig. 2:**
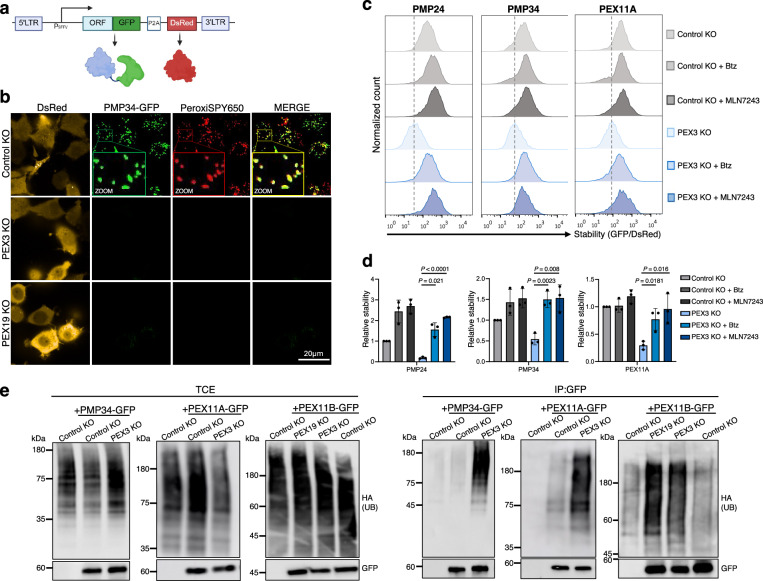
PMPs undergo ubiquitin-proteasome dependent degradation during peroxisomes deficiency. **a** Schematic illustration of the Global Protein Stability (GPS) bicistronic reporter system. The lentiviral construct encodes a PMP-GFP fusion protein and a stable DsRed reference protein separated by a P2A sequence. The GFP/DsRed ratio reflects PMP stability. Created in BioRender. Koren, I. (2026) https://BioRender.com/348xan0. **b** Representative fluorescence microscopy images of PMP34-GFP (a representative PMP reporter) and PeroxiSPY650 (peroxisome-specific reagent) in *PEX3* or *PEX19* KO cells compared to control cells. DsRed serves as an expression control and remains expressed in all cells. MERGE panels show the overlay of PMP34-GFP and PeroxiSPY650 signals. The zoomed regions (ZOOM) demonstrate strong colocalization between the PMP34-GFP and PeroxiSPY650 signals. Scale bar, 20 µm. **c**, **d** Stability analysis of PMP24, PMP34, and PEX11A GPS reporters in control KO or *PEX3* KO cells following 10 h treatment with 1 µM of the proteasome inhibitor bortezomib (Btz) or the E1 inhibitor MLN7243. **c** Representative flow cytometry histograms. The dashed line marks the peak of untreated *PEX3* KO cells, highlighting decreased reporter stability upon *PEX3* loss and the rightward stabilization shift induced by inhibitor treatment, consistent with ubiquitin-proteasome-dependent degradation. **d** Quantification of mean GFP/DsRed fluorescence, normalized to control KO cells. Data represent mean ± s.d. from *n* = 3 biological replicates. *P* values were determined using a two-tailed unpaired *t*-test. **e** In vivo ubiquitination assay. HA-ubiquitin was transfected to control, *PEX3* and *PEX19* KO cells stably expressing PMP-GFP reporters (e.g., GFP-fused PMP34, PEX11A and PEX11B). Cells were then treated with the proteasome inhibitor bortezomib (1 µM, 5 h) followed by IP with anti-GFP beads. Control KO cells not expressing PMP-GFP served as a negative control. Immunoblot analysis was performed on both total cell extracts (TCE) and GFP immunoprecipitates (IP:GFP) using anti-HA antibody to detect HA-ubiquitin (UB) conjugates. Anti-GFP immunoblotting confirmed comparable levels of PMP-GFP in the immunoprecipitates across samples. Source data are provided as a Source Data file.

To characterize the mechanism(s) of PMP degradation in peroxisome-deficient cells, we employed the Global Protein Stability (GPS) system^30^ to generate reporters expressing various PMPs fused to GFP. GPS is a method for monitoring protein stability using a bicistronic lentiviral vector that encodes two fluorescent proteins: GFP fused to a protein of interest (here, an N-terminally tagged PMP), and DsRed as a reference, separated by a P2A peptide (Fig. 2a). Because both proteins are translated from the same mRNA, the GFP/DsRed fluorescence ratio measured by flow cytometry provides a quantitative readout of the relative stability of the GFP fusion protein. A schematic of the flow cytometry gating strategy used in GPS assays is shown in Supplementary Fig. 3a.

We hypothesized that, in the absence of peroxisomes, a PQC mechanism exists to degrade mislocalized PMPs, leading to reduced stability of these proteins in *PEX3-* or *PEX19*-deficient cells. To test this, we expressed a GPS reporter for PMP34 (encoded by *SLC25A17*) as a representative PMP and analyzed its localization by fluorescence microscopy. In control cells, PMP34-GFP showed, as expected, strong co-localization with the peroxisomal probe PeroxiSPY650 (Fig. 2b). In contrast, peroxisomes were absent in *PEX3* or *PEX19* KO cells, as indicated by the loss of the PeroxiSPY650 signal (Fig. 2b and Supplementary Fig. 1d, e). Correspondingly, the GFP signal was undetectable in the KO cells, despite normal DsRed expression (Fig. 2b), consistent with possible degradation of PMP34-GFP under these conditions.

To assess UPS-dependent degradation, we quantified PMP stability using the GPS reporter system. Flow cytometry analysis of cells expressing GPS reporters for the PMPs PMP24 (encoded by *PXMP4*), PMP34 and PEX11A revealed a marked decrease in their stability in *PEX3*-deficient cells (Fig. 2c, d). This reduction in stability could be reversed by treating the cells with the E1 inhibitor MLN7243 or the proteasome inhibitor bortezomib (Btz) (Fig. 2c, d), indicating that UPS-dependent degradation occurs upon peroxisome biogenesis failure. The reduced stability was specific to *PEX3* and not an off-target effect of CRISPR/Cas9, as a similar reduction in PMP stability was observed in independent KO cell clones generated using a second sgRNA (Supplementary Fig. 3b, c). In addition, the same trend was observed in *PEX19*-deficient cells (Supplementary Fig. 3b, c). Moreover, reintroduction of *PEX3* or *PEX19* cDNA restored PMP stability in the respective KO cells (Supplementary Fig. 3b, c), confirming that the observed reduction in PMP stability is a specific, on-target consequence of *PEX3* or *PEX19* loss.

Finally, we provided evidence that peroxisome biogenesis failure induces ubiquitination of PMPs. To test this, GFP-fused PMPs were immunoprecipitated (IP) from cells co-expressing HA-tagged ubiquitin. Prior to IP, cells were treated with the proteasome inhibitor bortezomib to prevent degradation of the substrate and allow accumulation of ubiquitinated proteins. Western blot analysis of the GFP IP using an anti-HA antibody revealed increased ubiquitination of PMPs in *PEX3* or *PEX19* KO cells (Fig. 2e), indicating that, upon peroxisome biogenesis failure, PMPs are ubiquitinated and targeted for proteasomal degradation.

Next, we investigated the PQC pathway responsible for degrading mislocalized PMPs. We first ruled out the involvement of the major PQC factor BAG6, which is known to recognize long stretches of hydrophobic residues and primarily targets mislocalized ER membrane proteins^31^. To test this, we generated CRISPR-mediated KO of both *BAG6* and *PEX3* (*PEX3/BAG6* DKO; Supplementary Fig. 3d), which did not restore the stability of PMPs (Supplementary Fig. 3e), thus excluding BAG6 from contributing to this degradation pathway. Moreover, comparison of the TMD hydrophobicity between PMPs and ER membrane proteins revealed that PMP TMDs are significantly less hydrophobic (Supplementary Fig. 3f), further supporting the notion that mislocalized PMP degradation proceeds via a BAG6-independent mechanism.

### CRISPR screen identifies mitochondrial E3 ligases regulating PMP stability

**Fig. 3:**
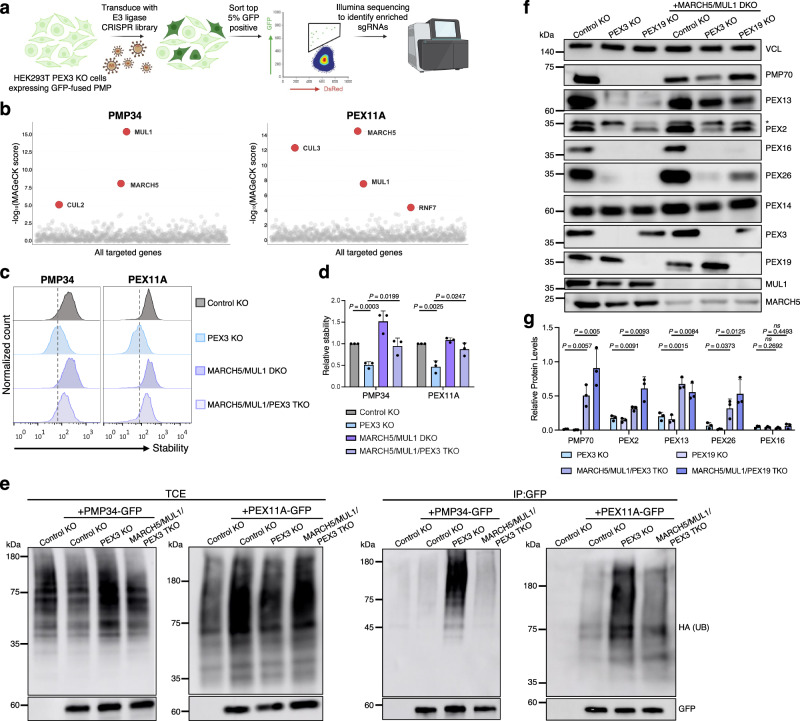
Mitochondrial E3 ligases MUL1 and MARCH5 are redundant regulators of PMP degradation. **a** Schematic of the E3 ligase CRISPR screen pipeline using the PMP-GPS reporter in *PEX3* KO cells. Cells with restored GFP expression following E3 ligase loss are sorted and sgRNA enrichment was determined by next-generation sequencing. Created in BioRender. Koren, I. (2026) https://BioRender.com/060l2z9. **b** MAGeCK analysis of the CRISPR screen results, identifying the mitochondrial E3 ligases *MUL1* and *MARCH5* as the most significantly enriched shared hits for both PMP34 and PEX11A reporters. Significance of enrichment was determined using the robust rank aggregation (RRA) algorithm with Benjamini-Hochberg FDR correction. **c**, **d** Stability analysis of PMP34 and PEX11A GPS reporters in control, *PEX3* KO, *MARCH5/MUL1* double KO (DKO), and *MARCH5/MUL1/PEX3* triple KO (TKO) cells. Representative flow cytometry histograms are displayed in (**c**) and quantification of mean GFP/DsRed fluorescence normalized to control KO ± s.d. from *n* = 3 biological replicates is shown in (**d**). *P* values were determined using a two-tailed unpaired *t*-test and are indicated in the figure. **e** In vivo ubiquitination assay. HA-ubiquitin was transfected to control KO, *PEX3* KO, and *PEX3* KO cells additionally lacking both *MUL1* and *MARCH5* stably expressing PMP34-GFP or PEX11A-GFP, followed by IP with anti-GFP beads. Control KO cells not expressing PMP-GFP served as a negative control. Immunoblot analysis was performed on both total cell extracts (TCE) and GFP immunoprecipitates (IP:GFP) using anti-HA antibody to detect HA-ubiquitin (UB) conjugates. Anti-GFP immunoblotting confirmed comparable levels of PMP-GFP in the immunoprecipitates across samples. **f**, **g** Immunoblot analysis of endogenous PMPs using specific antibodies in *PEX3* or *PEX19* KO cells with or without additional deletion of both *MUL1* and *MARCH5*. Vinculin (VCL) serves as a loading control, and antibodies against PEX3, PEX19, MUL1, and MARCH5 confirm KO efficiency. Representative immunoblot (**f**) and quantitative analyses (mean ±  s.d.) from *n* = 3 biological replicates are shown in (**g**). Quantification was normalized to control KO cells for consistency with analyses throughout the study. *P* values determined by two-tailed unpaired *t*-test and are indicated in the figure. Source data are provided as a Source Data file.

To identify the PQC factors involved in this process in an unbiased manner, we performed a CRISPR screen using an sgRNA library targeting all known E3 ligases^15^. *PEX3* KO cells expressing PMP GPS reporters were transduced with the library, and cells in which GFP expression was restored (due to the ablation of E3 ligases responsible for targeting PMPs for degradation) were sorted. Next-generation sequencing revealed the abundance of sgRNAs in both the sorted and unsorted populations, and enriched sgRNAs were identified using Model-based Analysis of Genome-wide CRISPR-Cas9 KO (MAGeCK) analysis^32^ (Fig. 3a). MAGeCK analysis identified *MARCH5* and *MUL1* as shared top hits in the PMP34 and PEX11A screens (Fig. 3b and Supplementary Data 3). Additionally, *CUL2* and *CUL3* scored in the PMP34 and PEX11A screens, respectively (Fig. 3b and Supplementary Data 3). Using MLN4924, a Cullin-RING ligase inhibitor^33^, we ruled out the involvement of *CUL2* and *CUL3* in promoting PMP degradation (Supplementary Fig. 4a), suggesting these were false positives from the screen. We therefore focused our investigation on MUL1 and MARCH5.

The identification of *MUL1* and *MARCH5* is notable, as these are the main E3 ligases of the mitochondria and are anchored to the outer mitochondrial membrane via their TMDs^34^. Interestingly, MARCH5 has also been suggested to localize to peroxisomes in some cellular contexts^35^. However, since peroxisomes are absent in *PEX3* KO cells (Fig. 2b and Supplementary Fig. 1d, e), these E3 ligases are likely located on the mitochondria, as previously demonstrated in *PEX3* KO cells^36,37^. To validate the results of the screen and investigate potential redundancies between *MUL1* and *MARCH5*, we generated individual KO cells for *MUL1* and *MARCH5*, as well as double KO (DKO) cells, in both *PEX3*-deficient and *PEX3*-proficient backgrounds. While individual KOs of these E3 ligases on the background of *PEX3* deficiency resulted in a minor stabilization effect (Supplementary Fig. 4b), the combined ablation significantly rescued the destabilization of PMP34 and PEX11A (Fig. 3c, d and Supplementary Fig. 4b). Notably, PEX11B CRISPR screens did not reveal any significant E3 ligase hits (Supplementary Data 3) and was not stabilized in cells deficient for *PEX3*, *MUL1* and *MARCH5* (Supplementary Fig. 4b), suggesting that additional or redundant E3 ligases may operate alongside MUL1 and MARCH5 to degrade certain PMPs upon peroxisome biogenesis failure.

To further demonstrate regulation of PMPs by MUL1 and MARCH5, we monitored ubiquitination of GFP-fused PMPs in cells transfected with HA-ubiquitin. IP of PMP-GFP from *PEX3* KO cells, with or without *MARCH5/MUL1* ablation, followed by immunoblotting for HA-ubiquitin, revealed that while PMP34-GFP and PEX11A-GFP are heavily ubiquitinated in *PEX3* KO cells, their ubiquitination is substantially reduced to background levels in *MARCH5/MUL1/PEX3* triple KO (TKO) cells (Fig. 3e). These results demonstrate that MUL1 and MARCH5 regulate PMPs’ turnover by promoting their ubiquitination and degradation upon peroxisome loss.

To extend this finding and determine whether the same mechanism also applies to endogenous PMPs, we performed immunoblot analysis of representative PMPs, comparing their protein levels in *PEX3* or *PEX19* KO cells with or without *MUL1* and *MARCH5* ablation. Interestingly, PEX2, PEX13, PEX26 and PMP70 were reduced in *PEX3* and *PEX19* KO cells, whereas additional KO of *MUL1* and *MARCH5* restored their levels (Fig. 3f, g). In contrast, PEX16 levels were not restored (Fig. 3f, g). PEX14 served as a negative control, as its abundance was not affected by *PEX3* or *PEX19* KOs (Fig. 3f).

Cycloheximide (CHX) chase assays further demonstrated the turnover dynamics of PMPs under these conditions, with PEX13 serving as a representative example. PEX13 exhibited turnover both in control KO cells (where peroxisomes are present) and in *PEX3* KO cells (where peroxisomes are absent) (Supplementary Fig. 4c). Importantly, focusing on peroxisome-deficient cells, comparison of *PEX3* KO and *MARCH5/MUL1/PEX3* TKO cells demonstrated that turnover of PEX13 was completely blocked in the absence of *MUL1* and *MARCH5* (Supplementary Fig. 4c, d), indicating that PEX13 turnover depends on these E3 ligases.

Collectively, these findings establish that the mitochondrial E3 ligases MUL1 and MARCH5 promote the ubiquitination and degradation of both exogenous GPS reporters and endogenous PMPs following peroxisome loss.

### PMPs are redirected to mitochondria for degradation by mitochondrial E3 ligases upon peroxisome loss

Our findings suggest a distinct quality control mechanism: whereas PQC factors, such as BAG6 and Ubiquilins, act on cytoplasmically retained mislocalized ER or mitochondrial membrane proteins, in the absence of peroxisomes, PMPs are redirected to mitochondria and degraded by resident E3 ligases, specifically MUL1 and MARCH5.

**Fig. 4:**
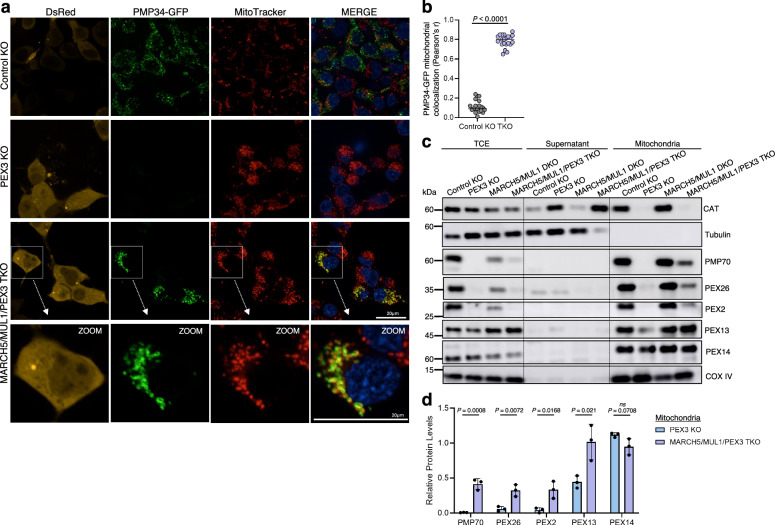
PMPs are rerouted to mitochondria for proteasomal degradation under peroxisome loss. **a** Representative confocal fluorescence microscopy images of PMP34-GFP reporter expressed in the indicated genetic backgrounds of HEK293T cells. Orange, DsRed expression control; green, PMP34-GFP signal; red, mitochondrial marker MitoTracker. MERGE panels show the overlay of the PMP34-GFP, MitoTracker, and Hoechst nuclear stain (blue). The lower panel (ZOOM) shows a magnified view of the boxed region above. Scale bar, 20 µm. **b** Pearson’s correlation coefficient (Pearson’s r) analysis for the colocalization of PMP34-GFP with the mitochondrial marker MitoTracker shows increased colocalization in cells deficient for both *PEX3* and *MUL1/MARCH5* (TKO). Data are presented as individual data points with mean values; *n*  =  21 cells per genetic background. The *P* value was determined by a two-tailed unpaired *t*-test and is indicated in the figure. **c**, **d** Cellular fractionation of the indicated KO cells, followed by immunoblotting of endogenous PMPs in the mitochondria-enriched fraction (Mitochondria) and the mitochondria-depleted supernatant (Supernatant). COX IV and Tubulin serve as mitochondrial or supernatant loading controls, respectively. CAT, a peroxisomal matrix protein, was included to confirm the absence of peroxisomal contamination in the mitochondrial fraction of peroxisome-deficient cells. A representative immunoblot is shown in (**c**), and quantitative analyses (mean ± s.d.) from *n* = 3 biological replicates are shown in (**d**) for the mitochondrial fraction. Quantification was normalized to control KO cells for consistency with analyses throughout the study. *P* values were determined using a two-tailed unpaired *t*-test and are indicated in the figure. ns, not significant. Source data are provided as a Source Data file.

To test this, we examined the localization of GPS PMP reporters in *PEX3* KO cells, with or without mitochondrial E3 ligases, using PMP34 as a representative example. Notably, HEK293T *PEX3*-deficient cells completely lack peroxisomes, as demonstrated by PeroxiSPY650 staining (Supplementary Fig. 5a, b; left panel). As observed previously, fluorescence microscopy confirmed the absence of the GPS reporter in *PEX3* KO cells (Fig. 4a). However, combined deletion of *MARCH5/MUL1* in *PEX3* KO cells not only restored PMP34 protein levels but also allowed PMP34-GFP to accumulate at mitochondria (Fig. 4a). Consistently, Pearson’s correlation (Pearson’s r) analysis between the PMP34-GFP signal and the mitochondrial dye MitoTracker revealed high colocalization in *MARCH5/MUL1/PEX3* TKO cells, but poor correlation in control KO cells (Fig. 4b). This phenomenon was also observed in an additional cell line, HeLa (Supplementary Fig. 5a, b; right panel), using three other PMP-GFP reporters (Supplementary Fig. 6a–c).

To further confirm mitochondrial PMP localization, we performed cellular fractionation followed by immunoblot analysis of mitochondria-enriched fraction (Mitochondria) and a mitochondria-depleted supernatant (Supernatant). Since peroxisomes and mitochondria form extensive contact sites^38^, mitochondria-enriched fractions from control KO and *MARCH5/MUL1* DKO cells contained peroxisomal markers, including PMPs and the matrix protein CAT (Fig. 4c); such contamination has been reported previously^39^, precluding clear discrimination between the two organelles. To overcome this limitation, we focused our analysis on peroxisome-deficient cells (*PEX3* KO and *MARCH5/MUL1/PEX3* TKO cells), in which the absence of peroxisomes (Supplementary Fig. 5a, b) ensures that proteins detected in the mitochondria-enriched fraction originate from mitochondria rather than peroxisomal contamination, as evidenced by the lack of CAT signal in these fractions (Fig. 4c). This approach allowed us to directly assess whether loss of *MUL1* and *MARCH5* specifically increased mitochondrial-associated PMPs. Indeed, endogenous PMP levels were rescued specifically in the mitochondrial-enriched fraction of *MARCH5/MUL1/PEX3* TKO cells, whereas PEX14 levels remained unchanged, as expected (Fig. 4c, d).

Notably, the same trend was observed in a *PEX19* KO background. Cellular fractionation indicated that PMP levels (PMP70, PEX13 and PEX2) were increased in the mitochondrial-enriched fraction of *MARCH5/MUL1/PEX19* TKO cells (Supplementary Fig. 6d). Given that PEX19 functions as a shuttling factor for PMPs to the peroxisome, these findings are in line with previous studies demonstrating that mitochondrial targeting of PMPs occurs independently of PEX19 in peroxisome-deficient cells^40^, confirming that this pathway does not require PEX19.

Together, these findings provide strong evidence that PMPs are marked for degradation at the outer mitochondrial membrane, where MUL1 and MARCH5 reside, and that deletion of these E3 ligases leads to the accumulation of PMPs at the outer mitochondrial membrane. Previous studies have shown that ubiquitinated outer mitochondrial membrane proteins are extracted by AAA ATPases prior to degradation by the cytosolic proteasome^41,42^. Consistent with this model, confocal microscopy of *PEX3* KO cells expressing PMP-GFP reporters (PMP34 and PEX11A) and treated with the proteasome inhibitor bortezomib revealed cytoplasmic accumulation of the reporters (Supplementary Fig. 7a, c). Quantitative analysis confirmed this cytoplasmic localization, showing a significant increase in colocalization (Pearson’s r) between the PMP-GFP signal and DsRed, which serves as a nucleocytoplasmic marker and expression reference in the GPS system (Supplementary Fig. 7b, d). These observations suggest that a similar extraction and proteasome-dependent degradation mechanism applies to mitochondria-targeted PMPs. However, the identity of the ATPase responsible for this process remains to be determined. Notably, aggregates were observed in the cytoplasm of proteasome-inhibited, peroxisome-deficient cells, suggesting that interference with UPS-mediated clearance of PMPs may lead to their aggregation, presumably due to hydrophobic interactions driven by their TMDs. This underscores the importance of PQC of PMPs in preventing toxic accumulation upon mislocalization.

Finally, because protein-targeting machineries have limited capacity and are not entirely error-free, even under normal conditions^9,43^, we hypothesized that MARCH5 and MUL1 surveil mislocalized PMPs in cells with intact peroxisomes. To address this, we first analyzed the exogenously expressed PMP34-GFP reporter. Fluorescence microscopy revealed significantly increased mitochondrial co-localization of this reporter in *MARCH5/MUL1* DKO cells compared to control KO cells (Supplementary Fig. 8a,b), indicating that a fraction of overexpressed PMP is mistargeted to mitochondria and subsequently degraded by MARCH5/MUL1. These findings align with previous reports demonstrating mitochondrial mistargeting upon PEX14 overexpression^44^. Consistently, in most cases, a similar trend was observed: flow cytometry analysis showed an increase in PMP stability in cells lacking *MARCH5* and *MUL1*, even with intact peroxisomes (Fig. 3c, d). This underscores the role of this pathway in surveilling mislocalized PMP species.

To determine whether this phenomenon also occurs under physiological conditions, we examined endogenous PMPs using a CRISPR knock-in cell line in which GFP was fused to PMP70^45^, a representative PMP. In this background, we generated *MARCH5/MUL1* DKO cells and subsequently assessed the mitochondrial localization of the endogenous PMP. In contrast to the exogenous expression setting, no significant increase in mitochondrial localization was observed in *MARCH5/MUL1* DKO cells (Supplementary Fig. 8c, d). Consistently, cellular fractionation did not reveal elevated steady-state levels of PMPs in the mitochondria-enriched fraction of *MARCH5/MUL1* DKO cells (Fig. 4c).

Together, these results indicate that while mistargeting of PMPs to mitochondria can occur upon overexpression and is subject to MARCH5/MUL1-dependent degradation, endogenous PMPs are efficiently and faithfully targeted to peroxisomes under physiological conditions. We cannot exclude the possibility that a very small fraction of endogenous PMPs is mistargeted; however, if present, such events are below the detection limit of our microscopy and biochemical approaches.

### PMP TMD is sufficient for mitochondrial E3 ligases-dependent degradation

Our data show that PMPs degraded upon peroxisome loss belong to diverse classes, including single- and multi-pass proteins with different topologies. Since all are targeted by the same E3 ligases (MARCH5 and MUL1), they likely share a common recognition feature. Given their varied cytosolic domains, the most plausible shared element is the TMD. To test this idea, we used the single-pass, tail-anchored PMP PEX26 as a simplified model, as its TMD and short C-terminal sequences are sufficient for peroxisomal targeting^46^. In our MS analysis, PEX26 was degraded upon peroxisome loss (Supplementary Fig. 2c and Supplementary Data 1), which we confirmed using endogenous antibodies (Fig. 1d–g). Consistent with our findings for other PMPs, its degradation is dependent on MARCH5 and MUL1 (Fig. 3f, g).

**Fig. 5:**
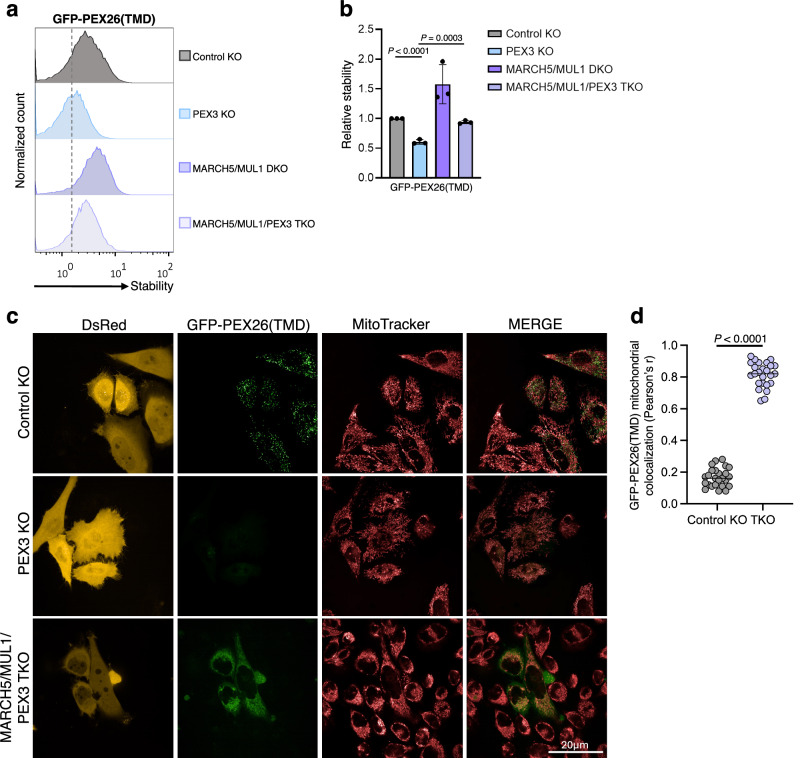
Transmembrane domains mediate recognition of PMPs by MARCH5 and MUL1. **a**,**b** Stability analysis of PEX26(TMD) GPS reporter in HEK293T control, *PEX3* KO, *MARCH5/MUL1* DKO, and *MARCH5/MUL1/PEX3* TKO cells. Representative flow cytometry histograms are shown in (**a**) and quantitative analysis (mean GFP/DsRed fluorescence normalized to control KO ± s.d.) from *n* = 3 biological replicates are shown in (**b**). *P* values were determined using a two-tailed unpaired *t*-test and are indicated in the figure. **c** Representative confocal fluorescence microscopy images of the GFP-PEX26(TMD) reporter expressed in the indicated genetic backgrounds of HeLa cells. Orange, DsRed expression control; green, GFP-PEX26(TMD) signal; red, mitochondrial marker MitoTracker. MERGE panels show the overlay of the GFP-PEX26(TMD) and MitoTracker signals. Scale bar, 20 µm. **d** Pearson’s correlation coefficient (Pearson’s r) analysis for the colocalization of GFP-PEX26(TMD) with the MitoTracker signal. Data are presented as individual data points with mean values; *n*  =  25 cells per genetic background. The *P* value was determined by a two-tailed unpaired *t*-test and is indicated in the figure. Source data are provided as a Source Data file.

To directly test whether the TMD mediates recognition, we generated a GPS reporter in which GFP is fused to the minimal TMD and C-terminal region of PEX26 (GFP-PEX26(TMD)) and assessed its stability by flow cytometry and subcellular localization by microscopy. GFP-PEX26(TMD) stability decreased in *PEX3* KO cells and was restored upon *MARCH5/MUL1* ablation (Fig. 5a, b). The reporter also showed increased mitochondrial co-localization in *MARCH5/MUL1/PEX3* TKO cells (Fig. 5c, d), consistent with mitochondrial localization driven by the TMD upon peroxisome loss, followed by MARCH5/MUL1-dependent degradation.

Together, these results demonstrate that the PEX26 TMD is sufficient to confer recognition by the MARCH5/MUL1 PQC pathway, directly supporting our hypothesis that membrane-embedded elements of PMPs mediate substrate recognition.

### *MARCH5/MUL1* ablation impairs survival in peroxisome-deficient cells

**Fig. 6:**
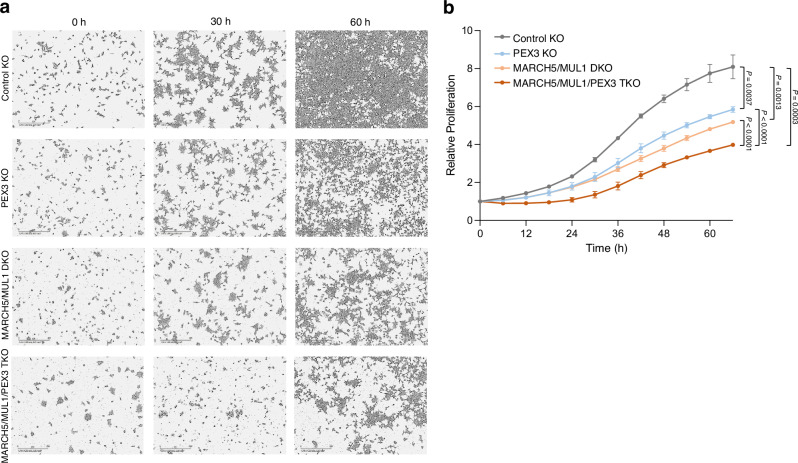
Mitochondrial quality control of PMPs supports cell survival upon peroxisome biogenesis failure. **a**,**b** Proliferation assay of control, *PEX3* KO, *MARCH5/MUL1* DKO, and *MARCH5/MUL1/PEX3* TKO cells. **a** Representative bright-field microscope images of each genotype at 30 h and 60 h post-seeding, showing differences in cell density and proliferation. **b** Quantification of proliferation as fold change in cell number relative to day 0, with each genotype normalized to its initial cell count. Mean ±  s.d. from *n* = 3 biological replicates are shown for each time point. *P* values from two-tailed unpaired *t*-tests are indicated in the figure. Source data are provided as a Source Data file.

To assess the impact of PMPs accumulation in mitochondria on cellular physiology, we performed a proliferation assay. We compared the proliferation of control KO cells with *PEX3* KO cells, *MARCH5/MUL1* DKO cells, and *MARCH5/MUL1/PEX3* TKO cells. During the course of the assay (total 66 hours), *PEX3* KO or *MARCH5/MUL1* DKO cells displayed a marked reduction in proliferation, which was further exacerbated in the *MARCH5/MUL1/PEX3* TKO cells (Fig. 6). These findings indicate a synergistic negative effect of peroxisome loss combined with the loss of mitochondrial E3 ligases, due in part to impaired mitochondrial turnover of PMPs. Altogether, these results highlight the critical role of MUL1 and MARCH5 as quality control factors for PMP degradation upon peroxisome loss.

## Discussion

**Fig. 7:**
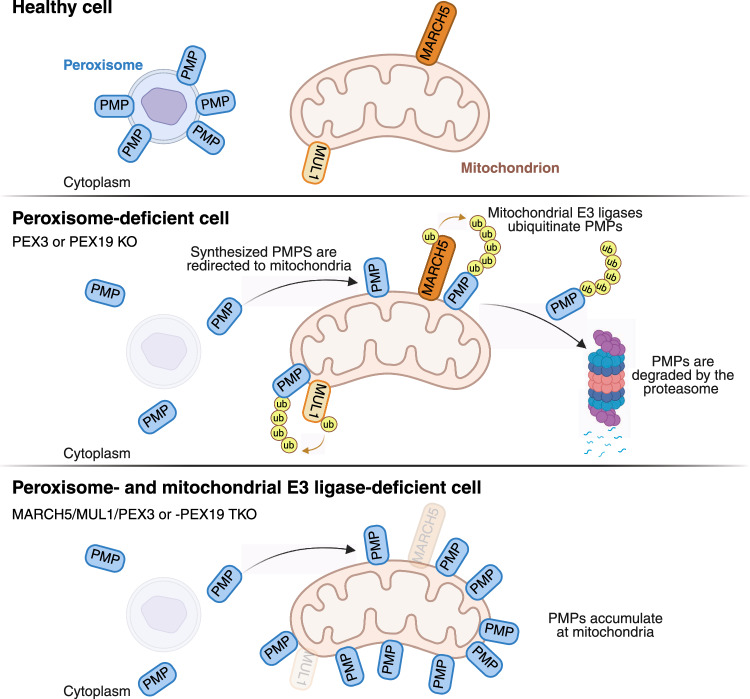
Model: mitochondria-mediated quality control of PMPs in peroxisome-deficient cells. In healthy cells, PMPs are correctly inserted into peroxisomes. Upon peroxisome biogenesis failure (*PEX3* or *PEX19* KO), newly synthesized PMPs are not degraded in the cytosol but are instead rerouted to mitochondria, where they are ubiquitinated by the mitochondrial E3 ligases MARCH5 and MUL1 and subsequently degraded by the proteasome. In cells lacking both peroxisomes and mitochondrial E3 ligases (*MARCH5/MUL1/PEX3* or -*PEX19* triple KO), PMPs accumulate on mitochondria, indicating a failure of their clearance. Created in BioRender. Koren, I. (2026) https://BioRender.com/nyuhpbq.

Recent studies have shown that mitochondria-localized MARCH5 is essential for the de novo biogenesis of peroxisomes from mitochondria in human cell lines; loss of MARCH5 impairs the budding of PEX3-containing vesicles from mitochondria, thereby blocking the formation of pre-peroxisomes^36,37^. Here we show that the mitochondrial E3 ligases MARCH5 and MUL1 also contribute to the PQC of PMPs that are rerouted to mitochondria in the absence of peroxisomes (Fig. 7). Thus, mitochondrial E3 ligases appear to play a dual role- facilitating peroxisome biogenesis under physiological conditions while promoting the degradation of mitochondria-directed PMPs when peroxisome formation is impaired. This functional flexibility reveals a previously unappreciated integration of organelle biogenesis and PQC at the mitochondrial surface. Several questions remain unresolved. It remains unclear how PMPs are shielded from mitochondrial E3 ligases under normal conditions when peroxisome biogenesis is intact, yet become susceptible to degradation in cells lacking or harboring mutations in key biogenesis factors. In addition, a subset of PMPs, such as PEX14, appears to be resistant to degradation, though the mechanisms underlying their protection remain to be elucidated. Moreover, certain PMPs, such as PEX11B and PEX16, are degraded independently of MARCH5 and MUL1, suggesting that additional E3 ligases act in parallel. Identifying these factors will be important for fully defining the organellar PQC network. Notably, while we demonstrate MARCH5/MUL1-dependent degradation of PMPs through genetic and functional assays, we do not directly assess physical interactions between the E3 ligases and PMPs due to the challenges associated with purifying membrane-embedded protein complexes, compounded by the transient nature of E3 ligase–substrate interactions.

Importantly, peroxisome-mitochondria communication has been documented in multiple contexts. In mammalian cells, these organelles cooperate in fatty acid β-oxidation and ROS metabolism, and several tethering proteins have been proposed to mediate their interaction^47,48^. However, our findings extend this view by suggesting that peroxisome-mitochondria interplay persists even when one organelle is absent. Given this tight functional relationship, it is not surprising that peroxisome loss leads to mitochondrial defects. Although PBDs are caused by pathogenic variants in *PEX* genes, mitochondria are also affected in these diseases^49^. This is likely due to disrupted metabolic pathways, ROS accumulation, and impaired mitochondrial fission^50–52^. Moreover, our results suggest that the accumulation of mitochondria-localized PMPs may represent an additional trigger contributing to mitochondrial dysfunction upon peroxisome loss.

While cytoplasmic PQC pathways handle mislocalized ER and mitochondrial proteins, we found that upon peroxisome biogenesis failure, PMPs are rerouted to mitochondria and targeted by mitochondrial E3 ligases. Mislocalized membrane proteins can induce proteotoxicity through various mechanisms, such as protein aggregation or inappropriate interactions with other proteins interfering with their function, underscoring the importance of efficient surveillance and removal. Given the limited capacity of protein-targeting machineries^9,43^, we hypothesized that a small fraction of PMPs might also be mistargeted to mitochondria under normal conditions and handled by MARCH5 and MUL1. However, we did not detect appreciable mitochondrial mislocalization of endogenous PMPs in *MARCH5/MUL1*-deficient cells, indicating that peroxisomal targeting is highly efficient and that any mistargeted species are below our detection limits. Importantly, mislocalization may be exacerbated by stress conditions or mutations in targeting sequences or biogenesis factors^53–56^. Indeed, mitochondrial mislocalization of a subset of PMPs due to mutations in peroxisome biogenesis factors has been shown to impair mitochondrial structure and function^39,57,58^. Similarly, overexpression of the PMP PEX14 leads to its mislocalization to mitochondria, accompanied by mitochondrial abnormalities^44^. Our study builds on these observations by identifying a UPS-dependent degradation mechanism mediated by mitochondrial E3 ligases, which actively remove mitochondria-targeted PMPs upon peroxisome loss. This pathway likely becomes particularly important when targeting fidelity is challenged, thereby maintaining proteostasis and helping to limit mitochondrial damage. Importantly, certain cell types, such as ZSD patient fibroblasts, mouse embryonic fibroblasts, and hepatocytes, are more sensitive to PMP mislocalization than others (e.g., cancer cell lines)^44,49,51^, suggesting that this variability may partly reflect differences in the expression or activity of mitochondrial E3 ligases responsible for degrading mislocalized PMPs.

It remains unclear whether the targeting of PMPs to mitochondria upon peroxisomal biogenesis failure is a passive consequence of the absence of their native targeting site, resulting in mistargeting and thus should be considered as mislocalized, or an active process whereby the proteins are deliberately rerouted to mitochondria to facilitate their degradation. The concept of rerouting for degradation has been described in yeast, where a process termed mitochondria as guardian in the cytosol (MAGIC) facilitates the clearance of misfolded proteins. In this pathway, misfolded cytosolic proteins translocate into mitochondria via import channels, where they are degraded by the matrix-localized Lon protease Pim1^59^. This mechanism helps dissolve cytosolic protein aggregates associated with mitochondria. Similar observations of cytosolic misfolded proteins in human mitochondria suggest that a MAGIC-like mechanism may also exist in higher organisms^59,60^.

Collectively, our work supports a quality control axis in which PMPs ectopically localized to mitochondria are targeted for ubiquitin-mediated degradation by MARCH5 and MUL1. These findings advance our understanding of organelle cross-talk, protein homeostasis, and the mechanisms that safeguard the cellular proteome under conditions of organelle dysfunction. By uncovering the pathways that govern PMP turnover, this study provides a foundation for future mechanistic and structural investigations into peroxisomal PQC and its integration with mitochondrial surveillance systems.

## Methods

### Cell culture

HEK293T (ATCC CRL-3216) and HeLa (a gift from Kimchi A., Weizmann Institute of Science) were grown in Dulbecco’s Modified Eagle’s Medium (DMEM) (Life Technologies, #41965039) supplemented with 10% fetal bovine serum (Thermo Fisher Scientific, #A5256801) and 100 U/mL penicillin/streptomycin (Thermo Fisher Scientific, #15140122).

### Transfection and lentivirus production

Lentivirus was generated by transfecting HEK293T cells (80% confluency) with the transfer vector and four plasmids encoding Gag-Pol, Rev, Tat, and VSV-G using PolyJet (SignaGen Laboratories, #SL100688). Media was changed 24 h post-transfection, and lentiviral supernatants were collected 24 h later. After centrifugation (800 x g, 5 min), the virus was stored at −80 °C. Target cells were transduced with the virus and 8 μg/ml hexadimethrine bromide (Polybrene) (Sigma-Aldrich, #TR-1003).

### Inhibitors

Bortezomib (APEXBio, #A2614), MLN4924 (Active Biochem #A-1139) and MLN7243 (Selleck Chemicals, #S8341) were used at a final concentration of 1 µM. Cycloheximide (ApexBio, #A8244) was used at a final concentration of 50 µg/ml.

### Plasmids

The open reading frames (ORFs) encoding CAT, PEX13, PEX11A, PEX11B, PMP24, PMP34, PEX3 and PEX19 were obtained in the form of entry clones from the Ultimate ORF Clone collection (Thermo Fisher Scientific, # HORF01). *PEX3* and *PEX19* were amplified by PCR to add a HA tag at their C-terminus and subsequently cloned into the pHAGE Lenti vector using HiFi DNA Assembly (New England Biolabs (NEB), #NEB-E2621L). The plasmid also encodes for Crimson to enable control of equal expression levels of the constructs (EF1α promoter-ORF-PGK promoter-Crimson)^61^. PEX13, PEX11A, PEX11B, PMP24, PMP34 were cloned upstream of GFP (ORF-GFP) in the GPS vector^62^ using HiFi DNA Assembly, while CAT was cloned downstream of GFP in the GPS vector by LR reaction^63^ (Gateway LR Clonase II Enzyme Mix, Thermo Fisher Scientific, #11791020) according to the manufacturer’s protocol. The PEX26 TMD with its flanking sequences (residues 237-305) was synthesized as a DNA oligonucleotide by GenScript (The amino acid is shown below, with the TMD sequence underlined):

RQLWDSAVSHFFSLPFKKSLLAALILCLLVVRFDPASPSSLHFLYKLAQLFRWIRKAAFSRLYQLRIRD*. The fragment was cloned downstream of GFP in the GPS vector^63^ using HiFi DNA Assembly. pRK5-HA-Ubiquitin was obtained from Addgene (#17608).

CRISPR/Cas9-mediated gene disruption was performed using the lentiCRISPR v2 vector (Addgene, #52961). Oligonucleotides encoding the top and bottom strands of the sgRNAs were synthesized (IDT), annealed, and cloned into the vector as described^64^.

Nucleotide sequences of the sgRNAs used were:

sg-AAVS1 (control): GGGGCCACTAGGGACAGGAT

sg1-PEX3: GATTAAGGCCTCTCTCAGTGT

sg2-PEX3: GCTGTCATATTGCAAGTCCTC

sg1-PEX19: GCTCCAATTCCCTGTCCGCTT

sg2-PEX19: GTGAGGAAGGCTGTAGTGTCG

sg-MUL1: GTACTCCGTGTACCGGCAGA

sg-MARCH5: GACCAGGCCTGTCTACAACGC

sg-BAG6: GCAAGATGATAAGAAGCTTC

sg-PEX2: GGACCAAACTAGCTGCTCCA

### Generation of CRISPR/Cas9 knockout cells

Lentivirus was generated by transfecting HEK293T with lentiCRISPR v2 encoding the relevant sgRNA as described above. Forty hours post-transduction, cells were selected with puromycin. Genomic DNA was extracted 7 days later, PCR-amplified (~500 bp flanking the edited site), and analyzed by Sanger sequencing. Editing efficiency was assessed using the Inference of CRISPR Edits (ICE) CRISPR Analysis Tool (Synthego Performance Analysis v3.0.).

*PEX3* or *PEX19* KO cells were generated by viral transduction using sg1-PEX3 and sg1-PEX19, followed by puromycin selection. For the proteomics experiments (Fig. 1a–c and Supplementary Fig. 2a–c) and RNA-seq experiment (Fig. 1h), single clones (PEX3 KO1 and PEX3 KO2) were obtained from the *PEX3* KO cell population. For all other experiments, validation was performed using the polyclonal KO cell populations, not single clones. For KO–rescue experiments (Supplementary Fig. 3b,c), *PEX3* and *PEX19* KO cells were generated by transfection using sg2-PEX3 and sg2-PEX19, respectively, followed by puromycin selection for 48 hours. *BAG6* KO cells were generated via viral transduction, after which PEX3 sg1-RNA was introduced to produce the corresponding double KO (DKO) cells. *MUL1* and *MARCH5* DKO cells were made via simultaneous transduction with both MUL1 and MARCH5 sgRNAs. For triple KO (TKO) cells, *MUL1/MARCH5* DKO populations were further transduced with sg1-PEX3 or sg1-PEX19 sgRNAs to generate the corresponding TKO cell populations.

### Rescue experiment with PEX3 and PEX19 cDNA

*PEX3* or *PEX19* KO cells were transduced with lentiviruses carrying *PEX3* or *PEX19* cDNA, respectively. The cDNA plasmid also encodes for Crimson which serves as a fluorescent marker for successful transduction and comparable expression levels of the constructs. 48 h post-transduction, cells expressing the cDNA were analyzed by flow cytometry to monitor Crimson expression as readout for successful integration of the cDNAs into the host genome.

### Global protein stability (GPS) assay

The GPS reporter assay was described previously^63^. To assess PMPs stability, lentiviral vector encoding the GPS reporters were introduced into control KO or the indicated KO cells, followed by selection for 3 days. Flow cytometry was performed on a CytoFLEX (Beckman Colter) to record GFP and DsRed fluorescence from at least 10,000 cells, and the GFP/DsRed ratio was analyzed using FlowJo v11. A schematic explaining the flow cytometry gating strategy is shown in Supplementary Fig. 3a. This gating strategy was used for all GPS assays presented in the manuscript, including Fig. 2c, Fig. 3c, Fig. 5a, Supplementary Fig. 3c,e, and Supplementary Fig. 4a,b.

### Immunoblotting

Cells were lysed in ice-cold lysis buffer (10 mM NaPO_4_, 100 mM NaCl, 5 mM EDTA pH 8, 1% Triton X-100, 0.5% Deoxycholic acid sodium salt, 0.1% SDS) supplemented with Halt Protease and Phosphatase Inhibitor Cocktail (Thermo Fisher Scientific, #78442) for 25 min at 4 °C. Lysates were clarified by centrifugation (20,000 x g, 15 min, 4 °C), and nuclear pellets were resuspended in lysis buffer, sonicated briefly, and re-clarified. Protein concentration was determined by a standard Bradford assay (Bio-Rad #500-0006), a linear bovine serum albumin (BSA) calibration curve, and an Epoch microplate spectrophotometer. Proteins were subsequently resolved by SDS-PAGE (Mini-PROTEAN TGX Precast Protein Gels, Bio-Rad) and transferred to a nitrocellulose membrane, which was then blocked in 10% nonfat dry milk in PBS + 0.1% Tween-20 (PBS-T). The membrane was incubated with primary antibody overnight at 4 °C, and then, following three washes with PBS-T, HRP-conjugated secondary antibodies goat anti-rabbit IgG (H + L) HRP (1:20,000, Jackson ImmunoResearch Labs, #111-035-144) or goat anti-mouse IgG (H + L) (1:20,000, Jackson ImmunoResearch Labs, #115-035-003) were added for 1 h at room temperature. Following a further three washes in PBS-T, reactive bands were visualized using SuperSignal West Femtochemiluminescence substrate (Pierce; #34095) or an EZ-ECL (Biological Industries; #20-500-171) for 5 min. Reactive bands were visualized using the ImageQuant TL software v8.2 on Amersham Imager 680 (Cytiva). Immunoblot quantification was carried out by measuring band intensities using Fiji ImageJ v1.54p, and statistical analyses were performed using GraphPad Prism v10.6.1.

The following primary antibodies were used: mouse anti-vinculin (1:1,000; Sigma-Aldrich, #V9264), rabbit anti-HA tag (1:1,000; Cell Signaling Technology (CST), #3724), rabbit anti-GFP tag (1:1,000; Abcam, ab290), rabbit anti-PEX3 (1:1,000; ABclonal, #A7352), rabbit anti-PEX19 (1:1,000; Proteintech, 14713-1-AP), rabbit anti-PEX14 (1:1,000; Bethyl, #A303-086A), rabbit anti-PEX13 (1:1,000; Proteintech, #26649-1-AP), rabbit anti-PEX2 (1:1,000; StJohns (STJ) #117503), mouse anti-PMP70 (1:1,000; Sigma-Aldrich, #SAB4200181), rabbit anti-MARCH5 (1:1,000; CST #19168), rabbit anti-MUL1 (1:1,000; Proteintech, #16133-1-AP), mouse anti-TOMM20 (1:1,000; Santa Cruz (SC) #17764), rabbit anti-BAG6 (1:1,000; CST #8523), rabbit anti-β-Tubulin (9F3) (1:1,000; CST #2128), rabbit anti-PEX26 (Proteintech #27472-1-AP), rabbit anti-PEX16 (Proteintech #14816-1-AP), rabbit anti- RNF187 (Novus Biologicals, #NBP1-91025), mouse anti-Catalase (R&D Systems, # MAB3398).

### In vivo ubiquitination analysis

HEK293T cells stably expressing GPS PMPs were grown in 10 cm plates, transfected with HA-ubiquitin and treated with bortezomib (1 µM, 5 h) 48 hours post-transfection. Cells were then lysed in ice-cold buffer with Halt Protease and Phosphatase Inhibitor Cocktail and 50 µM PR-619 (APEXBio Technology, #A8212) for 30 minutes on ice. Nuclei were pelleted by centrifugation (14,000 x g, 10 min, 4 °C). Protein concentration was measured by Bradford assay, and equal amounts (~1 mg) were incubated with 20 µl GFP-Trap Magnetic Agarose (Proteintech, #gtma) for 2 h. Beads were washed three times with stringent buffer (500 mM NaCl, 0.5 mM EDTA pH 8, Tris-HCl pH 7.5) containing Protease and Phosphatase Inhibitor Cocktail and PR-619 before proteins were eluted with SDS-PAGE sample buffer (95 °C, 10 min). SDS-PAGE and immunoblot were performed as described.

### Mitochondria purification

The mitochondria-enriched and mitochondria-depleted supernatant were isolated using the Mitochondria Isolation Kit (Abcam, #ab110170) as recommended by the manufacturer. Briefly, HEK293T cells were harvested and incubated in the supplied mild lysis buffer to preserve mitochondrial integrity for 10 min on ice. The cell suspension was then placed into a glass homogenizer and homogenized for 50 strokes using a tight pestle on ice. The homogenate was then centrifuged (600 x g, 10 min, 4 °C) to remove the nuclei and unbroken cells. The resulting supernatant was further centrifuged at 12,000 × g for 30 min at 4 °C to separate the mitochondria-enriched pellet from the mitochondria-depleted supernatant. Protein concentration was determined by the Bradford assay, and samples were analyzed by SDS-PAGE as described above.

### Microscopy

HEK293T cells expressing GFP-CAT grown on coverslips were fixed for 15 min with 4% formaldehyde. Following 3 washes with PBS-T, coverslips were mounted onto slides using mounting media (Sigma-Aldrich, #F6182) prior to imaging. Cells were imaged with Leica Stellaris 5 confocal microscope using LASX software, and a 63× oil lens /1.4 N.A. UPlanSApo objective (Olympus). For live cell mitochondria visualization, HEK293T or HeLa cells were plated on CellCarrier Ultra microplates (PerkinElmer) and stained with MitoTracker Deep Red FM (Cell-Signaling, #8788) for 30 min at 37 °C followed by 10 min incubation of 10 μg/ml Hoechst (Thermo Scientific, #62249) staining to label nuclei. For live cell peroxisome visualization, PeroxiSPY650 reagent^24^ (Spirochrome, #SC507) was used according to the manufacturer’s instructions. Briefly, cells were incubated with 1 μM reagent in culture medium for 15 min at 37 °C, followed by 10 min incubation of 10 μg/ml Hoechst, and subsequently imaged using standard Cy5 settings. Images were acquired using an Opera Phenix Plus High-Content Screening System (PerkinElmer) in confocal mode, capturing a minimum of nine fields per well with 63× water immersion objectives.

### Pearson’s correlation coefficient analysis

Colocalization between MitoTracker or DsRed and PMP-GFP was analyzed using the Coloc2 plugin in ImageJ. The multi-color fluorescence image was split into channels and converted to 8-bit for analysis. The Coloc2 plugin was used to assess colocalization between two channels, and Pearson’s correlation coefficient was used for statistical analysis. Statistical analysis was performed using GraphPad Prism 10 v10.6.1.

### Peroxisomes quantification

Images containing the PeroxiSPY650 reagents across different genetic backgrounds were acquired using an Opera Phenix Plus High-Content Screening System (PerkinElmer) in confocal mode. Image analysis was performed using Harmony high-content analysis software (Revvity). Nuclei were first detected based on Hoechst signal, and cell boundaries were defined using the cytoplasmic background fluorescence from the PeroxiSPY650 channel. Puncta were identified using a spot detection algorithm with defined intensity and size thresholds applied uniformly across all samples. The number of puncta per cell was calculated automatically by assigning detected puncta to individual segmented cells. Fifty-two fields per condition were captured, and the number of puncta was quantified for each individual cell, generating single-cell measurements across all imaged fields. These per-cell values were used to calculate summary statistics (e.g., mean puncta per cell) for each genetic background or condition. Data are presented as mean ± s.e.m., and statistical significance was determined using a two-tailed Welch’s *t*-test.

### Cell Proliferation Assay

HEK293T control KO, *PEX3* KO, *MARCH5/MUL1* DKO, and *MARCH5/MUL1/PEX3* TKO cells were seeded at 5 × 10⁴ cells per well in 24-well plates (triplicate for each background), and cell proliferation was monitored using the IncuCyte® S3 Live-Cell Analysis System (Sartorius). Phase-contrast images were acquired every 6 h over a 66-hour period, capturing 16 fields per well. Cell confluence was quantified automatically by the IncuCyte software (version 2023B), which measures the percentage of the imaged area occupied by cells. For each background, the mean confluence across triplicates was calculated and normalized to the initial time point (0 h). Relative proliferation was plotted as fold change over time. Statistical analyses were performed using GraphPad Prism 10 software, applying a two-tailed unpaired t-test to assess significance between groups.

### Proteomics

#### Cell lysis and protein digestion

The dataset includes three independent biological replicates of control KO cells and two independent *PEX3* KO HEK293T clones. Cells were lysed in 8.5 M Urea, 400 mM ammonium bicarbonate and 10 mM DTT, sonicated twice (90%, 10-10, 5’), and centrifuged (10,000 g, 10’). Protein amount was estimated using Bradford assay. The samples were reduced (60 °C for 30 min), modified with 35.2 mM iodoacetamide in 100 mM ammonium bicarbonate (room temperature for 30 min in the dark) and digested in 1.5 M Urea, 66 mM ammonium bicarbonate with modified trypsin (Promega), overnight at 37 °C in a 1:50 (M/M) enzyme-to-substrate ratio. An additional second digestion with Trypsin was done for 4 h at 37 °C in a 1:100 (M/M) enzyme-to-substrate ratio. The tryptic peptides were desalted using Oasis HLB 96-well µElution Plate (Waters), dried and re-suspended in 0.1% Formic acid in 2% acetonitrile.

#### Mass spectrometry analysis

The resulting peptides were analyzed by LC-MS/MS using an Exploris 480 mass spectrometer (Thermo) fitted with a capillary HPLC (Vanquish, Thermo Scientific).

The peptides were loaded in solvent A (0.1% formic acid in water) on an ionoptics, AUR3-25075C18-XT, 25 cm x 75 um ID, 1.7um C18 reversed phase analytical column. The peptides mixture was resolved with a 6 to 34% linear gradient of solvent B (80% acetonitrile with 0.1% formic acid) for 120 min followed by a 0.1 min increase of 34 to 99% and 11 min at 99% solvent B at flow rates of 0.15 μl/min. Mass spectrometry was performed in a positive mode using repetitively full MS scan (m/z 380-985, resolution 120,000) followed by DIA scans (10 Da isolation windows with 1 m/z overlap, and resolution 30,000).

#### Data analysis

The mass spectrometry data was analyzed using the DIA-NN software version 1.9.2^65,66^ against human proteome from the Uniprot database (20662 entries, downloaded January 2025), with Min peptide length set to 7, Maximum number of missed cleavages set to 1, Cysteine carbamidomethylation enabled as a fixed modification, and protein N-term acetylation enabled as a variable modification.

Peptide- and protein-level false discovery rates (FDRs) were filtered to 1%.

Statistical analysis of the identification and quantification results was done using Perseus 1.6.7 software^67^.

Supplementary Data 1 lists the MS and log_2_MS signal of all quantified proteins, as well as the differences between control KO and the two independent PEX3 KO clones, each analyzed in triplicate.

### RNA-seq library preparation and analysis

*Library preparation:* A total of 1 × 10^6^ cells from each cell line was grown in one well of a 6-well plates, in triplicates. Total RNA was isolated following cells lysis on dishes using Direct-zol RNA Microprep Kit (Zymo Research, #R2061), followed by cDNA synthesis with qScript cDNA Synthesis Kit (Quantabio, #95047-025). A quantity of 1 μg of total RNA was used for mRNA purification with the NEBNext poly(A) mRNA magnetic isolation module. NEBNext Ultra II directional RNA library preparation kits for Illumina were used for RNA-seq library preparation. NEBNext multiplex oligos for Illumina were used for indexing during PCR amplification of the final libraries. Libraries were quantified by qPCR using the NEBNext library quantification kit for Illumina and multiplexed accordingly. The libraries were sequenced on an Illumina NextSeq 2000 platform in a single-end sequencing format.

*RNA-Seq analysis*: RNA-Seq data quality was assessed using FastQC (v0.12.1) (Available online at: http://www.bioinformatics.babraham.ac.uk/projects/fastqc/). Sequencing reads were aligned to the human reference genome (GRCh38, annotation release 111) using STAR aligner (v2.7.10b) with default parameters. Alignment quality was evaluated based on the percentage of mapped reads. Gene-level quantification of aligned reads was performed using featureCounts (version 2.1.1)^68^, and the number of successfully assigned reads per gene was recorded. Samples generated between 21 and 44 million reads, with genome alignment rates ranging from ~74 to 92%, indicating high-quality mapping. Assignment of sequencing reads to annotated genomic features using featureCounts resulted in mapping rates ranging from 69 to 74% across all samples.

Normalization of read counts and differential expression analysis between control KO (WT) and *PEX3* KO samples were conducted using the DESeq2 R package^69^ (version 1.46.0), applying its standard workflow. The dataset includes duplicate (WT) samples and triplicates of two independent *PEX3* KO clones. The two WT samples showed a high degree of correlation (R² = 0.9452) and were therefore considered sufficient for comparison. *P* values were adjusted for multiple testing using the Benjamini-Hochberg method to control the false discovery rate (FDR). Genes with an adjusted *p*-value (padj) <0.05 and an absolute log2 fold change > 1 were considered significantly differentially expressed. Principal component analysis (PCA) was performed on variance-stabilized transformed expression data using DESeq2 to visualize sample clustering and variance between conditions.

RNA-seq results are presented in Supplementary Data 2.

### CRISPR screens

A custom sgRNA library was designed targeting 880 human E3s at a depth of 6 sgRNAs per gene^15^. The sgRNA library DNA was packaged into lentiviral particles. HEK293T cells stably expressing PMP-GFP fusion proteins were transduced at a multiplicity of infection of ~0.3 at a sufficient scale to maintain at least 1000-fold representation of the library. Untransduced cells were eliminated through puromycin selection commencing two days post-transduction. The top ~5% of the GFP/DsRed ratio was isolated by FACS, which was performed 7 days post-transduction. For each screen, genomic DNA was extracted from both the sorted cells and the unsorted library as a reference. The sgRNAs in both pools were amplified by PCR and sequenced on an Illumina NextSeq instrument.

#### CRISPR screens Analysis

Illumina reads were first trimmed of constant regions derived from the backbone of lentiCRISPR v2 expression vector using Cutadapt^70^. Count tables were generated from the remaining variable sgRNA sequences using Bowtie 2^71^. The Model-based Analysis of Genome-wide CRISPR/Cas9 Knockout (MAGeCK) method^32^ was used to rank the performance of individual genes targeted by multiple sgRNAs enriched in the sorted cells versus the unsorted populations. The full MAGeCK results for each screen are presented in Supplementary Data 3.

### Functional enrichment analysis

Functional enrichment analysis was performed using Metascape^25^. Significantly downregulated proteins from the proteomic dataset were compared against the total proteome background using the Gene Ontology (GO) Biological Processes database in Metascape. The ten most significantly enriched pathways, ranked by -log₁₀(*P* values), were visualized and are presented in Fig. 1c. The same parameters were applied to compare significantly downregulated non-peroxisomal proteins to the total proteome (Supplementary Fig. 2d).

### Transmembrane domain hydrophobicity analysis

All protein sequences were retrieved from UniProtKB. ER membrane proteins and PMPs were filtered based on Subcellular location feature annotation. Transmembrane domains (TMDs) were extracted for each protein using the Transmembrane feature annotations, resulting in 219 TMDs for PMPs and 9,357 TMDs for ER proteins. The start-end positions of each TMD were used to extract the corresponding amino acid sequence from the full protein, and their hydrophobicity scores were calculated using the Grand Average of Hydropathy (GRAVY) index^72^. The distributions of hydrophobicity for both membrane types were visualized using overlapping density histograms with 30 bins. Statistical comparison between PMPs and ER transmembrane proteins was performed using Welch’s two-sample *t*-test.

### Statistics and reproducibility

All experiments were independently repeated at least three times (biological replicates), unless otherwise specified. Representative examples of flow cytometry, microscopy, and immunoblotting data are shown, and similar results were consistently obtained across independent experiments.

### Reporting summary

Further information on research design is available in the Nature Portfolio Reporting Summary linked to this article.

## Supplementary information

Supplementary Information

Description of Additional Supplementary Files

Supplementary Data 1

Supplementary Data 2

Supplementary Data 3

Reporting Summary

Transparent Peer Review file

## Source data

Source Data

## Data Availability

The MS proteomic data generated in this study have been deposited to ProteomeXchange via the PRIDE database under the identifier PXD069489. The RNA-seq data generated in this study have been deposited to the NCBI GEO with accession number GSE310531. All data generated and analyzed in this study are included as Figures, Supplementary Figs. or Supplementary Data 1-3. Source data are provided with this paper.
