# Supplementary material for: A mitochondria-driven quality control mechanism for peroxisomal membrane proteins
Source: Nat Commun. 2026 Jun 10;17:7375. doi: 10.1038/s41467-026-74117-6 (PMC13402316; doi:10.1038/s41467-026-74117-6)
Supplement: Supplementary file 1 — Supplementary Information [file 41467_2026_74117_MOESM1_ESM.pdf]

Supplementary Information for

A Mitochondria-Driven Quality Control Mechanism for Peroxisomal Membrane Proteins

Sarin Segev-Nakar[1], Itay Koren[1,*]

[1]The Mina and Everard Goodman Faculty of Life Sciences, Bar-Ilan University, Ramat-Gan 5290002, Israel.

[*]To whom correspondence should be addressed. E-mail: Itay Koren, itay.koren@biu.ac.il.

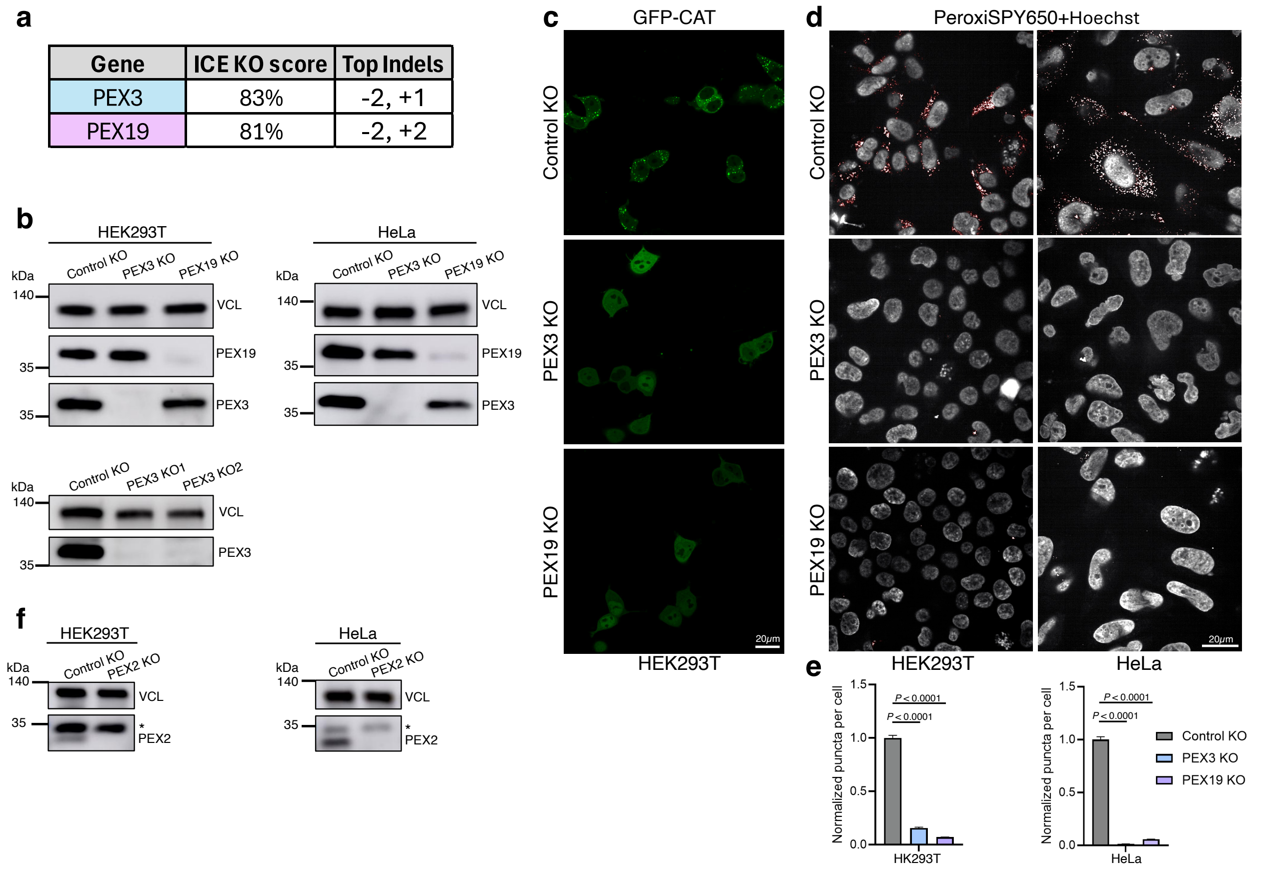

Supplementary Fig. 1. **Validation of peroxisome biogenesis factor knockout cells.**

(a) Knockout (KO) efficiency in *PEX3*- and *PEX19*-deficient cell populations. KO scores were calculated using the ICE CRISPR analysis tool, and the most abundant insertions and deletions (indels) are indicated.

(b) Confirmation of successful KO of *PEX3* and *PEX19* in HEK293T (top left) and HeLa (top right) cell populations by immunoblotting using antibodies to PEX3 or PEX19. The bottom panel shows two individual KO clones of HEK293T cells derived from the population. Vinculin (VCL) serves as a loading control.

(c) Representative immunofluorescence images of control cells (sgRNA targeting *AAVS1*) and *PEX3* or *PEX19* KO cells expressing the peroxisomal reporter GFP-fused Catalase (GFP-CAT). GFP-CAT shows punctate localization in control KO cells whereas it is diffuse in the cytoplasm of *PEX3* or *PEX19* KO cells, indicative of peroxisome loss. The experiment was independently repeated three times with similar results.

(d-e) Confocal fluorescence microscopy analysis showing loss of PeroxiSPY650 (peroxisome-specific reagent) signal in live HEK293T (left) and HeLa (right) *PEX3* or *PEX19* KO cells compared to control cells. The experiment was independently repeated three times with similar results. Representative overlaid images are shown in **(d)**. Red, PeroxiSPY650; nuclei were stained with Hoechst. Scale bar, 20 μm. **(e)** Quantification of PeroxiSPY650 puncta per cell in *PEX3* or *PEX19* KO cells compared to control cells. $n = 1965$ (control KO), $n = 1786$ (*PEX3* KO), and $n = 5127$ (*PEX19* KO) cells for HEK293T; $n = 2266$ (control KO), $n = 2267$ (*PEX3* KO), and $n = 2372$ (*PEX19* KO) cells for HeLa cells. Data are presented as mean ± s.e.m. *P* values were determined using Welch's two-sided *t*-test and are indicated in the figure.

(f) Immunoblot analysis of PEX2 in control or *PEX2* KO HEK293T cells (left) and HeLa cells (right) using an anti-PEX2 antibody. VCL serves as a loading control. The absence of the lower band in KO cells indicates that this band corresponds to PEX2, whereas the upper band persists and is therefore considered non-specific.

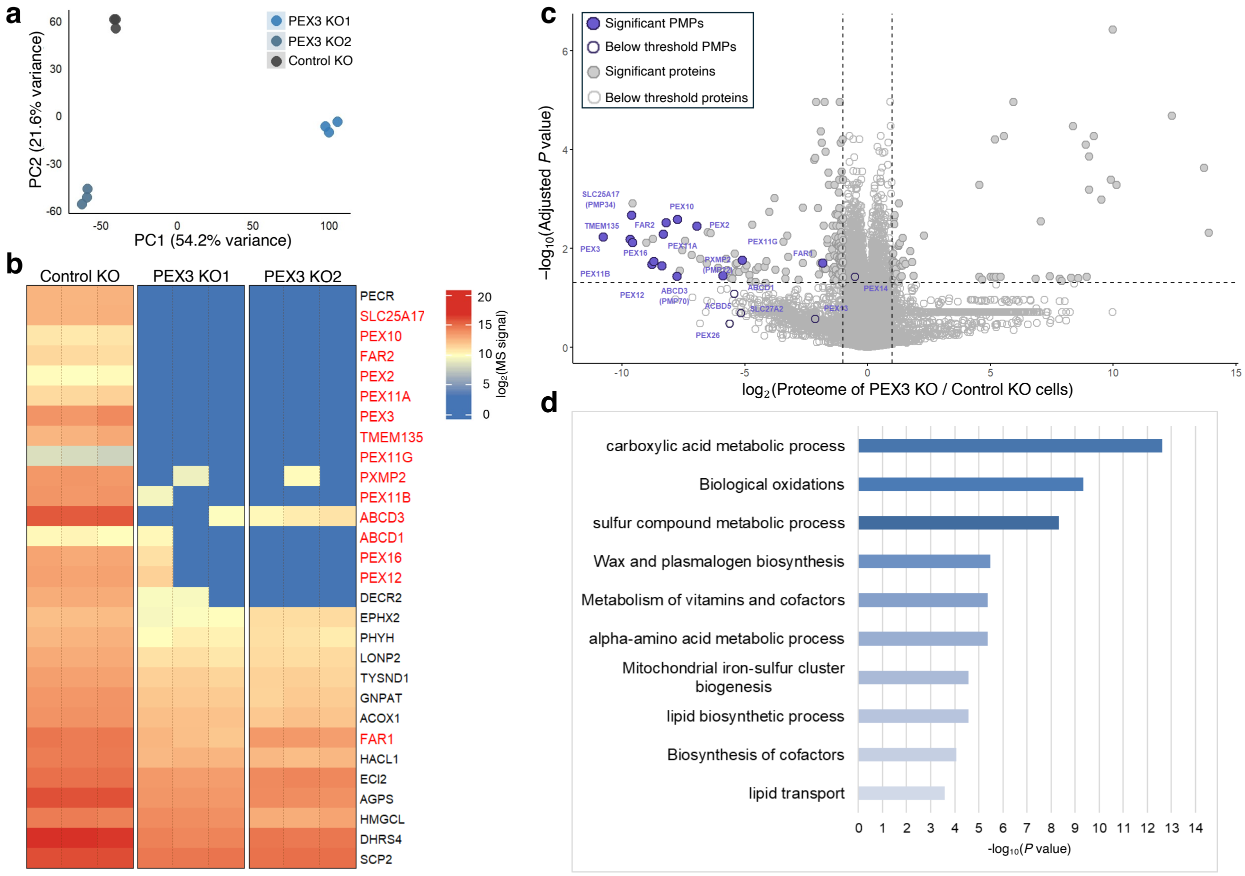

Supplementary Fig. 2. **Proteomic changes in *PEX3* knockout cells.**

(a) Principal component analysis (PCA) of label-free mass spectrometry (MS) data showing distinct proteomic profiles between HEK293T control KO (sgRNA targeting *AAVS1*) and two independent *PEX3* KO clones.

(b) Heatmap showing MS-based proteomic abundance changes of significantly downregulated peroxisomal proteins relative to control KO across all replicates. Peroxisomal membrane proteins (PMPs) are highlighted in red.

(c) Volcano plot displaying the $\log_2$ fold change (FC) versus $-\log_{10}(P \text{ value})$ from MS proteomics analysis of the total proteome, comparing control KO cells to two independent *PEX3* KO cells. Significance thresholds (dashed lines) were set at $\log_2 FC \geq 1$ or ≤ -1 and adjusted $P < 0.05$, determined by Welch's *t*-test. Light gray circles represent non-PMPs, with gray filled circles indicating proteins with significant abundance changes and empty gray circles representing proteins not meeting fold-change or statistical thresholds. PMPs are highlighted in purple and labeled with their gene names: empty circles denote PMPs not meeting significance, and filled circles indicate significantly downregulated PMPs.

(d) Functional enrichment analysis of significantly downregulated non-peroxisomal proteins. Statistical significance of enrichment was determined by Metascape.

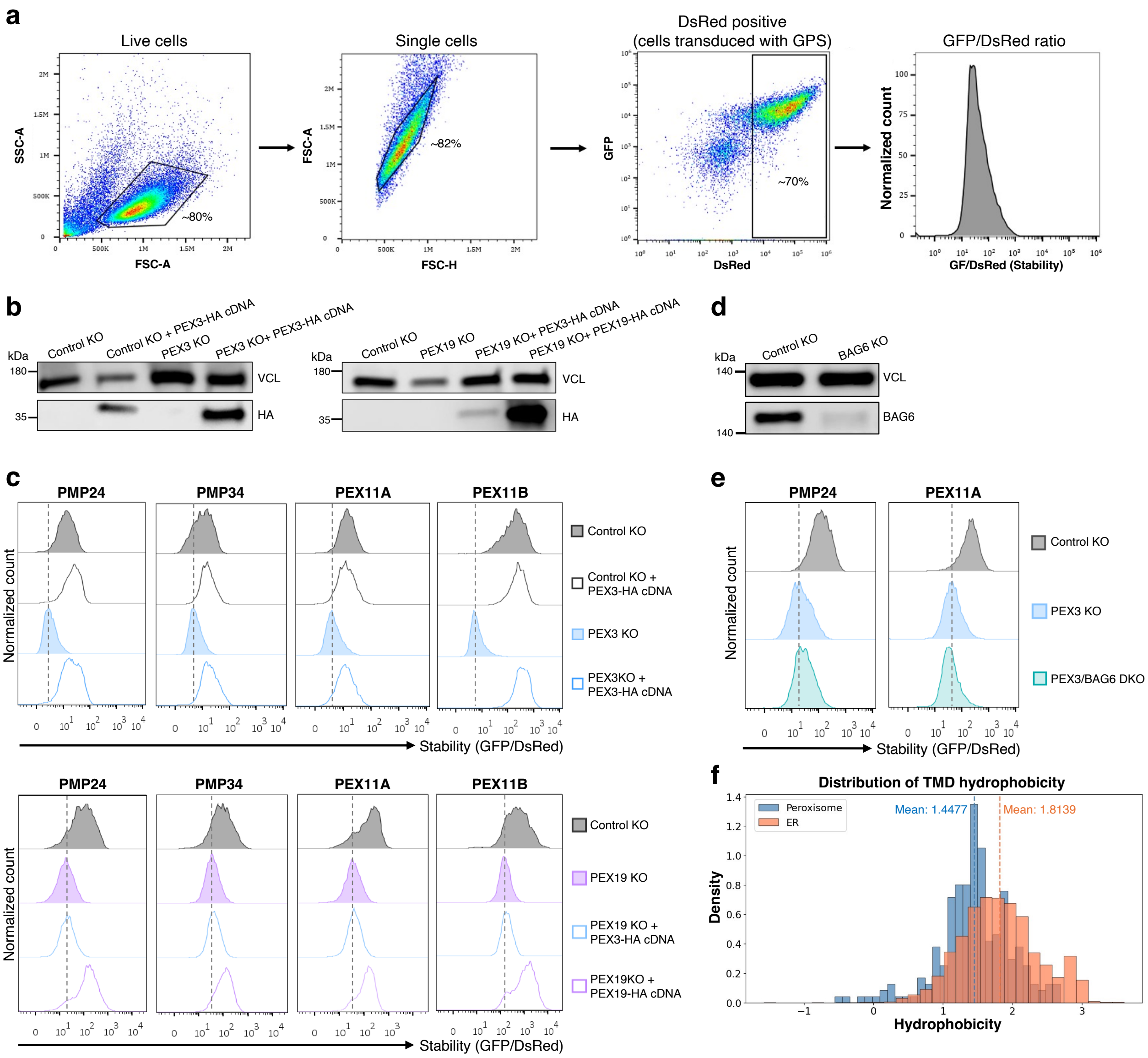

Supplementary Fig. 3. **Characterization of the PMP degradation pathway.**

(a) Flow cytometry gating strategy for the GPS assays. Live cells were gated based on FSC-A vs. SSC-A, and single cells were subsequently gated using FSC-A vs. FSC-H. A minimum of 10,000 DsRed-positive cells (representing successful lentiviral integration of the GPS reporters) were analyzed using FlowJo software to determine the GFP/DsRed ratio, which reflects the stability of the GFP-fused substrate.

(b-c) *PEX3* or *PEX19* KO cells stably expressing PMP-GFP reporters were transduced with the corresponding *PEX3* or *PEX19* cDNA. **(b)** Immunoblot with an anti-HA antibody confirming successful expression of the introduced cDNAs. **(c)** Flow cytometry analysis of GPS reporter stability in KO cells with or without cDNA expression. The dashed line marks the peak of the *PEX3* or *PEX19* KO condition to highlight the rightward shift (rescue from degradation) upon cDNA introduction. The experiment was independently repeated three times with similar results.

(d) Immunoblot confirming the successful KO of *BAG6* using an anti-BAG6 antibody. VCL serves as a loading control. *BAG6* KO cells were subsequently used to generate *PEX3/BAG6* double KO

(DKO) cells via transduction with a PEX3-targeting sgRNA (sg1). *PEX3* KO was confirmed by sequencing.

(e) Flow cytometry analysis of PMP GPS reporter stability in *PEX3* or *PEX3*/*BAG6* DKO cells. The dashed line marks the peak of the *PEX3* KO cells to demonstrate that the additional KO of *BAG6* does not induce a stabilization shift. The experiment was independently repeated three times with similar results.

(f) Distribution of transmembrane domain (TMD) hydrophobicity comparing ER membrane proteins (orange, mean = 1.8139) and PMPs (blue, mean = 1.4477), showing that PMP TMDs are significantly less hydrophobic ($P < 0.001$, Welch's two-sample t-test).

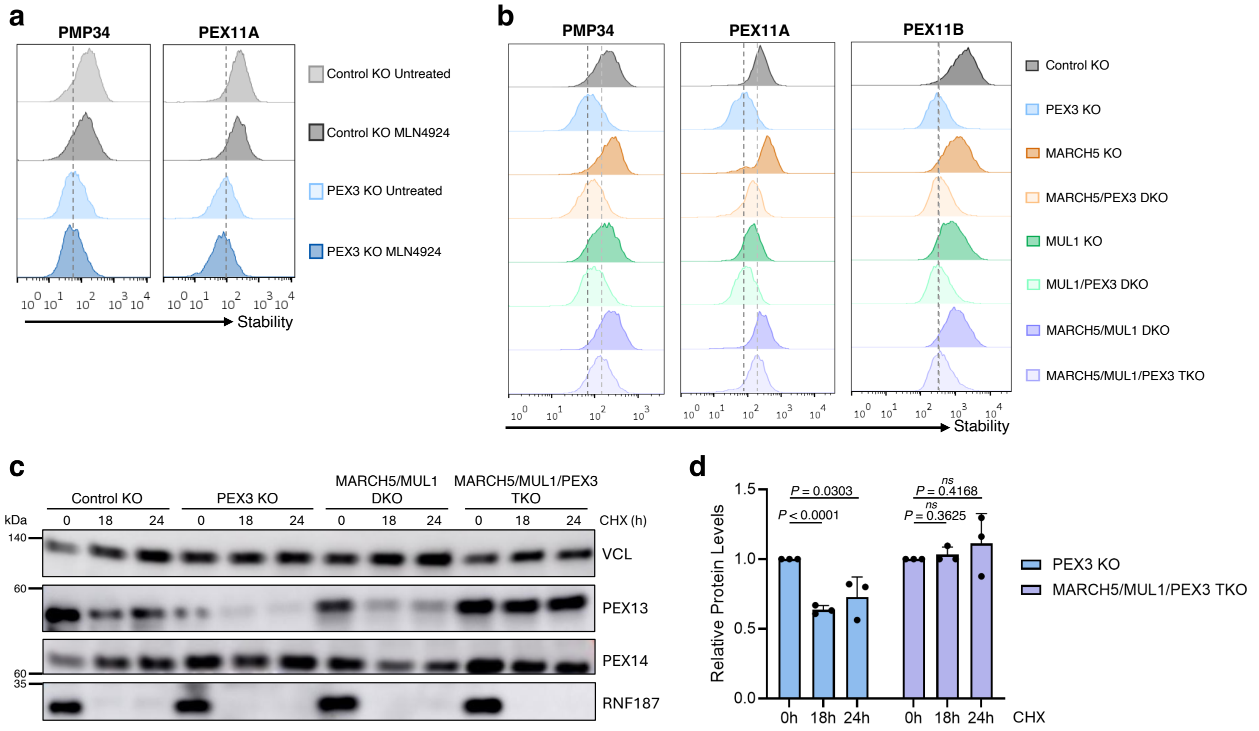

Supplementary Fig. 4. **MUL1 and MARCH5 redundantly promote PMP degradation.**

(a) Stability analysis of PMP GPS reporters in control or *PEX3* KO cells treated with 1 µM of the Cullin-RING ligase inhibitor MLN4924 for 10 h, as assessed by flow cytometry. The dashed line marks the peak of untreated *PEX3* KO cells, demonstrating that MLN4924 treatment does not induce a stabilization shift. The experiment was independently repeated three times with similar results.

(b) Flow cytometry-based stability analysis of the indicated PMP GPS reporters in control or *PEX3* KO cells with individual or combined ablation of *MUL1* and *MARCH5*. The dark gray dashed line marks the peak of *PEX3* KO cells and serves as a reference to assess stabilization shifts upon individual KO of *MUL1* or *MARCH5*. The light gray dashed line marks the peak of *MARCH5/MUL1/PEX3* TKO cells, demonstrating that this genetic background produces the most pronounced stabilization of PMP GPS reporters. PEX11B, a non-substrate of MUL1 and MARCH5, does not exhibit altered stability upon either individual or combined KO. The experiment was independently repeated three times with similar results.

(c-d) Cycloheximide (CHX) chase assay in the indicated KO cells treated with 50 µg/ml CHX for the specified time points, followed by immunoblot analysis of PEX13 and PEX14 protein levels. VCL serves as a loading control and RNF187 serves as a short-lived positive control to confirm CHX treatment efficacy. A representative immunoblot is shown in **(c)**. Quantitative analysis of relative PEX13 protein levels, normalized to VCL and the 0 h time point is shown in **(d)**. Data represent mean ± s.d. from $n = 3$ biological replicates. Significance was determined using a two-tailed unpaired t-test comparing the indicated time points to 0 h for each genetic background. P values are indicated in the figure. ns, not significant.

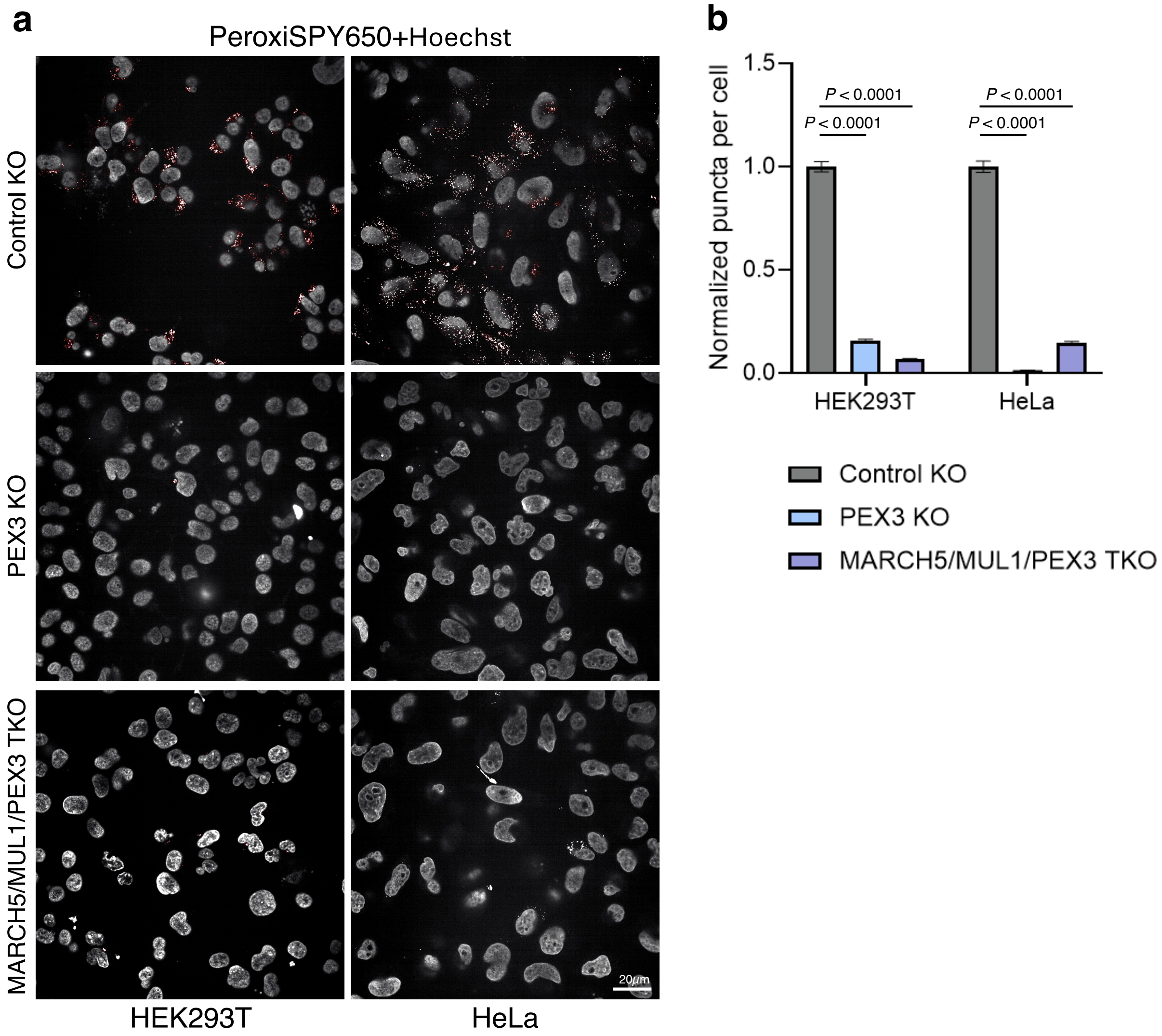

Supplementary Fig. 5. **Validation of peroxisome loss in *PEX3*-deficient cells.**

(a) Representative confocal fluorescence microscopy images showing loss of PeroxiSPY650 signal in live HEK293T (left) and HeLa (right) *PEX3* KO and *MARCH5/MUL1/PEX3* triple KO (TKO) cells compared to control KO cells. The experiment was independently repeated three times with similar results. Merged images are shown. Red, PeroxiSPY650; nuclei were stained with Hoechst. Scale bar, 20 µm.

(b) Quantification of PeroxiSPY650 puncta per cell, normalized to control cells, to assess relative peroxisome abundance in HEK293T (left) and HeLa (right) *PEX3* KO and *MARCH5/MUL1/PEX3* TKO cells. $n = 1965$ (control KO), $n = 1786$ (*PEX3* KO), and $n = 3871$ (TKO) cells for HEK293T; $n = 2266$ (control KO), $n = 2267$ (*PEX3* KO), and $n = 1784$ (TKO) cells for HeLa cells. Data are presented as mean ± s.e.m. P values were determined using Welch's two-sided t-test and are indicated in the figure.

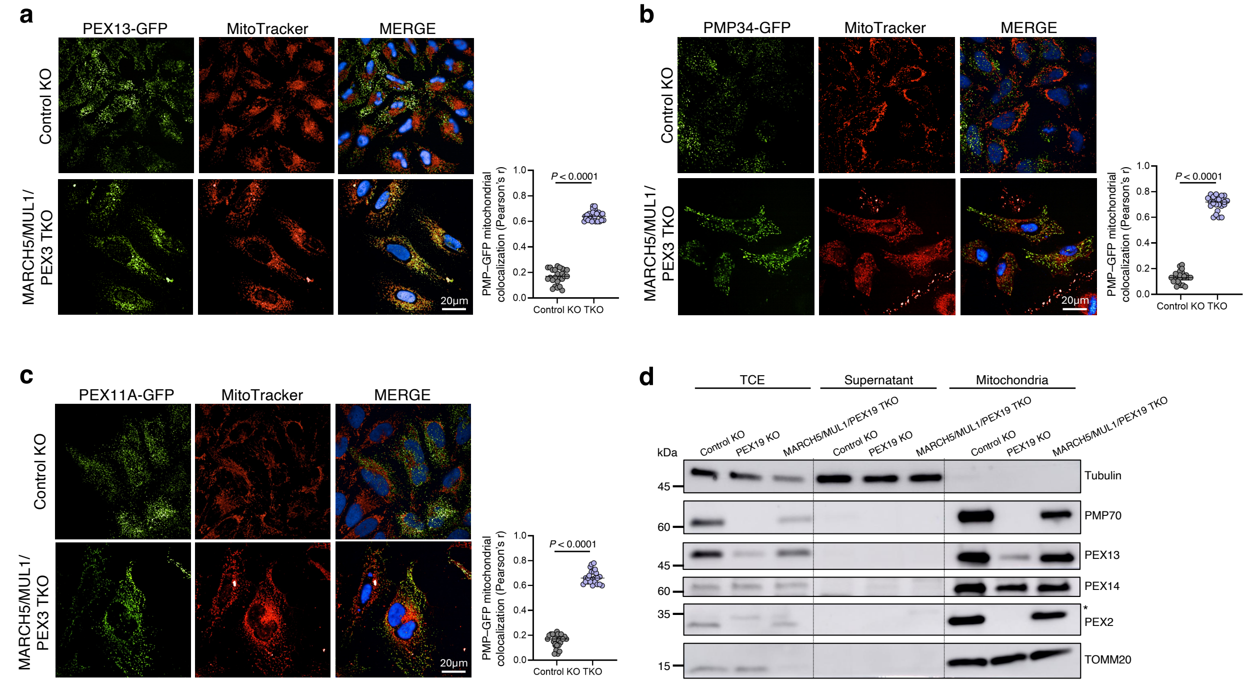

Supplementary Fig. 6. **Peroxisome loss redirects PMPs to mitochondria for MARCH5/MUL1-dependent degradation.**

(a-c) Representative confocal fluorescence microscopy images of PEX13-GFP **(a)**, PMP34-GFP **(b)**, and PEX11A-GFP **(c)** reporters in the indicated genetic backgrounds of HeLa cells. Green, PMP-GFP signal; red, mitochondrial marker MitoTracker. MERGE panels show the overlay of PMP-GFP, MitoTracker, and Hoechst nuclear stain (blue). Scale bar, 20 µm.

Pearson's correlation coefficient (Pearson's r) analysis for the colocalization of PMP-GFP from **(a-c)** with MitoTracker is shown in the graphs adjacent to the images, demonstrating increased colocalization in cells deficient for both *PEX3* and *MARCH5/MUL1* (*MARCH5/MUL1/PEX3* TKO). Data are presented as individual data points with mean values; $n = 25$ cells per genetic background. The P value was determined by a two-tailed unpaired t-test and is indicated in the figure.

(d) Cellular fractionation of the indicated KO cells followed by immunoblotting for endogenous PMPs in the mitochondria-enriched fraction (Mitochondria) and the mitochondria-depleted supernatant (Supernatant). TOMM20 and Tubulin serve as mitochondrial and supernatant protein loading controls, respectively. * denotes a non-specific band observed in PEX2 blots. The experiment was independently repeated three times with similar results.

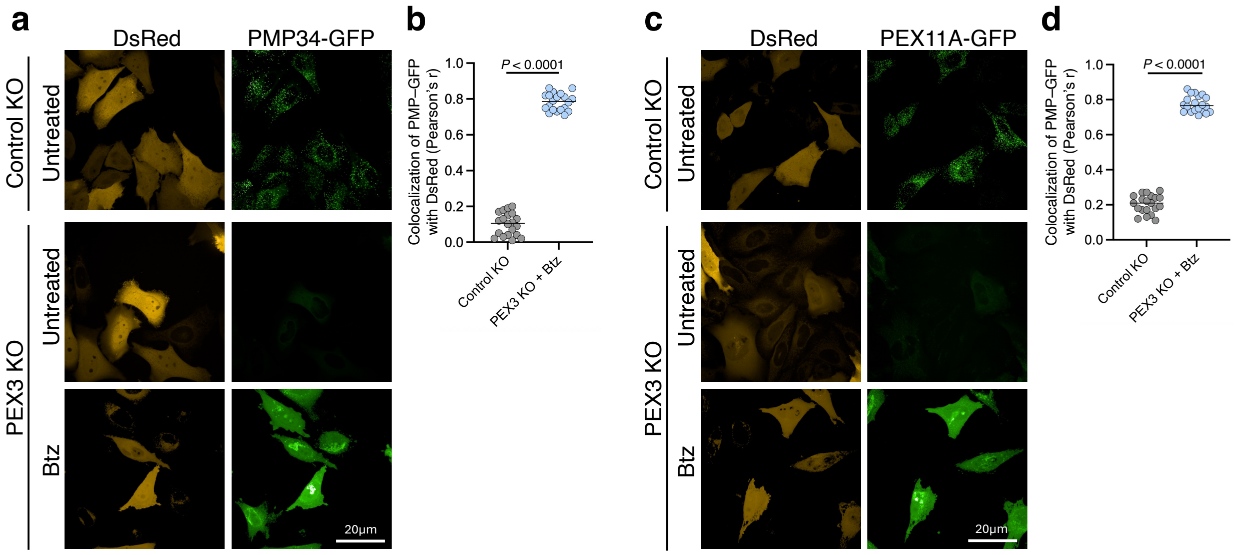

Supplementary Fig. 7. **Proteasome inhibition leads to cytoplasmic accumulation of exogenous PMP-GFP reporters.**

(a-d) The indicated exogenous PMP-GFP reporters were expressed in control or *PEX3* KO HeLa cells, either left untreated or treated with the proteasome inhibitor bortezomib (Btz; 1 µM, 16 h).

(a,c) Representative confocal fluorescence microscopy images for PMP34-GFP **(a)** and PEX11A-GFP **(c)**. Green, PMP-GFP signal; orange, DsRed expression control. Scale bar, 20 µm.

(b,d) Pearson's correlation coefficient (Pearson's r) analysis of the colocalization between PMP-GFP and DsRed, serving as a nucleocytoplasmic marker, for the conditions shown in **(a)** and **(c)**. Data are presented as individual data points with mean values; $n = 20$ cells per condition. The P value was determined by a two-tailed unpaired t-test and is indicated in the figure.

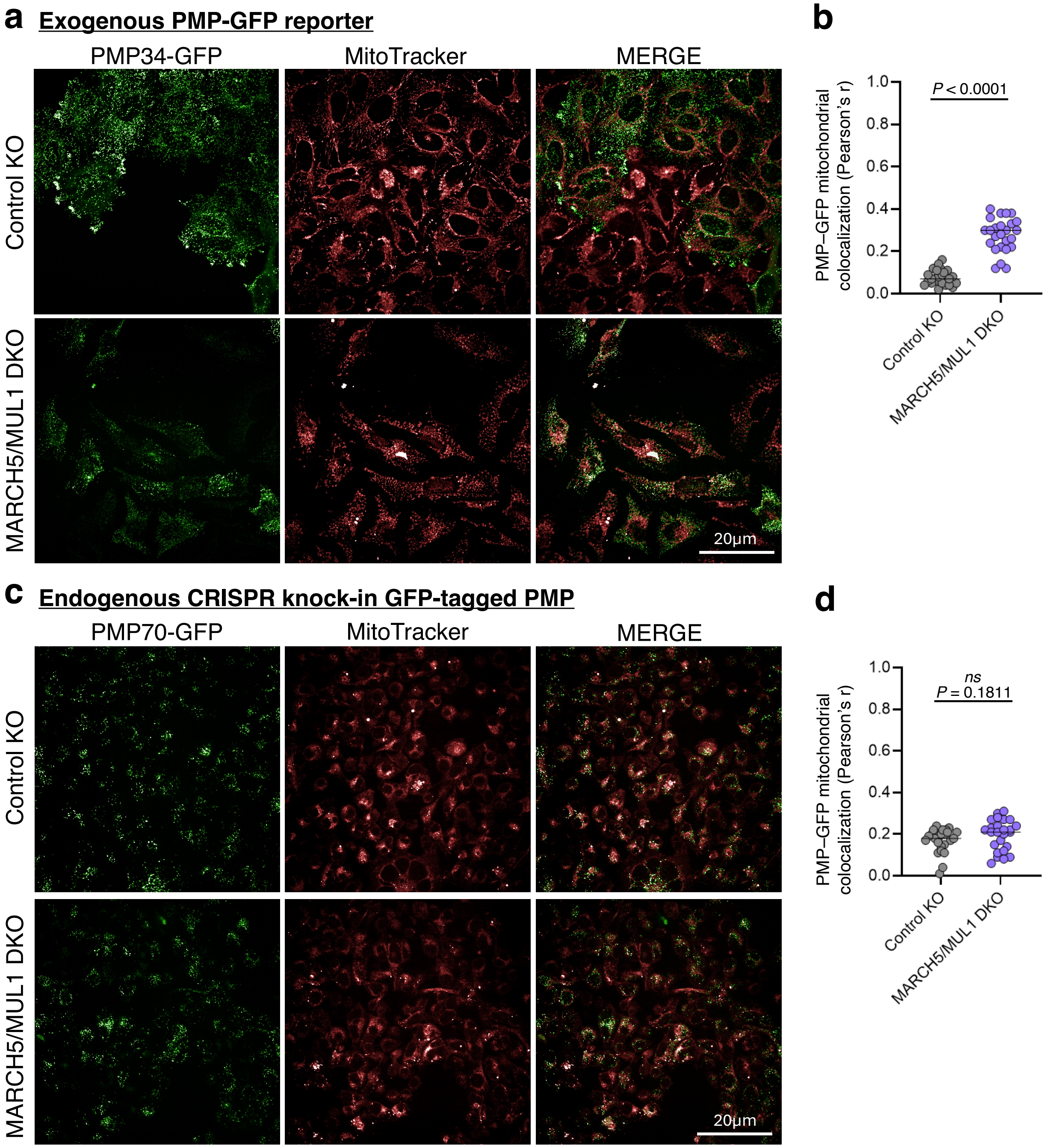

Supplementary Fig. 8. **In cells with intact peroxisomes, exogenous but not endogenous PMPs are detected in mitochondria of *MARCH5/MUL1*-deficient cells.**

(a) Representative confocal fluorescence microscopy images of the exogenous PMP34-GFP reporter expressed in the indicated genetic backgrounds of HeLa cells. Green, PMP-GFP; red, mitochondrial marker MitoTracker. MERGE panels show the overlay of PMP34-GFP and MitoTracker. Scale bar, 20 μm.

(b) Quantification of colocalization between exogenous PMP34-GFP and MitoTracker using Pearson's correlation coefficient (Pearson's r). Increased colocalization is observed in

MARCH5/MUL1-deficient cells (DKO). Data are presented as individual data points with mean values; $n = 25$ cells per genetic background. The P value was determined by a two-tailed unpaired t-test and is indicated in the figure.

(c) Representative confocal fluorescence images of endogenous PMP70-GFP in the indicated genetic backgrounds of HEK293T CRISPR knock-in cells. Green, PMP70-GFP; red, MitoTracker. MERGE panels show the overlay of PMP70-GFP and MitoTracker. Scale bar, 20 μm.

(d) Quantification of colocalization between endogenous PMP70-GFP and MitoTracker in control KO or *MARCH5/MUL1* DKO cells using Pearson's r. Data are presented as individual data points with mean values; $n = 25$ cells per genetic background. The P value was determined by a two-tailed unpaired t-test and is indicated in the figure. ns, not significant.
