# Supplementary material for: A mitochondria-driven quality control mechanism for peroxisomal membrane proteins
Source: Nat Commun. 2026 Jun 10;17:7375. doi: 10.1038/s41467-026-74117-6 (PMC13402316; doi:10.1038/s41467-026-74117-6)
Supplement: Supplementary file 2 — Description of Additional Supplementary Files [file 41467_2026_74117_MOESM2_ESM.pdf]

Description of Additional Supplementary Files

File Name: Supplementary Data 1.

Description: Label-free mass spectrometry-based proteomic analysis of *PEX3* KO versus control KO HEK293T cell lines.

File Name: Supplementary Data 2.

Description: RNA-seq analysis comparing the transcriptome of *PEX3* KO cells to control KO HEK293T cells.

File Name: Supplementary Data 3.

Description: Results from CRISPR screens on PMP-GFP reporters analyzed using MAGeCK. (a) PMP24-GFP, (b) PEX11A-GFP, (c) PEX11B-GFP.
