# Supplementary material for: A mitochondria-driven quality control mechanism for peroxisomal membrane proteins
Source: Nat Commun. 2026 Jun 10;17:7375. doi: 10.1038/s41467-026-74117-6 (PMC13402316; doi:10.1038/s41467-026-74117-6)
Supplement: Supplementary file 6 — Reporting Summary [file 41467_2026_74117_MOESM6_ESM.pdf]

nature portfolio

Corresponding author(s): Itay Koren

Last updated by author(s): May 13, 2026

Reporting Summary

Nature Portfolio wishes to improve the reproducibility of the work that we publish. This form provides structure for consistency and transparency in reporting. For further information on Nature Portfolio policies, see our Editorial Policies and the Editorial Policy Checklist.

Statistics

For all statistical analyses, confirm that the following items are present in the figure legend, table legend, main text, or Methods section.

n/a	Confirmed	
[ ]	[x]	The exact sample size (*n*) for each experimental group/condition, given as a discrete number and unit of measurement
[ ]	[x]	A statement on whether measurements were taken from distinct samples or whether the same sample was measured repeatedly
[ ]	[x]	The statistical test(s) used AND whether they are one- or two-sided<br>*Only common tests should be described solely by name; describe more complex techniques in the Methods section.*
[x]	[ ]	A description of all covariates tested
[x]	[ ]	A description of any assumptions or corrections, such as tests of normality and adjustment for multiple comparisons
[ ]	[x]	A full description of the statistical parameters including central tendency (e.g. means) or other basic estimates (e.g. regression coefficient) AND variation (e.g. standard deviation) or associated estimates of uncertainty (e.g. confidence intervals)
[ ]	[x]	For null hypothesis testing, the test statistic (e.g. *F*, *t*, *r*) with confidence intervals, effect sizes, degrees of freedom and *P* value noted<br>*Give P values as exact values whenever suitable.*
[x]	[ ]	For Bayesian analysis, information on the choice of priors and Markov chain Monte Carlo settings
[x]	[ ]	For hierarchical and complex designs, identification of the appropriate level for tests and full reporting of outcomes
[ ]	[x]	Estimates of effect sizes (e.g. Cohen's *d*, Pearson's *r*), indicating how they were calculated

Our web collection on statistics for biologists contains articles on many of the points above.

Software and code

Policy information about availability of computer code

Data collection

Western blot data were imaged by ImageQuant LAS 4000.
Flow cytometry data were collected using CytoFlex S (Beckman Coulter).
Cell proliferation was monitored using the IncuCyte® S3 Live-Cell Analysis System (Sartorius).
Microscopy images were acquired using an Opera Phenix Plus High-Content Screening System (PerkinElmer) in confocal mode.
RNAseq libraries were sequenced on an Illumina NextSeq 2000 platform in a single-end sequencing format.
MS was done by LC-MS/MS using an Exploris 480 mass spectrometer (Thermo) fitted with a capillary HPLC (Vanquish, Thermo scientific).

Data analysis

RNA-Seq analysis: RNA-Seq data quality was assessed using FastQC (v0.12.1). Sequencing reads were aligned to the human reference genome using STAR aligner (v2.7.10b) with default parameters. Gene-level quantification of aligned reads was performed using featureCounts (version 2.1.1) 59, and the number of successfully assigned reads per gene was recorded. Normalization of read counts and differential expression analysis between WT and KO samples were conducted using the DESeq2 60 R package (version 1.46.0), applying its standard workflow. P-values were adjusted for multiple testing using the Benjamini-Hochberg method to control the false discovery rate (FDR). Principal component analysis (PCA) was performed on variance-stabilized transformed expression data using DESeq2 to visualize sample clustering and variance between conditions.
The mass spectrometry data was analyzed using the DIA-NN software version 1.9.2
Flow cytometry data was analyzed with FlowJo v.10.9 (BD).
Pearson's correlation coefficient analysis was analyzed by the "Coloc2" plugin of ImageJ software and statistical analysis was performed using GraphPad Prism 10 software.
Peroxisome quantification was done via image analysis using Harmony high-content analysis software (Revvity v5.3).
Cell confluence was quantified automatically by the IncuCyte software (v2023B).

For manuscripts utilizing custom algorithms or software that are central to the research but not yet described in published literature, software must be made available to editors and reviewers. We strongly encourage code deposition in a community repository (e.g. GitHub). See the Nature Portfolio guidelines for submitting code & software for further information.

Data

Policy information about availability of data

All manuscripts must include a data availability statement. This statement should provide the following information, where applicable:

- Accession codes, unique identifiers, or web links for publicly available datasets
- A description of any restrictions on data availability
- For clinical datasets or third party data, please ensure that the statement adheres to our policy

Proteomics: The MS proteomic data have been deposited to ProteomeXchange via the PRIDE database under the identifier PXD069489.

RNA-seq: The data have been deposited to the NCBI GEO with accession number GSE310531

Research involving human participants, their data, or biological material

Policy information about studies with human participants or human data. See also policy information about sex, gender (identity/presentation), and sexual orientation and race, ethnicity and racism.

Reporting on sex and gender	N/A
Reporting on race, ethnicity, or other socially relevant groupings	N/A
Population characteristics	N/A
Recruitment	N/A
Ethics oversight	N/A

Note that full information on the approval of the study protocol must also be provided in the manuscript.

Field-specific reporting

Please select the one below that is the best fit for your research. If you are not sure, read the appropriate sections before making your selection.

☒ Life sciences ☐ Behavioural & social sciences ☐ Ecological, evolutionary & environmental sciences

For a reference copy of the document with all sections, see nature.com/documents/nr-reporting-summary-flat.pdf

Life sciences study design

All studies must disclose on these points even when the disclosure is negative.

Sample size	All experiments have been performed in at least three replicates.<br>Proteomics and transcriptomics were done in triplicates to acquire statistically robust quantification results.<br>For western blot and immunofluorescence experiments, sample sizes were determined based on prior research and widely accepted criteria in the scientific community.
Data exclusions	No Data exclusions.
Replication	Experiments were repeated independently at least three times. All replication attempts were successful.
Randomization	In cell-based experiments, including immunofluorescence, western blotting, RNAseq and mass spectrometry, randomization is neither

possible nor applicable, in accordance with generally accepted criteria in the scientific community.

Blinding	Western blot analysis was not blinded, as the gel loading order must be predefined. Blinding is not relevant or applicable to mass spectrometry, RNAseq or routine cell culture assays, as is generally accepted in the scientific community. Analyses were automated and performed without any bias.

Reporting for specific materials, systems and methods

We require information from authors about some types of materials, experimental systems and methods used in many studies. Here, indicate whether each material, system or method listed is relevant to your study. If you are not sure if a list item applies to your research, read the appropriate section before selecting a response.

Materials & experimental systems

n/a	Involved in the study	
☐	☒	Antibodies
☐	☒	Eukaryotic cell lines
☒	☐	Palaeontology and archaeology
☒	☐	Animals and other organisms
☒	☐	Clinical data
☒	☐	Dual use research of concern
☒	☐	Plants

Methods

n/a	Involved in the study	
☒	☐	ChIP-seq
☐	☒	Flow cytometry
☒	☐	MRI-based neuroimaging

Antibodies

Antibodies used	The following primary antibodies were used for western blotting:<br>mouse anti-vinculin (1:1,000; Sigma-Aldrich, #V9264), rabbit anti-HA tag (1:1,000; Cell Signaling Technology (CST), #3724), rabbit anti-GFP tag (1:1,000; Abcam, ab290), rabbit anti-PEX3 (1:1,000; ABclonal, #A7352), rabbit anti-PEX19 (1:1,000; proteintech, 14713-1-AP), rabbit anti-PEX14 (1:1,000; Bethyl, #A303-086A), rabbit anti-PEX13 (1:1,000; proteintech, #26649-1-AP), rabbit anti-PEX2 (1:1,000; StJohns (STJ) #117503), mouse anti-PMP70 (1:1,000; Sigma-Aldrich, #SAB4200181), rabbit anti-MARCH5 (1:1,000; CST #19168), rabbit anti-MUL1 (1:1,000; proteintech, #16133-1-AP), rabbit anti-HIF1a (1:1,000; Bethyl, #A300-286A), mouse anti-TOMM20 (1:1,000; santa cruz (SC) #17764), rabbit anti-BAG6 (1:1,000; CST #8523), rabbit anti-b-Tubulin (9F3) (1:1,000; CST #2128), rabbit anti-PEX26 (Proteintech #27472-1-AP), rabbit anti-PEX16 (Proteintech #14816-1-AP), rabbit anti- RNF187 (Novus Biologicals, #NBP1-91025), mouse anti-Catalase (R&D Systems, # MAB3398). HRP-conjugated secondary antibodies: goat anti-rabbit IgG (H+L) HRP (1:20,000, Jackson ImmunoResearch Labs, #111-035-144) or goat anti-mouse IgG (H+L) (1:20,000, Jackson ImmunoResearch Labs, #115-035-003).
Validation	All antibodies are commercial and were validated by the manufacturer for the purpose used in this study, i.e. immunoblotting and immunofluorescence using human cells.

Eukaryotic cell lines

Policy information about cell lines and Sex and Gender in Research

Cell line source(s)	HEK293T (ATCC® CRL-3216™); HeLa (A gift from Kimchi A. lab, Weizmann Institute of Science, source: CCL-185, respectively)
Authentication	Cell lines were authenticated by STR profiling.
Mycoplasma contamination	Cell lines were routinely tested for Mycoplasma contamination and were Mycoplasma-negative.
Commonly misidentified lines<br>(See ICLAC register)	None of the commonly misidentified lines was used in this study.

Plants

Seed stocks	N/A
Novel plant genotypes	N/A
Authentication	N/A

Flow Cytometry

Plots

Confirm that:

☒ The axis labels state the marker and fluorochrome used (e.g. CD4-FITC).

☒ The axis scales are clearly visible. Include numbers along axes only for bottom left plot of group (a 'group' is an analysis of identical markers).

☒ All plots are contour plots with outliers or pseudocolor plots.

☒ A numerical value for number of cells or percentage (with statistics) is provided.

Methodology

Sample preparation	Cells were detached with trypsin, washed once with PBS and then directly analyzed on the Flow cytometer.
Instrument	CytoFlex S (Beckman Coulter).
Software	FlowJo V.10
Cell population abundance	Live cells were ~80%, singlets were ~82%, DsRed positives were ~65%-70%
Gating strategy	Cells were first gated for live cells using forward and side scatter (FSC-A/SSC-A). Single cells were discriminated based on height vs. width of the forward scatter (FSC-A/FSC-H). Finally, GPS reporter positive cells were gated based on the DsRed expression.

☒ Tick this box to confirm that a figure exemplifying the gating strategy is provided in the Supplementary Information.
