# Supplementary material for: A mitochondria-driven quality control mechanism for peroxisomal membrane proteins
Source: Nat Commun. 2026 Jun 10;17:7375. doi: 10.1038/s41467-026-74117-6 (PMC13402316; doi:10.1038/s41467-026-74117-6)
Supplement: Supplementary file 7 — Transparent Peer Review file [file 41467_2026_74117_MOESM7_ESM.pdf]

nature portfolio

Peer Review File

A Mitochondria-Driven Quality Control Mechanism for Peroxisomal Membrane Proteins

Corresponding Author: Professor Itay Koren

This file contains all reviewer reports in order by version, followed by all author rebuttals in order by version.

Version 0:

Reviewer comments:

Reviewer #1

(Remarks to the Author)
In this manuscript, the authors demonstrated a new quality control pathway upon loss of peroxisome where part of peroxisomal membrane proteins (PMPs) are targeted and degraded at the outer mitochondrial membrane (OMM) via the OMM E3 ubiquitin ligases MARCH5 and MUL1. Specifically, the authors used PEX3, PEX19 KO cells that are lack of peroxisome due to impaired biogenesis. By transcriptomic and proteomic analysis, they found that many of the peroxisomal proteins are downregulated post-transcriptionally in peroxisome biogenesis deficient cells. Further, they used a reporter assay to identify several PMPs whose ubiquitylation is increased and stability is reduced. With CRISPR screen, they identified that the OMM anchored E3 ligases, MARCH5 and MUL1 are required redundantly for the ubiquitylation and degradation of certain PMPs. Moreover, they showed that these PMPs are redirected to OMM for degradation. Loss of MARCH5 and MUL1 further impairs cell proliferation in PEX3 KO cells. The identification of mitochondrial E3 ligases in peroxisomal protein degradation adds new insights into the cross-organellar protein quality control machinery. While in most cases data support conclusion, the authors need to provide solid data to confirm the increased ubiquitylation and reduced stability of endogenous PMPs. Here are specific comments:
1. Cycloheximide chase assay should be performed to validate the reduced stability of endogenous candidate PMPs at the OMM when peroxisome biogenesis is impaired. Using PEX14 as control would be helpful.
2. Fig. 2d: PEX3 KO and PEX19 KO cells have much less PEX11B and PMP34 expression according to fluorescence imaging (Fig. 2b and Fig. 4a). However, while the total cell Ub levels are similar between control and KO cells, GFP level did not show comparable difference as in fluorescence images. Is this an artifact of the overexpression system? Otherwise, to achieve same amount of GFP, one would need much more KO cells (therefore more total proteins and more intense Ub signal) than WT cells. This also applies to Fig. 3d. Could the authors comment on this and provide more experimental details?
3. Do MARCH5 and MUL1 get involved in PMP degradation under physiological condition? Does MARCH5/MUL1 DKO cells show enhanced mitochondrial localization of PMPs? Fig. 3c and Fig. S3b seem to suggest some PMPs (PMP34, PEX11A) being stabilized in MARCH5/MUL1 DKO cells, but this needs to be properly quantified. This is to have a better idea whether PMP targeting to mitochondria is a stress-/loss of peroxisome-driven process.
4. For PEX2 blots, is the second band an unspecific band or another form of PEX2? This should be validated by KO/KD experiment.
5. Western blot data should be quantified or stating if independent replicates showing consistent results.
6. Colocalization of PMPs with mitochondria should be quantified in Fig. S4.
7. Flow cytometry analysis such as in Fig. 2c and Fig. 3c should be quantified not only showing representative plots.

Reviewer #2

(Remarks to the Author)
In this study, Dr. Segev-Nakar and Dr. Koren report a novel mitochondria-driven quality control mechanism for peroxisomal membrane proteins (PMPs). They show that, in cells lacking a functional peroxisomal membrane biogenesis machinery, PMP expression is downregulated at the post-transcriptional level, and the proteins are mistargeted to mitochondria. The authors identified the mitochondrial E3 ligases MUL1 and MARCH5 as key factors that ubiquitinate these mistargeted PMPs, directing them for proteasomal degradation. This mechanism preserves mitochondrial proteostasis and supports cell viability. Overall, the data are solid and clearly presented, although some findings are less novel than occasionally

suggested. Addressing several remaining concerns and clarifying certain aspects would further enhance the quality and clarity of the work.

Specific remarks

Hypotheses and interpretation of the results

1. Introduction – The authors state “Based on this, we hypothesize that similar mechanisms may also act on mislocalized proteins from other organelles, such as peroxisomes, by recognizing their targeting signals.” However, the experiments presented do not address this hypothesis. The study does not demonstrate that any of the identified membrane protein targeting signals (mPTSs) of the proteins examined (e.g., PEX2, PEX3, PEX13, PEX14, or PMP70) contribute to their degradation. The study should be properly contextualized, or the authors must provide direct experimental evidence to support their claim.

2. Results – The statement that mitochondrial targeting of PMPs occurs independently of PEX19 in peroxisome-deficient cells is already established (e.g., DOI: 10.1083/jcb.148.5.931). The authors should cite these prior studies and frame their results as confirmation rather than a novel finding.

3. Discussion – The authors state that their results suggest that accumulation of mitochondria-localized PMPs may contribute to mitochondrial dysfunction upon peroxisome loss. However, this concept has already been demonstrated in prior studies (doi: 10.15252/embj.201487943; doi: 10.15252/embr.202051991; DOI: 10.15252/embr.202153790) and is therefore not novel. The authors should clarify this context and emphasize that the novelty of their work lies in identifying a new underlying mechanism.

Experimental design, controls, and reproducibility

4. Figure 1a – The authors analyzed two PEX3 knockout clones, PEX3 KO1 and PEX3 KO2. In the PCA plot, however, the clones are separated by a large distance along PC1, despite being expected to be functionally similar and therefore cluster together? Does this separation indicate major differences between the clones, potentially due to off-target effects? While the authors compare both clones in Figure 1 and Supplementary Figure 1, it is unclear which clone was used for the other experiments? Did they confirm the findings in the second clone reintroduce PEX3 to rule out off-target effects? This should be clarified in the manuscript. In addition, the term “control KO” is ambiguous. Do the authors mean the corresponding parental cell line? If so, it should be clearly annotated throughout the manuscript.

5. The authors state that all experiments were independently repeated at least 3 times with consistent outcomes. However, the supplementary RNA datasets include only 2 wild-type samples. It is also unclear whether the flow cytometry plots represent averaged data from 3 independent experiments or a single selected dataset. In addition, the authors should quantify the blot signals from all 3 biological replicates and provide appropriate statistical comparisons for the relevant conditions.

6. Mitochondrial purification – The authors use a commercial kit to isolate mitochondria. However, this method relies on differential centrifugation, it produces mitochondria-enriched fractions rather than highly purified mitochondria. This is evident from the presence of peroxisomal markers in the mitochondrial fraction from control cells (Fig. 4c and S4d), indicating contamination by other organelles. In addition, the term “cytoplasm” is inaccurate here, as it typically refers to the cellular content enclosed by the cell membrane but outside the nucleus, whereas the fraction labeled as such corresponds to the 12,000 x g supernatant. Finally, the term “mitochondrial lysis buffer” is misleading, as the purpose of this buffer is to lyse cells while preserving mitochondrial integrity rather than lysing mitochondria themselves.

7. The authors identify MUL1 and MARCH 5 as E3 ligases ubiquitinating mislocalized PMPs in their CRISPR (Fig. 3b), yet later state that these ligases function redundantly in PMP degradation (Supplementary Fig. 3b). It is difficult to reconcile this conclusion with the screening conditions, as the sgRNA library DNA was delivered at an MOI of ~0.3. Under these conditions, and even with 1000 x library representation – the probability that any given cells receives sgRNAs targeting both MUL1 and MARCH5 is extremely low. If single gene knockouts produced minimal phenotypes, it therefore remains unclear how the screen could have detected this redundant pair. The authors should clarify how their screen design supports these findings or provide independent evidence validating the proposed redundancy.

8. The authors propose that MARCH5 and MUL1 continuously manage a small pool of mislocalized PMPs to maintain proteostasis and cell viability. It is unclear whether the effect is also observed in control cells (e.g., with or without Bortezomib or MLN7243 treatment) or in cells lacking both E3 ligases but with intact peroxisome biogenesis). The authors should clarify whether these conditions were tested and how they support the proposed model.

Data presentation

9. The Y-axes in the flow cytometry plots (Figs. 2c, 3c, S2b, S2D, and 3) need proper labeling. In addition, the authors should indicate how many cells were analyzed in each experiment.

10. The authors report that 149 were downregulated and 63 upregulated in PEX3-deficient cells relative WT (Welch’s t-test, $p < 0.05$; $\log_{10}$ fold change <-1 or >+1 for upregulated proteins) However, Supplementary Table 1 and Figures 1b,c present log2 fold-change values, not log10. This discrepancy should be corrected for consistency and accuracy.

Nomenclature and accuracy

11. The authors state that catalase contains a peroxisomal targeting signal type 1 (PTS1), composed of the tripeptide serine-lysine-leucine (-SKL), is incorrect. Catalase carries a non-canonical PTS1 sequence (-KANL; DOI: 10.1083/jcb.134.4.849). Unless the authors have engineered this sequence – something not indicated in the Materials and Methods – the statement is inaccurate and should be corrected.

12. Protein descriptions in text and tables – Several proteins cannot be easily traced in the supplementary tables because the text uses synonyms while the tables use official nomenclature (e.g., ABCD3 is referred to as PMP70 in the text). The authors should standardize protein names across the manuscript and supplementary materials to ensure consistency and clarity for non-experts.

Reviewer #3

(Remarks to the Author)
The study by Segev-Nakar and Koren demonstrated the widespread downregulation of peroxisomal membrane proteins (PMPs) in cells lacking the PEX3 or PEX19 genes. These genes play a pivotal role in the targeting and import of PMPs to peroxisomes and are therefore essential for peroxisome biogenesis. The authors showed that the downregulation of PMPs in cells deficient in peroxisome biogenesis (PEX3 or PEX19 knockout (KO) cells) is due to decreased protein stability rather than reduced mRNA levels. They subsequently demonstrated that PMPs that fail to reach peroxisomes in PEX3 KO cells are instead directed to the mitochondria. There, the E3 ligases MUL1 and MARCH5 ubiquitinate the PMPs, promoting their degradation by the proteasome and safeguarding cellular proteostasis. Furthermore, the authors demonstrated that cells with triple KOs of PEX3/MUL1/MARCH5 exhibited greater impairment in cell proliferation than PEX3 KO or MUL1/MARCH5 KO cells. Unlike the degradation of mislocalised ER proteins by the cytosolic protein quality control (PQC), the authors proposed an additional layer of PQC involving mitochondria-residing E3 ligases in cells with deficient peroxisome biogenesis.

While the postulation of tight cross-talk between the peroxisome and mitochondria in protein quality control is interesting, unfortunately the data presented here do not fully support the authors' conclusion. In particular, their conclusion that PMPS are rerouted to mitochondria in PEX3 KO cells cannot be fully supported by their immunofluorescence and fractionation data. Secondly, it is unclear whether this is a general phenomenon as the PMPs degraded by MARCH5/MUL1 seemed limited. The specific points have been outlined below.

Fig. 1: Add the positions of PMP70, PEX13 and PEX14 (Fig. 1e) to the diagram in Fig. 1b. In Fig. 1e, it would be more convincing if more PMPs were shown in immunoblotting in HEK293T cells, with quantification (mean, SE).

Fig. 2b: Include staining with a peroxisome-specific dye or marker, as well as co-staining with PEX11B-GFP in PEX3 and PEX19 KO cells. I strongly recommend observing PEX11B-GFP in the presence of bortezomib in PEX3 or PEX19 knockout (KO) cells. What is the expected subcellular localisation of PEX11B in KO cells treated with bortezomib?

Figure 3d: The authors would demonstrate an in vivo ubiquitination assay for endogenous PMP34 or other PMPs. In Fig. 3e, changes in PEX2 levels were minimal. The authors could replace this or quantify the change.

Fig. 4a. Again, the authors should verify the absence of peroxisome (complete loss?) in PEX3 KO cells using other peroxisome-specific markers. Mitochondria are scattered throughout the cytoplasm in control KO cells, whereas perinuclear aggregation of mitochondria was observed in PEX3 and TKO cells. This perinuclear aggregation of mitochondria can occur dependent on organelle-specific motor proteins and microtubules. Is it possible that the remaining peroxisomes or PMPs in TKO cells move to the perinuclear region in these stressed conditions?
Given the method of mitochondrial purification used, it is highly likely that the so-called 'mitochondria fraction' contains both mitochondria and peroxisomes. Indeed, the mitochondrial fraction of the control KO cells in Fig. 4c contained abundant PMP70, PEX13 and PEX14 along with TOM20. Therefore, the authors cannot conclude that PMPs are redirected to mitochondria without conducting further experiments to verify the complete loss of peroxisomes in TKO cells. The authors should also include a co-IP experiment showing the interaction between MARCH5/MUL1 and PMP34 as well as some of other PMPs.

Minor
SFig.1c. In PEX3 and PEX19 KO cells, GFP-CAT lost its punctate fluorescence pattern and displayed both diffused cytoplasmic and nuclear fluorescence.

The left parentheses were missing in the indication of Supplementary figures throughout the text.

Version 1:

Reviewer comments:

Reviewer #1

(Remarks to the Author)
The authors addressed all of my concerns convincingly. The manuscript is suitable for publication in Nature Communications.

Reviewer #2

(Remarks to the Author)
The authors have adequately addressed all my concerns, and the manuscript has been significantly strengthened as a result. I greatly appreciate the authors' careful and thorough revision.

Reviewer #3

(Remarks to the Author)
I appreciate the authors' endeavor to address the comments by this reviewer. However, it is still missing the important biochemical evidence of interaction and ubiquitylation between PMPs and MARCH5/MUL1. With exogenously expressing GFP- or Myc-tagged MARCH5 with HA-ubiquitin (Yanagi S et al.,), the authors would be able to address these concerns.

Open Access This Peer Review File is licensed under a Creative Commons Attribution 4.0 International License, which permits use, sharing, adaptation, distribution and reproduction in any medium or format, as long as you give appropriate credit to the original author(s) and the source, provide a link to the Creative Commons license, and indicate if changes were made.
In cases where reviewers are anonymous, credit should be given to 'Anonymous Referee' and the source.
The images or other third party material in this Peer Review File are included in the article's Creative Commons license, unless indicated otherwise in a credit line to the material. If material is not included in the article's Creative Commons license and your intended use is not permitted by statutory regulation or exceeds the permitted use, you will need to obtain permission directly from the copyright holder.
To view a copy of this license, visit https://creativecommons.org/licenses/by/4.0/

We sincerely appreciate the reviewers' thoughtful feedback and constructive suggestions for additional experiments and improved clarity. In response, we have addressed all comments and editorial requests through new experiments, rigorous quantification, and careful revisions to the text and figures. Changes from the original submission are highlighted in red in the revised manuscript.

Major additions and improvements to the manuscript:

1. Elucidation of PMP recognition motif: To determine the signal that targets PMPs for degradation, we generated a minimal reporter fusing the transmembrane domain (TMD) of PEX26 to GFP. We demonstrate that the TMD is sufficient to drive mitochondrial mislocalization and subsequent MARCH5/MUL1-dependent degradation, providing a mechanistic basis for this quality control pathway.
2. Expanded PMP repertoire subjected to degradation upon peroxisomes loss: We validated additional antibodies for endogenous PMPs (PEX16 and PEX26) and confirmed by immunoblotting that they are also downregulated in peroxisome-deficient cells. Notably, all proteins tested but one were regulated by MARCH5/MUL1, highlighting the broad scope of this quality control pathway.
3. Analysis of endogenous PMP stability: We performed cycloheximide chase assays to measure the turnover of endogenous PMPs. These experiments confirm that endogenous PEX13 destabilization in PEX3 KO cells is strictly dependent on MARCH5 and MUL1, mirroring our findings with exogenous reporters.
4. Confirmation of peroxisome loss and mitochondrial purity: We employed the novel peroxisome-specific probe PeroxiSPY650 and immunoblotting for the peroxisomal matrix protein Catalase to demonstrate the complete absence of peroxisomes in PEX3 KO and MARCH5/MUL1/PEX3 TKO cells. This crucially confirms that PMP signals detected in mitochondria-enriched fractions of these cells represent genuine mitochondrial rerouting rather than peroxisomal contamination. Consistent with these biochemical data, quantitative confocal microscopy analyses in both HeLa and HEK293T cells demonstrate significant co-localization of PMPs with mitochondrial markers specifically in peroxisome-deficient cells.
5. Analysis of PMP targeting fidelity under physiological conditions: Using CRISPR knock-in cell lines expressing endogenous GFP-tagged PMP70 and PEX11B, we demonstrated that under physiological conditions (unlike upon exogenous overexpression or disruption of peroxisome biogenesis), endogenous PMPs are efficiently targeted to peroxisomes, with mitochondrial mislocalization remaining below the detection limit.
6. Quantification and statistical analysis: We have quantified all Western blots, immunofluorescence microscopy, and flow cytometry data from at least three independent biological replicates. Representative images are now accompanied by statistical analyses in the figures.

REVIEWER COMMENTS

Reviewer #1 (Remarks to the Author):

In this manuscript, the authors demonstrated a new quality control pathway upon loss of peroxisome where part of peroxisomal membrane proteins (PMPs) are targeted and degraded at the outer mitochondrial membrane (OMM) via the OMM E3 ubiquitin ligases MARCH5 and MUL1. Specifically, the authors used PEX3, PEX19 KO cells that are lack of peroxisome due to impaired biogenesis. By transcriptomic and proteomic analysis, they found that many of the peroxisomal proteins are downregulated post-transcriptionally in peroxisome biogenesis deficient cells. Further, they used a reporter assay to identify several PMPs whose ubiquitylation is increased and stability is reduced. With CRISPR screen, they identified that the OMM anchored E3 ligases, MARCH5 and MUL1 are required redundantly for the ubiquitylation and degradation of certain PMPs. Moreover, they showed that these PMPs are redirected to OMM for degradation. Loss of MARCH5 and MUL1 further impairs cell proliferation in PEX3 KO cells. The identification of mitochondrial E3 ligases in peroxisomal protein degradation adds new insights into the cross-organellar protein quality control machinery. While in most cases data support conclusion, the authors need to provide solid data to confirm the increased ubiquitylation and reduced stability of endogenous PMPs.

We thank the reviewer for acknowledging that "The identification of mitochondrial E3 ligases in peroxisomal protein degradation adds new insights into the cross-organellar protein quality control machinery", and for their constructive comments that helped improve the manuscript.

Here are specific comments:

1. Cycloheximide chase assay should be performed to validate the reduced stability of endogenous candidate PMPs at the OMM when peroxisome biogenesis is impaired. Using PEX14 as control would be helpful.

We thank the reviewer for this helpful suggestion. We have performed a cycloheximide (CHX) chase assay to assess the stability of endogenous PMPs. In PEX3 KO cells, the PMPs PEX2, PMP70, and PEX26 are below the detection limit by both mass spectrometry (Supplementary Data 1) and immunoblotting (Fig. 1d), which precludes direct comparison with MARCH5/MUL1/PEX3 TKO cells. Therefore, we focused on PEX13, which shows partial reduction in levels. As suggested, PEX14 was included as a negative control, and the short-lived protein RNF187 (PMID: 34626566) served as a positive control.

Consistent with the GPS data indicating reduced stability of exogenous proteins in PEX3 KO cells, CHX assay revealed a decrease in endogenous PEX13 levels in PEX3 KO cells **(Supplementary Fig. 4c,d)**. Notably, comparison of the two peroxisome-deficient conditions, PEX3 KO and MARCH5/MUL1/PEX3 TKO cells, demonstrated that mitochondrial turnover of PEX13 was completely blocked in the absence of MUL1 and MARCH5 (**Supplementary Fig. 4c,d**), indicating that PEX13 turnover at the outer mitochondrial membrane depends on these E3 ligases.

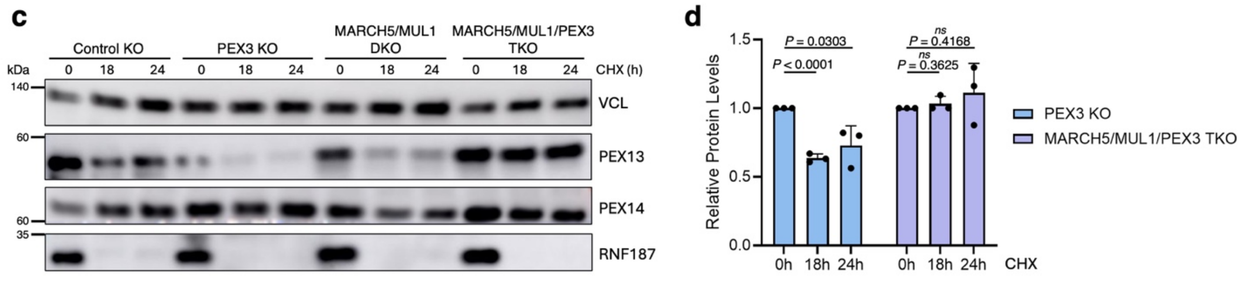

Supplementary Fig. 4c,d. Cycloheximide (CHX) chase assay in the indicated KO cells treated with 50 µg/ml CHX for the specified time points, followed by immunoblot analysis of PEX13 and PEX14 protein levels. VCL serves as a loading control and RNF187 serves as a short-lived positive control to confirm CHX treatment efficacy. A representative immunoblot is shown in (c). Quantitative analysis of relative PEX13 protein levels, normalized to VCL and the 0 h time point is shown in (d). Data represent mean ± s.d. from n = 3 independent experiments. Significance was determined using a two-tailed unpaired t-test comparing the indicated time points to 0 h for each genetic background. ns, not significant.

2. Fig. 2d: PEX3 KO and PEX19 KO cells have much less PEX11B and PMP34 expression according to fluorescence imaging (Fig. 2b and Fig. 4a). However, while the total cell Ub levels are similar between control and KO cells, GFP level did not show comparable difference as in fluorescence images. Is this an artifact of the overexpression system? Otherwise, to achieve same amount of GFP, one would need much more KO cells (therefore more total proteins and more intense Ub signal) than WT cells. This also applies to Fig. 3d. Could the authors comment on this and provide more experimental details?

Thank you for raising this point. The *in vivo* ubiquitination assays were performed under conditions of proteasome inhibition using bortezomib, which blocks degradation of ubiquitinated proteins. As a result, ubiquitinated GFP-tagged PMPs accumulate, leading to similar total levels of GFP-fused PMP in both WT and KO cells at the time of immunoprecipitation with GFP beads. This explains why the GFP signal in the ubiquitination assays does not mirror the differences observed in steady-state fluorescence imaging.

Although this information was included in the Methods section, we have now clarified it in the main text and in the figure legends to avoid confusion. Main text was edited as follows:

Page 9: "To test this, GFP-fused PMPs were immunoprecipitated (IP) from cells co-expressing HA-tagged ubiquitin. Prior to IP, cells were treated with the proteasome inhibitor bortezomib to prevent degradation of the substrate and allow accumulation of ubiquitinated proteins".

3. Do MARCH5 and MUL1 get involved in PMP degradation under physiological condition? Does MARCH5/MUL1 DKO cells show enhanced mitochondrial localization of PMPs? Fig. 3c and Fig. S3b seem to suggest some PMPs (PMP34, PEX11A) being stabilized in MARCH5/MUL1 DKO cells, but this needs to be properly quantified. This is to have a better idea whether PMP targeting to mitochondria is a stress-/loss of peroxisome-driven process.

This point was also raised by Reviewer 2 (comment #8), and we have now addressed it quantitatively.

Fluorescent microscopy analysis shows significantly increased co-localization of the PMP34 reporter with mitochondria in MARCH5/MUL1 DKO cells compared to control cells **(Supplementary Fig. 8a,b)**. As expected, this colocalization was less pronounced than that observed in cells deficient in peroxisomes (Supplementary Fig. 6a-c). These results suggest that upon exogenous expression, a fraction of PMPs is mistargeted to mitochondria even in cells with intact peroxisomes and is subsequently degraded by MARCH5/MUL1. This underscores the importance of this pathway in the surveillance of mislocalized PMP species. Our findings are consistent with previous reports showing that overexpression of PEX14 results in mitochondrial mistargeting (PMID: 38762172).

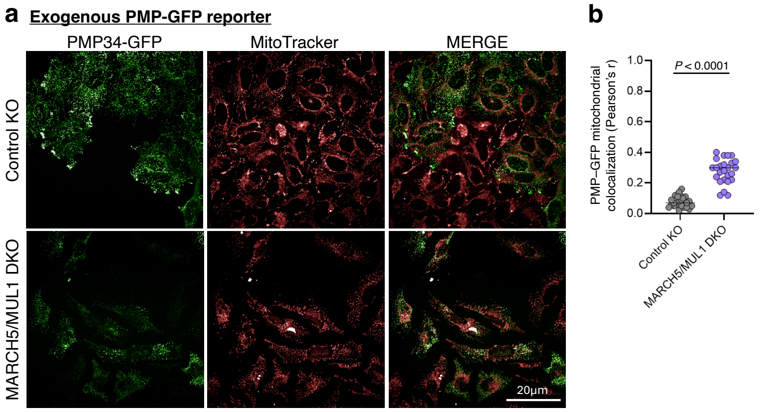

Supplementary Fig. 8. (a) Representative confocal fluorescence microscopy images of the exogenous PMP34-GFP reporter expressed in the indicated genetic backgrounds. Green, PMP-GFP signal; red, mitochondrial marker MitoTracker. Scale bar, 20 μm. (b) Quantification of colocalization between exogenous PMP34-GFP and MitoTracker using Pearson's correlation coefficient (Pearson's r). $n \geq 25$ cells per condition. Data are presented as mean ± s.d. P values were determined by two-tailed unpaired t-test.

Consistent with these results, we performed quantitative analysis of the flow cytometry data. In cells expressing exogenous GPS-PMP reporters, PMP stability is reduced under conditions of peroxisome loss and is restored upon MARCH5/MUL1 double knockout (DKO). In cells lacking MARCH5 and MUL1 but retaining intact peroxisomes, we observe an increase in PMP stability; however, the degree of stabilization is lower than that observed in peroxisome-deficient cells **(Fig. 3c,d).**

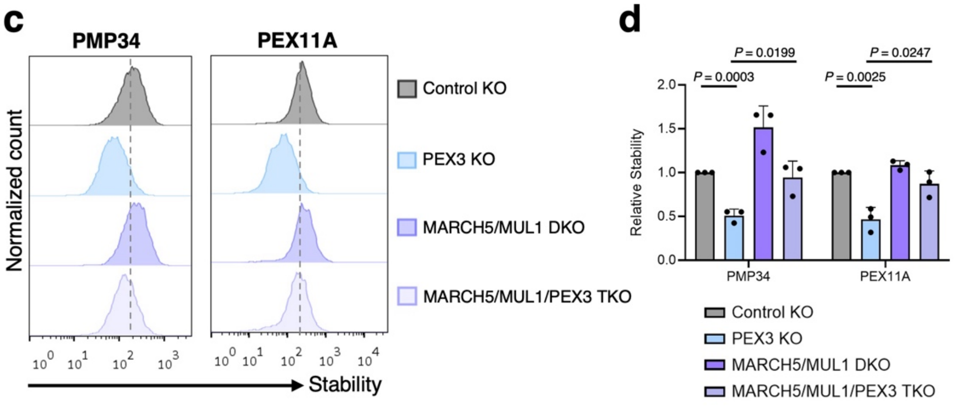

Fig. 3c,d. Stability analysis of PMP34 and PEX11A GPS reporters in control cells, MUL1/MARCH5 double KO (DKO) cells, PEX3 KO cells, and PEX3 KO cells additionally lacking both MUL1 and MARCH5 (MARCH5/MUL1/PEX3 TKO). Representative flow cytomeetry histograms (c) and quantitative analyses (mean GFP/DsRed fluorescence normalized to control KO ± s.d.) from n = 3 independent experiments (d) are shown. *P* values were determined using a two-tailed unpaired t-test.

Based on these observations, we hypothesize that, similar to other protein targeting pathways, peroxisomal protein targeting is not completely error-free. A small fraction of PMPs might mislocalize to mitochondria, a phenomenon that becomes more pronounced upon overexpression, as previously reported (PMID: 38762172). This mitochondrial pool of mislocalized PMPs is then recognized and degraded by MARCH5 and MUL1.

To determine whether this phenomenon also occurs under physiological conditions, we examined the localization of endogenous PMPs using a CRISPR knock-in cell line in which GFP was fused to PMP70 (PMID: 40689797). Importantly, in contrast to the exogenous expression setting, no significant increase in mitochondrial localization of these endogenous PMPs was observed in MARCH5/MUL1 DKO cells **(Supplementary Fig. 8c,d)**.

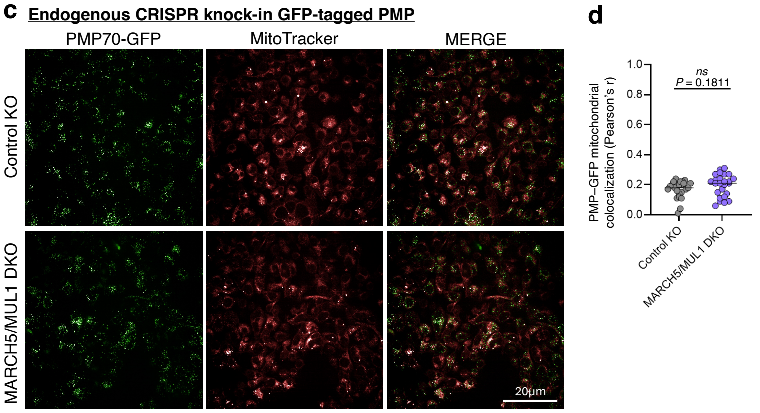

Supplementary Fig. 8. (c) Representative confocal fluorescence images of endogenous PMP70-GFP in the indicated genetic backgrounds of HEK293T CRISPR knock-in cells. Green, PMP70-GFP; red, MitoTracker. Scale bar, 20 µm. (d) Quantification of colocalization between endogenous PMP70-GFP and MitoTracker in control KO or MARCH5/MUL1 DKO cells using Pearson's correlation coefficient (Pearson's r). n ≥ 25 cells per condition. Data are presented as mean ± s.d. P values were determined by two-tailed unpaired t-test. ns, not significant.

In agreement with this, cellular fractionation experiments did not reveal increased steady-state levels of endogenous PMPs in the mitochondria-enriched fraction of MARCH5/MUL1 DKO cells compared to control KO cells **(Fig. 4c)** (see control KO [black bars] with MARCH5/MUL1 DKO [purple bars]).

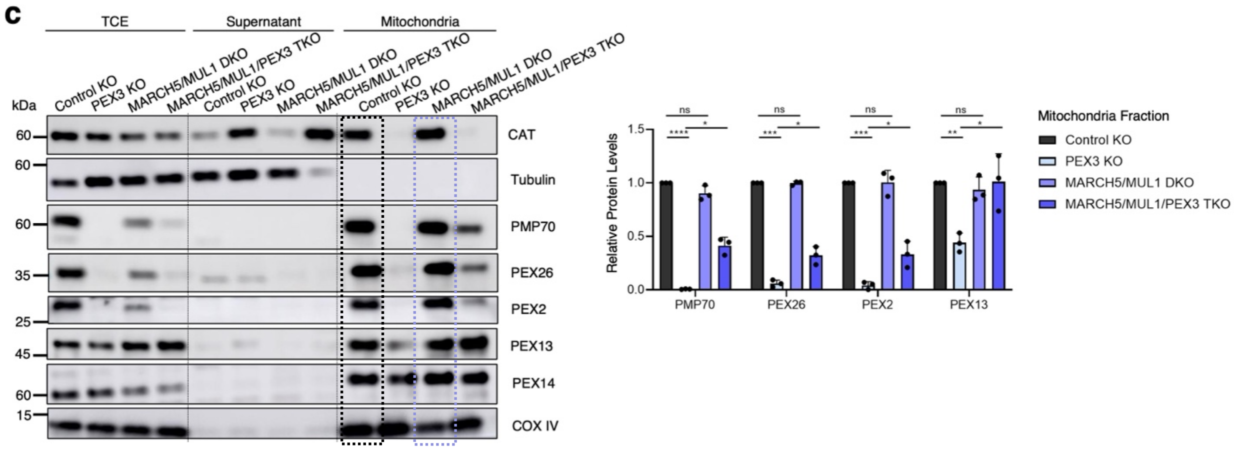

Fig. 4c. Cellular fractionation of indicated KO cells followed by immunoblot of endogenous PMPs in the mitochondria-enriched fraction ("Mitochondria") and the mitochondria-depleted supernatant ("Supernatant"). COX IV and Tubulin serve as mitochondrial or supernatant protein loading controls, respectively. A representative immunoblot is shown in (c). Quantitative analyses (mean ± s.d.) from n = 3 independent experiments is shown on the right. P values were determined using a two-tailed Student's t-test. *P < 0.01, **P < 0.001, ***P < 0.0001. ns- non-significant.

Together, these results indicate that while mistargeting of PMPs to mitochondria can occur upon overexpression and is subject to MARCH5/MUL1-dependent degradation, endogenous PMPs are efficiently and faithfully targeted to peroxisomes under physiological conditions. We cannot exclude the possibility that a very small fraction of endogenous PMPs is mistargeted; however, if present, such events are below the detection limit of our microscopy and biochemical approaches.

4. For PEX2 blots, is the second band an unspecific band or another form of PEX2? This should be validated by KO/KD experiment.

The upper band corresponds to a non-specific signal, as confirmed by a PEX2 knockout experiment. These data have now been added as **Supplementary Fig. 1f.**

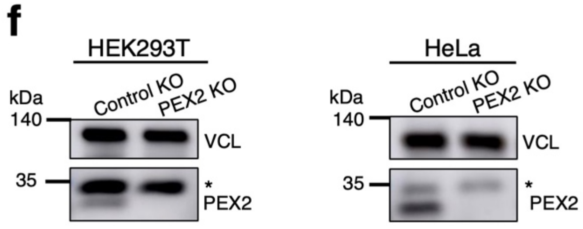

Supplementary Fig. 1f. Immunoblot analysis of PEX2 in control or PEX2 knockout (KO) HEK293T cells (left) and HeLa cells (right) using an anti-PEX2 antibody. Vinculin (VCL) was used as a loading control. The absence of the lower band in KO cells indicates that this band corresponds to PEX2, whereas the upper band persisted and is therefore considered non-specific.

5. Western blot data should be quantified or stating if independent replicates showing consistent results.

We thank the reviewer for this suggestion. The Western blot data have now been quantified, and the corresponding graphs have been added to the revised manuscript. Additionally, the figure legend now indicates that the results are representative of 3 independent replicates, all showing consistent trends.

6. Colocalization of PMPs with mitochondria should be quantified in Fig. S4.

Colocalization of PMPs with mitochondria in Fig. S4 (new **Supplementary Fig. 6**) has now been quantified, and the corresponding data have been added to the figure and its legend.

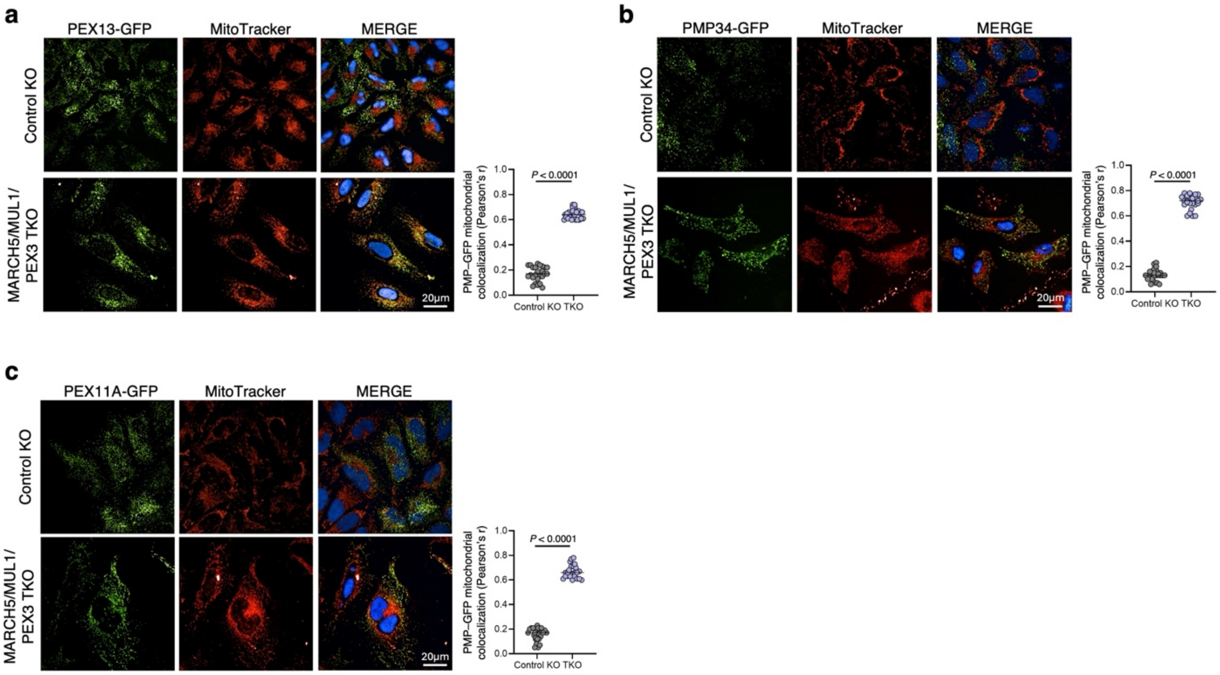

Supplementary Fig. 6. Peroxisome loss redirects PMPs to mitochondria for MARCH5/MUL1-dependent degradation. (a-c) Representative confocal fluorescence microscopy images of PEX13-GFP (a), PMP34-GFP (b), and PEX11A-GFP (c) reporters in the indicated genetic backgrounds of HeLa cells. Green, PMP-GFP signal; red, mitochondrial marker MitoTracker. MERGE panels show the overlay of PMP-GFP, MitoTracker, and Hoechst nuclear stain (blue). Scale bar, 20 µm. Pearson's correlation coefficient (Pearson's r) analysis for the colocalization of PMP-GFP from (a-c) with MitoTracker is shown in the graphs adjacent to the images, demonstrating increased colocalization in cells deficient for both PEX3 and MARCH5/MUL1 (TKO). Data are presented as individual data points with mean values; $n \geq 25$ cells were analyzed per sample. P values were determined by a two-tailed unpaired t-test.

7. Flow cytometry analysis such as in Fig. 2c and Fig. 3c should be quantified not only showing representative plots.

The flow cytometry data in Fig. 2c and Fig. 3c have now been quantified by calculating the mean fluorescence under the different conditions and genetic backgrounds. The quantified data have been added to the figures and figure legends, in addition to the representative plots.

Reviewer #2 (Remarks to the Author):

In this study, Dr. Segev-Nakar and Dr. Koren report a novel mitochondria-driven quality control mechanism for peroxisomal membrane proteins (PMPs). They show that, in cells lacking a functional peroxisomal membrane biogenesis machinery, PMP expression is downregulated at the post-transcriptional level, and the proteins are mistargeted to mitochondria. The authors identified the mitochondrial E3 ligases MUL1 and MARCH5 as key factors that ubiquitinate these

mistargeted PMPs, directing them for proteasomal degradation. This mechanism preserves mitochondrial proteostasis and supports cell viability. Overall, the data are solid and clearly presented, although some findings are less novel than occasionally suggested. Addressing several remaining concerns and clarifying certain aspects would further enhance the quality and clarity of the work.

We thank the reviewer for their kind comment that "Overall, the data are solid and clearly presented" and for their suggestions to further enhance the clarity and quality of the work.

Hypotheses and interpretation of the results

1. Introduction – The authors state "Based on this, we hypothesize that similar mechanisms may also act on mislocalized proteins from other organelles, such as peroxisomes, by recognizing their targeting signals." However, the experiments presented do not address this hypothesis. The study does not demonstrate that any of the identified membrane protein targeting signals (mPTSs) of the proteins examined (e.g., PEX2, PEX3, PEX13, PEX14, or PMP70) contribute to their degradation. The study should be properly contextualized, or the authors must provide direct experimental evidence to support their claim.

We agree with the reviewer that the original manuscript did not provide direct experimental evidence that mPTSs contribute to recognition by the PQC pathway. We have now addressed this experimentally in the revised manuscript.

Our data show that PMPs degraded upon peroxisome loss belong to diverse classes, including single- and multi-pass proteins with different topologies. Since all are targeted by the same E3 ligases (MARCH5 and MUL1), they likely share a common recognition feature. Given their varied cytosolic domains, the most plausible shared element is the TMD.

To test this idea, we used the single-pass, tail-anchored PMP PEX26 as a simplified model, as its TMD and short C-terminal sequences are sufficient for peroxisomal targeting (PMID: 16763195). In our mass spectrometry analysis, PEX26 was degraded upon peroxisome loss (Supplementary Fig. 2c and Supplementary Data 1), which we confirmed using endogenous antibodies (Figure 1d–g). Its degradation is dependent on MARCH5 and MUL1 (Fig. 3f,g)

To directly test whether the TMD mediates recognition, we generated a GPS reporter in which GFP is fused to the minimal TMD and C-terminal region of PEX26 (GFP-PEX26(TMD)) and assessed its stability by flow cytometry and subcellular localization by microscopy. GFP-PEX26(TMD) stability decreased in PEX3 KO cells and was restored upon MARCH5/MUL1 ablation (**Fig. 5a,b)**. The reporter also showed increased mitochondrial co-localization in MARCH5/MUL1/PEX3 TKO cells **(Fig. 5c,d),** consistent with mitochondrial localization driven by the TMD upon peroxisome loss, followed by MARCH5/MUL1-dependent degradation.

Together, these results demonstrate that the PEX26 TMD is sufficient to confer recognition by the MARCH5/MUL1 PQC pathway, directly supporting our hypothesis that membrane-embedded elements of PMPs mediate substrate recognition.
These new data have been incorporated as new **Fig. 5** into the revised manuscript.

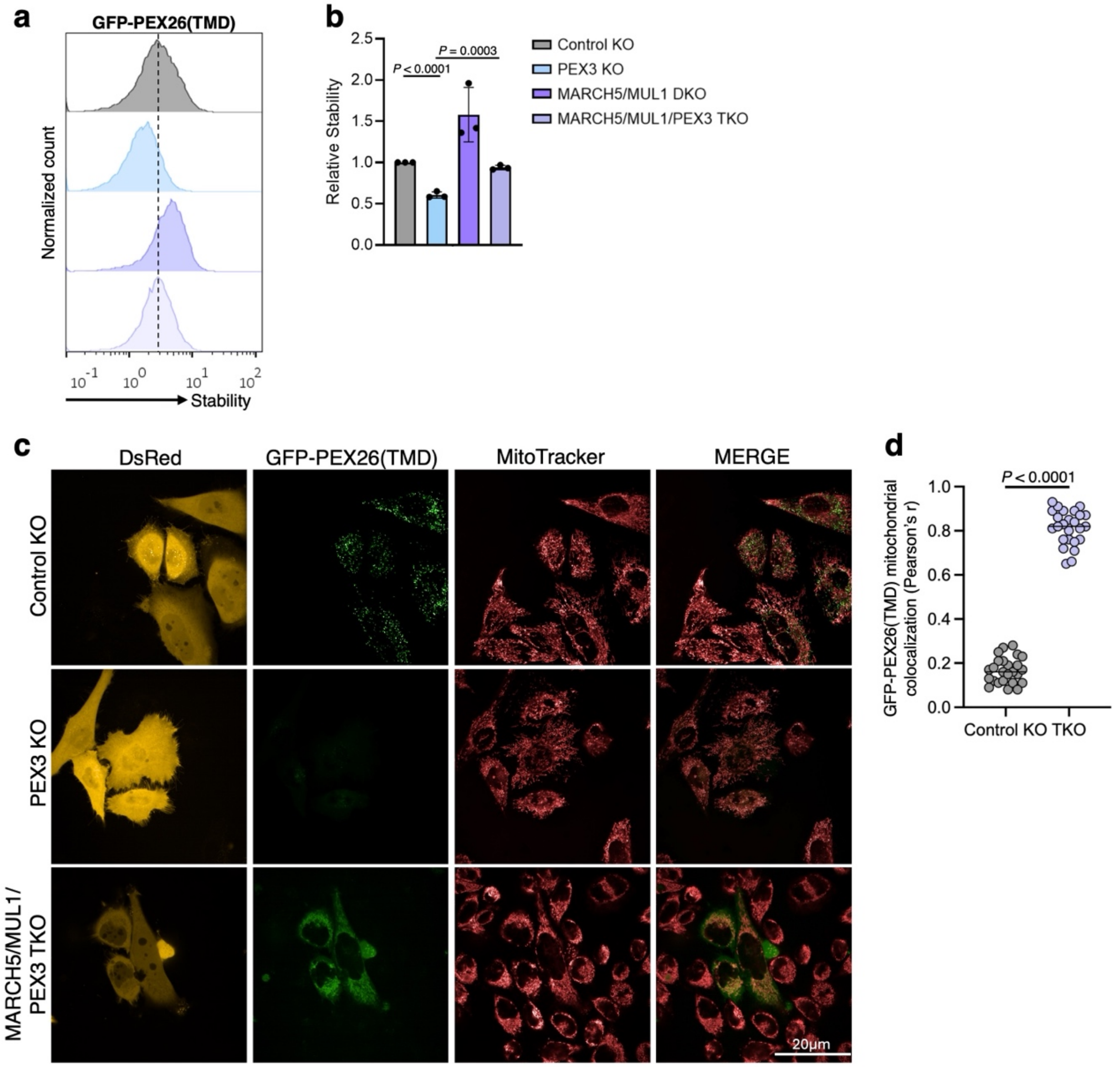

Fig. 5. Transmembrane domains mediate recognition of PMPs by MARCH5 and MUL1. (a,b) Stability analysis of PEX26(TMD) GPS reporter in HEK293T control cells, MUL1/MARCH5 DKO cells, PEX3 KO cells, and PEX3 KO cells additionally lacking both MUL1 and MARCH5 (TKO). Representative flow cytometry histograms are shown in (a) and quantitative analysis (mean GFP/DsRed fluorescence normalized to control KO ± s.d.) from n = 3 independent experiments are shown in (b). P values were determined using a two-tailed unpaired t-test. (c) Representative confocal fluorescence microscopy images of the GFP-PEX26(TMD) reporter expressed in the indicated genetic backgrounds of HeLa cells. Orange, DsRed expression control; green, GFP-PEX26(TMD) signal; red, mitochondrial marker MitoTracker. MERGE panels show the overlay of the GFP-PEX26(TMD) and MitoTracker signals. Scale bar, 20 µm. (d) Pearson's correlation coefficient (Pearson's r) analysis for the colocalization of GFP-PEX26(TMD) with the MitoTracker signal. Data are presented as individual data points with mean values; n ≥ 25 cells were analyzed per sample. The P value was determined by a two-tailed unpaired t-test.

2. Results – The statement that mitochondrial targeting of PMPs occurs independently of PEX19 in peroxisome-deficient cells is already established (e.g., DOI: 10.1083/jcb.148.5.931). The authors should cite these prior studies and frame their results as confirmation rather than a novel finding.

We thank the reviewer for pointing this out. We have cited the prior study (DOI: 10.1083/jcb.148.5.931) and now present our results as confirmation rather than a novel finding, as follows:

Page 13: "Given that PEX19 functions as a shuttling factor for PMPs to the peroxisome, these findings are in line with previous studies demonstrating that mitochondrial targeting of PMPs occurs independently of PEX19 in peroxisome-deficient cells 37, confirming that this pathway does not require PEX19".

3. Discussion – The authors state that their results suggest that accumulation of mitochondria-localized PMPs may contribute to mitochondrial dysfunction upon peroxisome loss. However, this concept has already been demonstrated in prior studies (doi: 10.15252/embj.201487943; doi: 10.15252/embr.202051991; DOI: 10.15252/embr.202153790) and is therefore not novel. The authors should clarify this context and emphasize that the novelty of their work lies in identifying a new underlying mechanism.

We thank the reviewer for this comment. We have clarified that the novelty of our work lies in identifying the underlying degradation mechanism, while PMP-induced mitochondrial dysfunction has been reported previously:

Page 17: "Indeed, mitochondrial mislocalization of a subset of PMPs due to mutations in peroxisome biogenesis factors has been shown to impair mitochondrial structure and function … Our study builds on these observations by identifying a UPS-dependent degradation mechanism mediated by mitochondrial E3 ligases, which actively removes mitochondria-targeted PMPs upon peroxisome loss. This pathway likely becomes particularly important when targeting fidelity is challenged, thereby maintaining proteostasis and helping to limit mitochondrial damage.."

Experimental design, controls, and reproducibility

4. Figure 1a – The authors analyzed two PEX3 knockout clones, PEX3 KO1 and PEX3 KO2. In the PCA plot, however, the clones are separated by a large distance along PC1, despite being expected to be functionally similar and therefore cluster together? Does this separation indicate major differences between the clones, potentially due to off-target effects?

Based on our experience with proteomics of CRISPR-edited cells, the differences between the two PEX3 knockout clones reflect clonal variability rather than off-target effects, as both clones were derived from the same population edited with a single sgRNA. Importantly, we applied stringent criteria in our analysis and considered only changes that were significant in both clones, as determined by Welch's t-test, a standard approach in proteomics.

While the authors compare both clones in Fig. 1 and Supplementary Fig. 1, it is unclear which clone was used for the other experiments? Did they confirm the findings in the second clone reintroduce PEX3 to rule out off-target effects? This should be clarified in the manuscript.

We apologize for the confusion. To ensure that the results in Fig. 1a–d were not due to clonal or off-target effects, we performed four independent validation sets: **(i)** For the mass spectrometry validation (Fig. 1d,e), we used the parental CRISPR-edited cell population (not a single clone), generated using the same sgRNA as in the single clones. **(ii)** We generated knockout cells using a

second, independent sgRNA targeting PEX3 and used these cells in the KO–rescue experiments (Supplementary Fig. 3b–c) thereby minimizing the likelihood of sgRNA-specific off-target effects. **(iii)** We performed rescue experiments by reintroducing PEX3 or PEX19 cDNA into the knockout cells. Restoration of the phenotype upon cDNA re-expression provides an independent genetic complementation approach to exclude off-target effects. **(iv)** Finally, we show that the phenotype observed in PEX3-deficient cells is reproduced in PEX19 KO cells, confirming that peroxisome loss is responsible for the reduced PMP levels and ruling out off-target effects. We have now clarified in the text and Methods which CRISPR-edited cells were used in each experiment.

In addition, the term "control KO" is ambiguous. Do the authors mean the corresponding parental cell line? If so, it should be clearly annotated throughout the manuscript.

An sgRNA targeting AAVS1 was used as a negative control, which is an established control for CRISPR-based experiments (PMID: 24336569). We have now clarified this in the text as follows:

Page 6: "Cells edited with an sgRNA targeting the AAVS1 safe-harbor locus were used as a control knockout (KO), an established negative control for CRISPR-based experiments[22]."

5. The authors state that all experiments were independently repeated at least 3 times with consistent outcomes. However, the supplementary RNA datasets include only 2 wild-type samples. It is also unclear whether the flow cytometry plots represent averaged data from 3 independent experiments or a single selected dataset. In addition, the authors should quantify the blot signals from all 3 biological replicates and provide appropriate statistical comparisons for the relevant conditions.

We have now included quantification of both the flow cytometry data and Western blots from three independent biological replicates, together with representative plots and blots. These data are now shown in the figures and described in the corresponding figure legends and text.

Regarding the RNA-seq dataset, the experiment includes three replicates for each PEX3 KO clone and two replicates for the WT control. The two WT samples show a high degree of correlation ($R^2 = 0.9452$), and therefore consider them sufficient for comparison; nonetheless, we have now explicitly noted this in the Methods section.

6. Mitochondrial purification – The authors use a commercial kit to isolate mitochondria. However, this method relies on differential centrifugation, it produces mitochondria-enriched fractions rather than highly purified mitochondria. This is evident from the presence of peroxisomal markers in the mitochondrial fraction from control cells (Fig. 4c and S4d), indicating contamination by other organelles.

We thank the reviewer for pointing this out (also noted by Reviewer 3). We agree that the fraction obtained represents a mitochondria-enriched rather than a highly purified mitochondrial fraction and may contain peroxisomal contaminants. To address this, we repeated the mitochondrial isolation using a second commercial immuno-purification kit based on an anti-TOM22 antibody (Miltenyi Biotec, #130-094-532). Unfortunately, this kit also produced mitochondria-enriched fractions with residual peroxisomal contamination (data not shown). Beyond the technical limitations of these kits, this contamination may also reflect extensive peroxisome–mitochondria

contact sites (PMID: 29720625), which make it inherently challenging to obtain highly pure mitochondria.

Importantly, we have independently validated the complete loss of peroxisomes in PEX3 KO and MARCH5/MUL1/PEX3 TKO cells using PeroxiSPY650 (PMID: 38773129). In both cell lines, the peroxisomal signal was reduced to near-background levels **(Supplementary Fig. 5).** Consistent with this, immunoblotting for catalase (CAT), a peroxisomal matrix marker, shows no detectable signal in the mitochondria-enriched fractions from PEX3 KO and PEX3/MUL1/MARCH5 TKO cells **(Fig. 4c).**

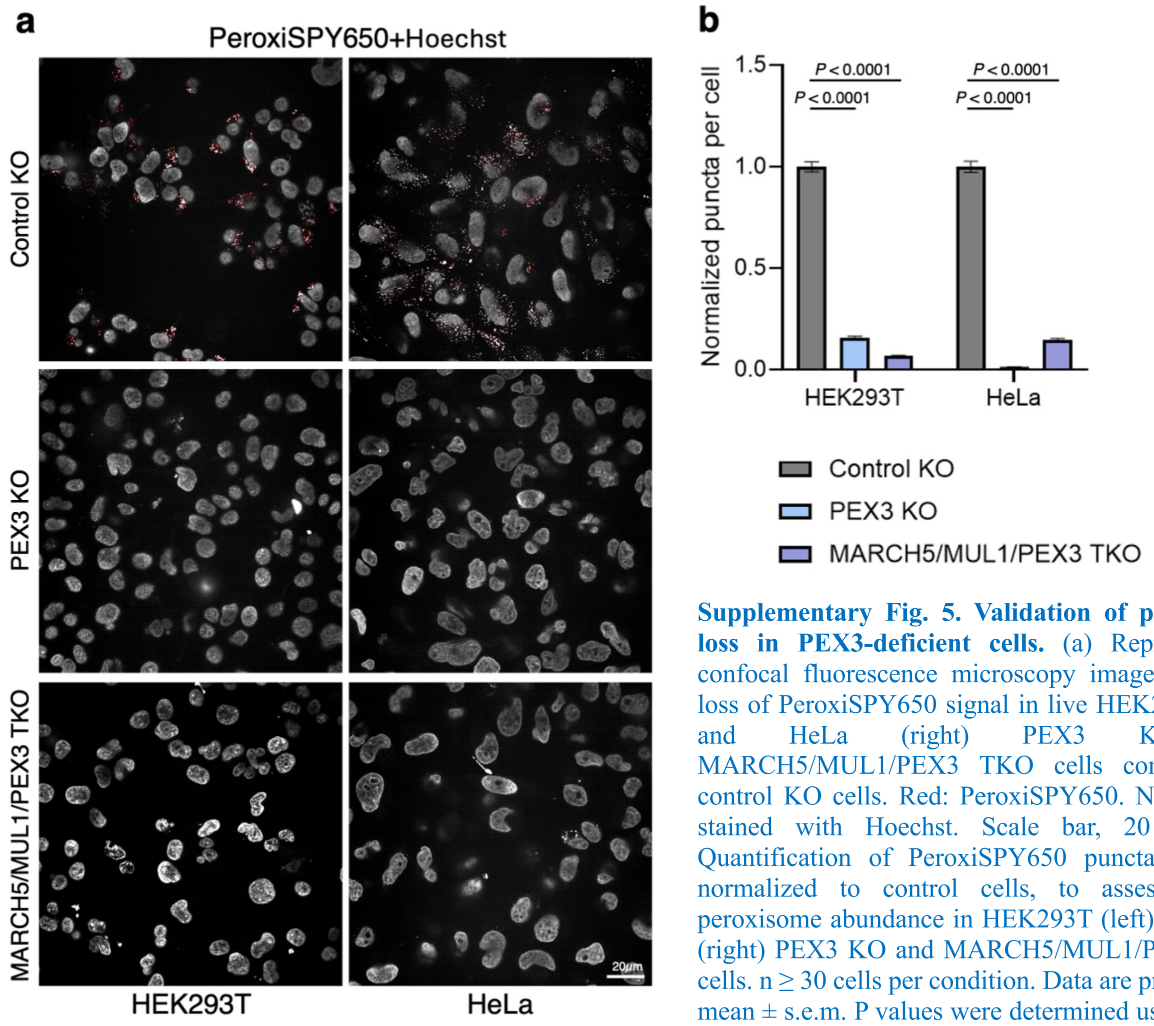

Supplementary Fig. 5. Validation of peroxisome loss in PEX3-deficient cells. (a) Representative confocal fluorescence microscopy images showing loss of PeroxiSPY650 signal in live HEK293T (left) and HeLa (right) PEX3 KO and MARCH5/MUL1/PEX3 TKO cells compared to control KO cells. Red: PeroxiSPY650. Nuclei were stained with Hoechst. Scale bar, 20 μm. (b) Quantification of PeroxiSPY650 puncta per cell, normalized to control cells, to assess relative peroxisome abundance in HEK293T (left) and HeLa (right) PEX3 KO and MARCH5/MUL1/PEX3 TKO cells. $n \geq 30$ cells per condition. Data are presented as mean ± s.e.m. P values were determined using a two-tailed unpaired t-test.

Thus, in the genetic background where peroxisomes are absent, the proteins detected in the mitochondria-enriched fraction originate from mitochondria rather than peroxisomal contamination. This allows us to directly compare PMP levels between PEX3 KO and PEX3/MUL1/MARCH5 TKO cells and to assess whether loss of MUL1 and MARCH5 specifically increases <u>mitochondrial</u>-associated PMPs.

Indeed, endogenous PMP levels were rescued specifically in the mitochondrial-enriched fraction of MARCH5/MUL1/PEX3 TKO cells, whereas PEX14 levels remained unchanged, as expected **(Fig. 4c,d)**.

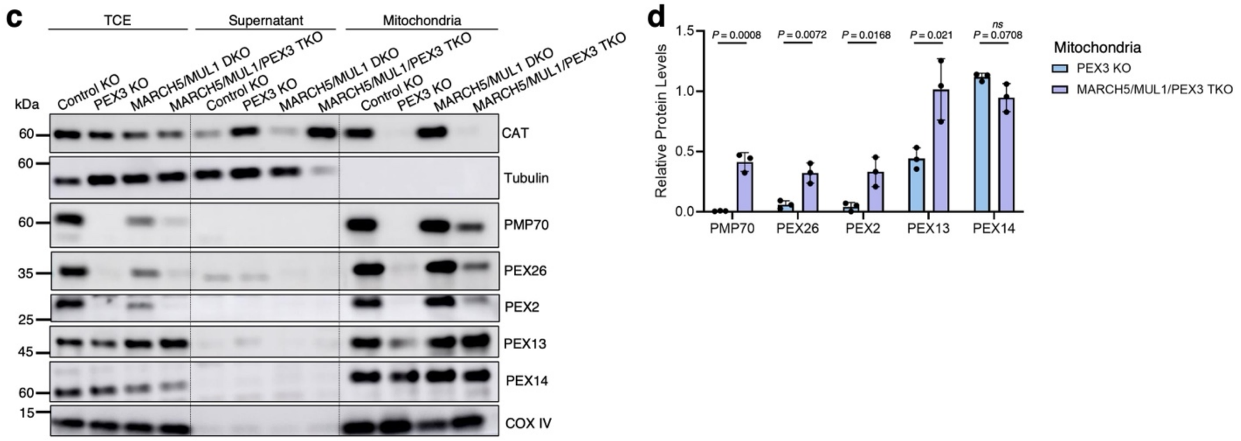

Fig. 4. PMPs are rerouted to mitochondria for proteasomal degradation under peroxisome loss. (c,d) Cellular fractionation of the indicated KO cells followed by immunoblot of endogenous PMPs in the mitochondria-enriched fraction ("Mitochondria") and the mitochondria-depleted supernatant ("Supernatant"). COX IV and Tubulin serve as mitochondrial or supernatant protein loading controls, respectively. CAT, a peroxisomal matrix protein, was included to confirm the absence of peroxisomal contamination in the mitochondrial fraction of PEX3-deficeint cells. A representative immunoblot is shown in (c), and quantitative analyses (mean ± s.d.) from n = 3 independent experiments are shown in (d). Quantification was normalized to control KO cells for consistency with analyses throughout the study. P values were determined using a two-tailed Student's t-test.

In addition, the term "cytoplasm" is inaccurate here, as it typically refers to the cellular content enclosed by the cell membrane but outside the nucleus, whereas the fraction labeled as such corresponds to the 12,000 x g supernatant. Finally, the term "mitochondrial lysis buffer" is misleading, as the purpose of this buffer is to lyse cells while preserving mitochondrial integrity rather than lysing mitochondria themselves.

We have corrected the terminology throughout the manuscript. The term "cytoplasm" has been replaced with "supernatant" in the text and figures, with the figure legends indicating that this fraction corresponds to a "mitochondria-depleted supernatant". "mitochondrial lysis buffer" has been changed to "mild lysis buffer preserving mitochondrial integrity," as suggested.

7. The authors identify MUL1 and MARCH 5 as E3 ligases ubiquitinating mislocalized PMPs in their CRISPR (Fig. 3b), yet later state that these ligases function redundantly in PMP degradation (Supplementary Fig. 3b). It is difficult to reconcile this conclusion with the screening conditions, as the sgRNA library DNA was delivered at an MOI of ~0.3. Under these conditions, and even with 1000 x library representation – the probability that any given cells receives sgRNAs targeting both MUL1 and MARCH5 is extremely low. If single gene knockouts produced minimal phenotypes, it therefore remains unclear how the screen could have detected this redundant pair. The authors should clarify how their screen design supports these findings or provide independent evidence validating the proposed redundancy.

CRISPR stability screens based on FACS sorting are highly sensitive to even small changes in protein stability. As shown in the screening pipeline (Fig. 3a), we isolate only the top 5% of GFP-positive cells. Although individual cells in the pooled screen typically carry only a single sgRNA (MOI ~0.3), even a modest stabilization of a GFP-tagged PMP can shift a subset of cells into this gate, leading to enrichment of the corresponding sgRNAs when analyzed by MAGeCK, particularly given the use of six independent sgRNAs per gene. Thus, partially redundant E3 ligases can be identified because each contributes detectably to substrate turnover, even if the strongest phenotype is observed only upon simultaneous loss of both proteins.

Consistent with this, Supplementary Fig. 3b shows that knockout of either MUL1 or MARCH5 in PEX3-deficient cells causes a small but reproducible stabilization of PMP34 and PEX11A, which is sufficient to be detected by the screen. In contrast, simultaneous loss of both ligases produces a much stronger stabilization, demonstrating their functional redundancy.

Importantly, this screening logic has been validated in our previous studies using the same platform to identify partially redundant E3 ligase substrate receptors, including FEM1A/B/C and ZYG11B/ZER1 (PMIDs: 31273098, 29779948).

8. The authors propose that MARCH5 and MUL1 continuously manage a small pool of mislocalized PMPs to maintain proteostasis and cell viability. It is unclear whether the effect is also observed in control cells (e.g., with or without Bortezomib or MLN7243 treatment) or in cells lacking both E3 ligases but with intact peroxisome biogenesis). The authors should clarify whether these conditions were tested and how they support the proposed model.

We thank the reviewer for these suggestions. This point was also raised by Reviewer 1 (comment #3), and we have now addressed it quantitatively.

Fluorescent microscopy analysis shows significantly increased co-localization of the PMP34 reporter with mitochondria in MARCH5/MUL1 DKO cells compared to control cells **(Supplementary Fig. 8a,b)**. As expected, this colocalization was less pronounced than that observed in cells deficient in peroxisomes (Supplementary Fig. 6a-c). These results suggest that upon exogenous expression, a fraction of PMPs is mistargeted to mitochondria even in cells with intact peroxisomes and is subsequently degraded by MARCH5/MUL1. This underscores the importance of this pathway in the surveillance of mislocalized PMP species. Our findings are consistent with previous reports showing that overexpression of PEX14 results in mitochondrial mistargeting (PMID: 38762172).

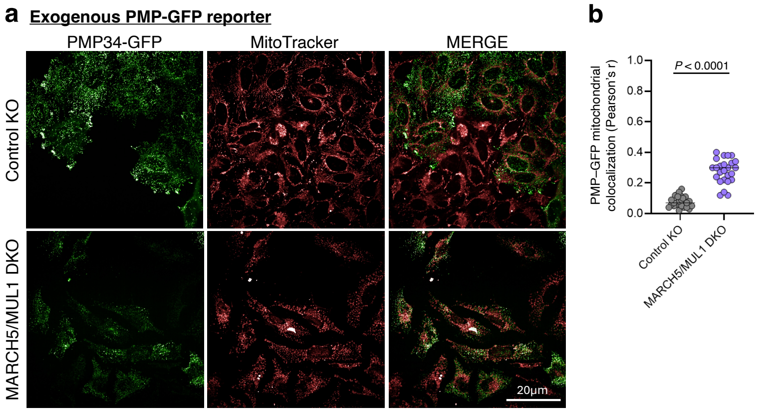

Supplementary Fig. 8. (a) Representative confocal fluorescence microscopy images of exogenous PMP34-GFP expressed in the indicated genetic backgrounds. Green, PMP-GFP signal; red, mitochondrial marker MitoTracker. Scale bar, 20 µm. (b) Quantification of colocalization between exogenous PMP34-GFP and MitoTracker using Pearson's correlation coefficient. $n \geq 25$ cells per condition. Data are presented as mean ± s.d. P values were determined by two-tailed unpaired t-test.

Consistent with these results, we performed quantitative analysis of the flow cytometry data. In cells expressing exogenous GPS-PMP reporters, PMP stability is reduced under conditions of peroxisome loss and is restored upon MARCH5/MUL1 double knockout (DKO). In cells lacking MARCH5 and MUL1 but retaining intact peroxisomes, we observe an increase in PMP stability; however, the degree of stabilization is lower than that observed in peroxisome-deficient cells **(Fig. 3c,d)**.

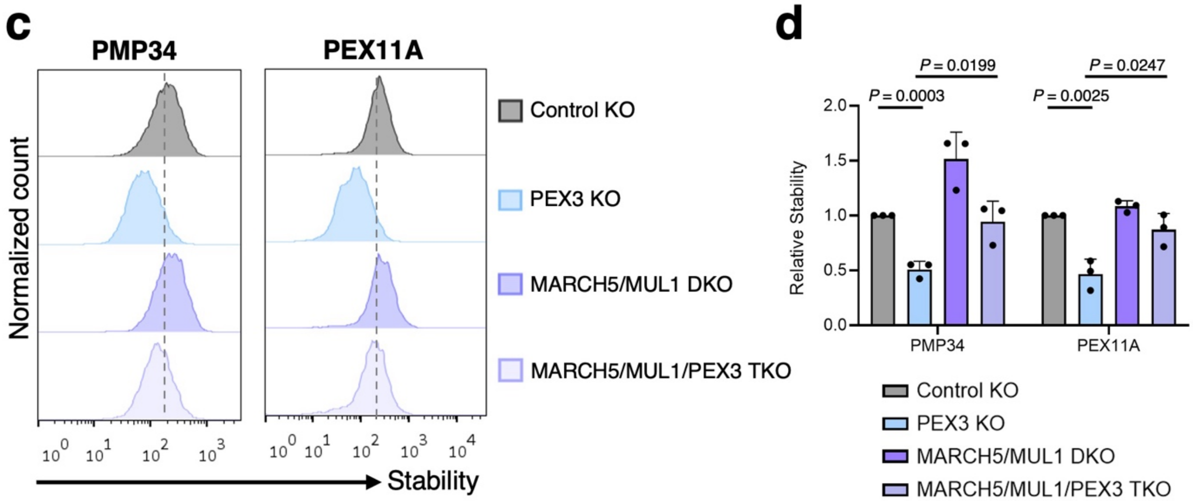

Fig. 3c,d. Stability analysis of PMP34 and PEX11A GPS reporters in control cells, MUL1/MARCH5 double KO (DKO) cells, PEX3 KO cells, and PEX3 KO cells additionally lacking both MUL1 and MARCH5 (TKO). Representative flow cytomeetry histograms (c) and quantitative analyses (mean GFP/DsRed fluorescence normalized to control KO ± s.d.) from n = 3 independent experiments (d) are shown. *P* values were determined using a two-tailed unpaired t-test.

Based on these observations, we hypothesize that, similar to other protein targeting pathways, peroxisomal protein targeting is not completely error-free. A small fraction of PMPs might mislocalize to mitochondria, a phenomenon that becomes more pronounced upon overexpression, as previously reported (PMID: 38762172). This mitochondrial pool of mislocalized PMPs is then recognized and degraded by MARCH5 and MUL1.

To determine whether this phenomenon also occurs under physiological conditions, we examined the localization of <u>endogenous</u> PMPs using a CRISPR knock-in cell line in which GFP was fused to PMP70 (PMID: 40689797). Importantly, in contrast to the exogenous expression setting, no significant increase in mitochondrial localization of these endogenous PMPs was observed in MARCH5/MUL1 DKO cells **(Supplementary Fig. 8c,d)**.

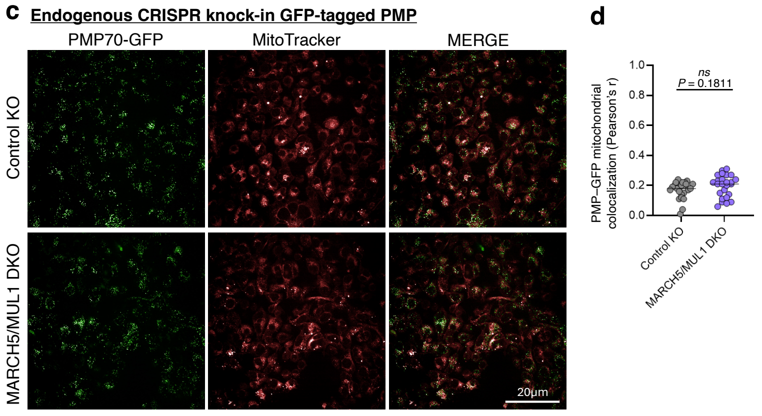

Supplementary Fig. 8. (c) Representative confocal fluorescence images of endogenous PMP70-GFP in the indicated genetic backgrounds of HEK293T CRISPR knock-in cells. Green, PMP70-GFP; red, MitoTracker. Scale bar, 20 µm. (d) Quantification of colocalization between endogenous PMP70-GFP and MitoTracker in control KO or MARCH5/MUL1 DKO cells using Pearson's correlation coefficient (Pearson's r). n ≥ 25 cells per condition. Data are presented as mean ± s.d. P values were determined by two-tailed unpaired t-test. ns, not significant.

In agreement with this, cellular fractionation experiments did not reveal increased steady-state levels of endogenous PMPs in the mitochondria-enriched fraction of MARCH5/MUL1 DKO cells compared to control KO cells **(Fig. 4c)** (see control KO [black bars] with MARCH5/MUL1 DKO [purple bars]).

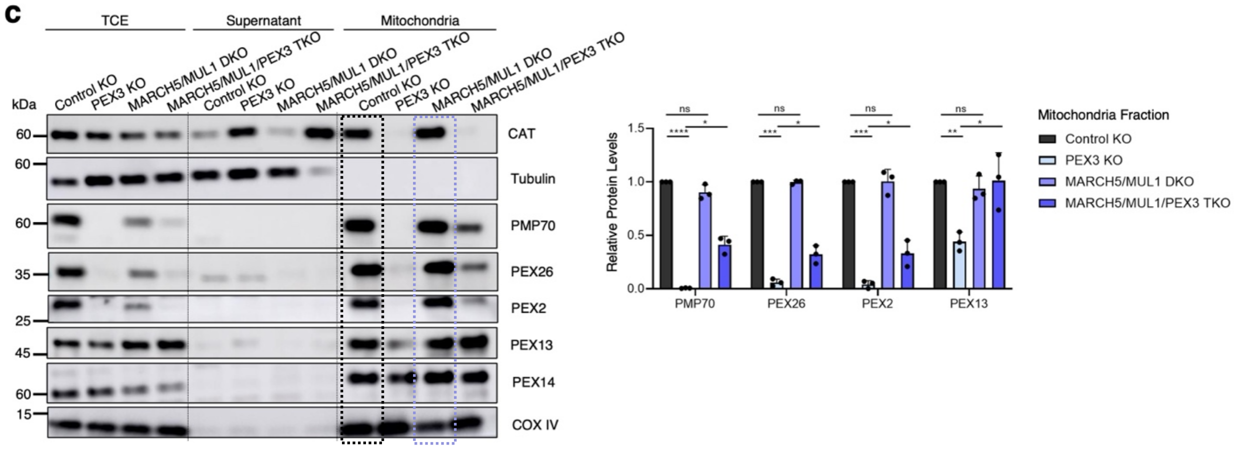

Fig. 4c. Cellular fractionation of indicated KO cells followed by immunoblot of endogenous PMPs in the mitochondria-enriched fraction ("Mitochondria") and the mitochondria-depleted supernatant ("Supernatant"). COX IV and Tubulin serve as mitochondrial or supernatant protein loading controls, respectively. A representative immunoblot is shown in (c). Quantitative analyses (mean ± s.d.) from n = 3 independent experiments is shown on the right. P values were determined using a two-tailed Student's t-test. *P < 0.01, **P < 0.001, ***P < 0.0001. ns- non-significant.

Together, these results indicate that while mistargeting of PMPs to mitochondria can occur upon overexpression and is subject to MARCH5/MUL1-dependent degradation, endogenous PMPs are efficiently and faithfully targeted to peroxisomes under physiological conditions. We cannot exclude the possibility that a very small fraction of endogenous PMPs is mistargeted; however, if present, such events are below the detection limit of our microscopy and biochemical approaches.

Data presentation

9. The Y-axes in the flow cytometry plots (Figs. 2c, 3c, S2b, S2D, and 3) need proper labeling. In addition, the authors should indicate how many cells were analyzed in each experiment.

We have now labeled the Y-axes in all flow cytometry plots as "Normalized Count". In addition, in accordance with the editorial guidelines, we have included the flow cytometry gating strategy in Supplementary Fig. 3a. We have also specified in the corresponding figure legends and in the Methods section that approximately 10,000 DsRed-positive cells were analyzed per sample in each flow cytometry experiment.

10. The authors report that 149 were downregulated and 63 upregulated in PEX3-deficient cells relative WT (Welch's t-test, $p < 0.05$; $\log_{10}$ fold change <-1 or >+1 for upregulated proteins) However, Supplementary Table 1 and Figures 1b,c present log2 fold-change values, not log10. This discrepancy should be corrected for consistency and accuracy.

We thank the reviewer for spotting this mistake. The fold-change values are indeed reported as $\log_2$, not $\log_{10}$, and this has now been corrected throughout the manuscript.

Nomenclature and accuracy

11. The authors state that catalase contains a peroxisomal targeting signal type 1 (PTS1), composed of the tripeptide serine-lysine-leucine (-SKL), is incorrect. Catalase carries a non-canonical PTS1 sequence (-KANL; DOI: 10.1083/jcb.134.4.849). Unless the authors have engineered this sequence – something not indicated in the Materials and Methods – the statement is inaccurate and should be corrected.

You are absolutely right. We have corrected the text to: *"As a functional readout, we expressed GFP fused to the peroxisomal matrix protein catalase (CAT), which contains a peroxisomal targeting signal type 1 (PTS1)."* We have avoided detailing canonical vs. non-canonical PTS1 sequences to prevent unnecessary confusion for the reader.

12. Protein descriptions in text and tables – Several proteins cannot be easily traced in the supplementary tables because the text uses synonyms while the tables use official nomenclature (e.g., ABCD3 is referred to as PMP70 in the text). The authors should standardize protein names across the manuscript and supplementary materials to ensure consistency and clarity for non-experts.

We thank the reviewer for pointing this out. We have standardized official protein names throughout the manuscript and supplementary materials. When gene and protein names differ, we indicate this in the text at the first mention. In the MS volcano plots (Fig. 1a and Supplementary Fig. 2c), protein names are also indicated in brackets where they differ from the gene names.

Reviewer #3 (Remarks to the Author):

The study by Segev-Nakar and Koren demonstrated the widespread downregulation of peroxisomal membrane proteins (PMPs) in cells lacking the PEX3 or PEX19 genes. These genes play a pivotal role in the targeting and import of PMPs to peroxisomes and are therefore essential for peroxisome biogenesis. The authors showed that the downregulation of PMPs in cells deficient in peroxisome biogenesis (PEX3 or PEX19 knockout (KO) cells) is due to decreased protein stability rather than reduced mRNA levels. They subsequently demonstrated that PMPs that fail to reach peroxisomes in PEX3 KO cells are instead directed to the mitochondria. There, the E3 ligases MUL1 and MARCH5 ubiquitinate the PMPs, promoting their degradation by the proteasome and safeguarding cellular proteostasis. Furthermore, the authors demonstrated that cells with triple KOs of PEX3/MUL1/MARCH5 exhibited greater impairment in cell proliferation than PEX3 KO or MUL1/MARCH5 KO cells. Unlike the degradation of mislocalised ER proteins by the cytosolic protein quality control (PQC), the authors proposed an additional layer of PQC involving mitochondria-residing E3 ligases in cells with deficient peroxisome biogenesis.

While the postulation of tight cross-talk between the peroxisome and mitochondria in protein quality control is interesting, unfortunately the data presented here do not fully support the authors' conclusion. In particular, their conclusion that PMPS are rerouted to mitochondria in PEX3 KO cells cannot be fully supported by their immunofluorescence and fractionation data. Secondly, it is unclear whether this is a general phenomenon as the PMPs degraded by MARCH5/MUL1 seemed limited. The specific points have been outlined below.

We thank the reviewer for noting that “the postulation of tight cross-talk between the peroxisome and mitochondria in protein quality control is interesting.” To strengthen our conclusions that PMPs are rerouted to the mitochondria and are degraded by MARCH5/MUL1, we have implemented the following additional validations:

1. We verified the complete loss of peroxisomes in PEX3 KO and PEX3/MARCH5/MUL1 triple KO (TKO) cells using the peroxisome-specific marker PeroxiSPY650 (PMID: 38773129).
2. Building on point 1, in the fractionation experiment, we focused our analysis on peroxisome-deficient cells (PEX3 KO and PEX3/MUL1/MARCH5 TKO cells), in which the absence of peroxisomes ensures that proteins detected in the mitochondria-enriched fraction originate from mitochondria rather than peroxisomal contamination. We have verified it using immunoblotting for catalase (CAT), a matrix peroxisomal protein. This protein is not rerouted to the mitochondria in PEX3 KO cells and provide a strong evidence that PEX3 KO and PEX3/MUL1/MARCH5 TKO cells are devoid of any contaminating peroxoisomes in the mitochondria fraction. This approach allowed us to directly assess whether loss of MUL1 and MARCH5 specifically increased mitochondrial-associated PMPs.
3. We quantified all immunofluorescence experiments in both HEK293T and HeLa cells to provide stronger evidence for co-localization between a mitochondria marker and PMPs in peroxisome-deficient cells.
4. We analyzed additional endogenous PMPs to assess their degradation by MARCH5 and MUL1 upon peroxisome loss. All proteins tested but one were regulated by

MARCH5/MUL1 indicating this phenomenon is not limited, but rather significant and plays a broad role in PMP quality control.

5. We provided evidence that the transmembrane domain (TMD) of PMPs is sufficient for MARCH5/MUL1 recognition of PMPs. Using a GFP reporter containing only the PEX26 TMD, we demonstrated it is sufficient to drive mitochondrial localization and trigger MARCH5/MUL1-dependent degradation.

Fig. 1: Add the positions of PMP70, PEX13 and PEX14 (Fig. 1e) to the diagram in Fig. 1b. In Fig. 1e, it would be more convincing if more PMPs were shown in immunoblotting in HEK293T cells, with quantification (mean, SE).

As suggested by the reviewer, we genereated a new Supplementary figure (Supplementary Fig. 2c) showing the positions of all PMPs. In addition, we tested multiple additional PMP antibodies (PEX10, PEX12, ABCD1, PEX16, and PEX26) for inclusion in Fig. 1e. Only validated antibodies that produced specific signals in immunoblotting (PEX16 and PEX26) were suitable for analysis and are now included in the revised figure with quantification. Consistent with the MS data (Fig. 1a), both PEX16 and PEX26 levels are reduced in peroxisome-deficient cells **(Fig. 1d-g)**.

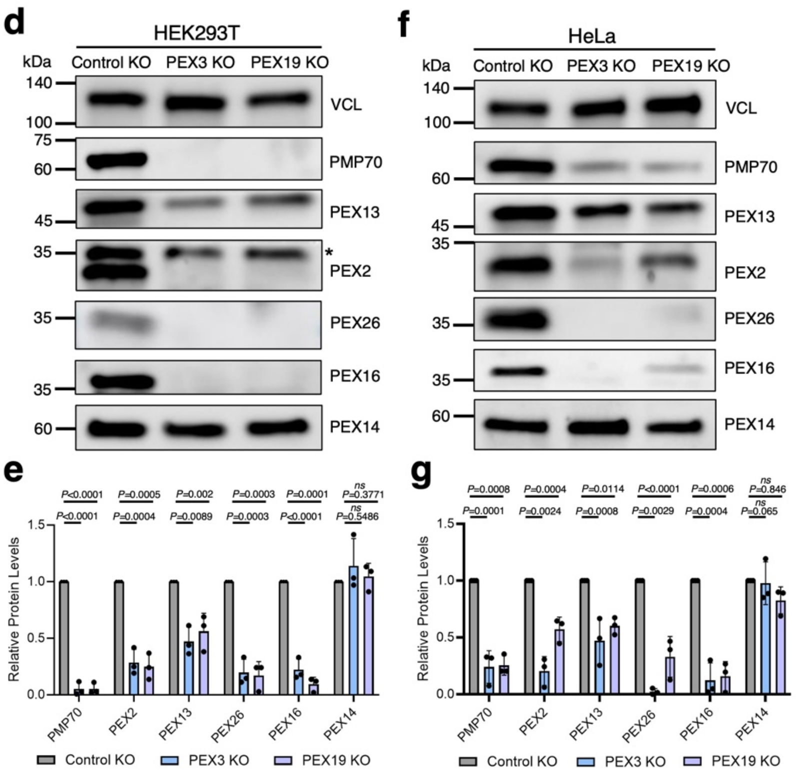

Fig. 1d-g. Immunoblot analysis of endogenous PMPs (PMP70, PEX13, PEX2, PEX26, PEX16, PEX14) in HEK293T (d-e) and HeLa (f-g) cells lacking PEX3 or PEX19. Vinculin (VCL) serves as a loading control. * denotes non-specific band observed in PEX2 blots. Representative immunoblots (d,f) and quantitative analyses (mean ± s.d.) (e,g) from n=3 experiments are shown. P values determined by two-tailed Student's t-test..

Among all these validated downregulated proteins upon peroxisome loss, 4 out of 5 analyzed proteins are regulated by MARCH5/MUL1 (**Fig. 3f,g** and Fig. 4c,d) indicating this phenomena is not limited, but rather significant and play a broad role in PMP quality control.

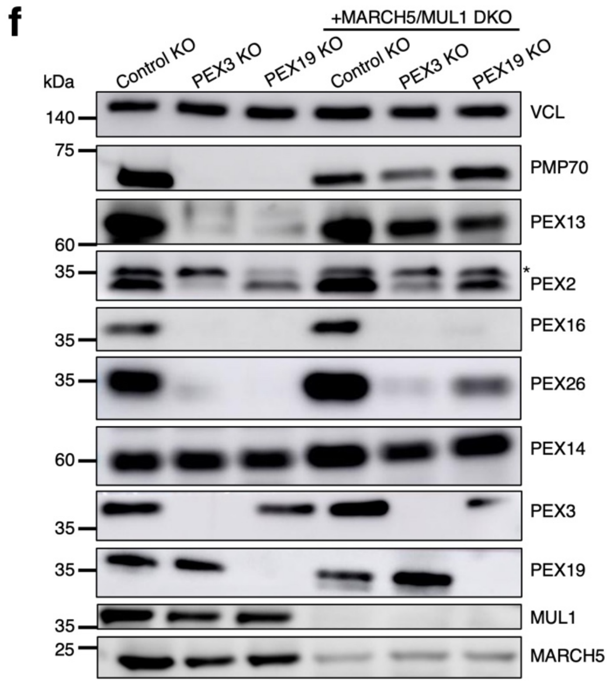

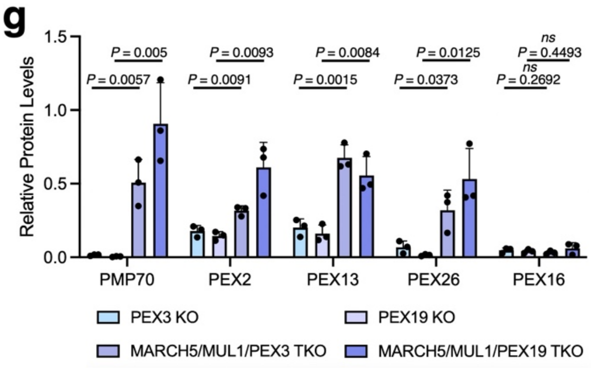

Fig. 3f,g. Immunoblot analysis of endogenous PMPs using specific antibodies in PEX3 or PEX19 KO cells with or without additional deletion of both MUL1 and MARCH5. VCL serves as a loading control. Representative immunoblots (f) and quantitative analyses (mean ± s.d.) from n=3 experiments are shown in (g). P values determined by two-tailed Student's t-test.

Fig. 2b: Include staining with a peroxisome-specific dye or marker, as well as co-staining with PEX11B-GFP in PEX3 and PEX19 KO cells.

We have now used the peroxisome-specific fluorescent reagent PeroxiSPY650 (PMID: 38773129) to co-stain with PMP34-GFP in PEX3 and PEX19 KO cells. These experiments show clear co-localization of PeroxiSPY650 with PMP34-GFP in control KO cells, while PeroxiSPY650 signal is absent in both PEX3 and PEX19 KO cells, consistent with the loss of peroxisomes in these backgrounds. **Fig. 2b** shows representative images of the strong co-localization between PMP34-GFP and PeroxiSPY650, and **Supplementary Fig. 1d,e** provide quantification of PeroxiSPY650 puncta per cell showing that the peroxisomal signal was reduced to near-background levels in these genetic backgrounds.
(We chose to use PMP34-GFP rather than PEX11B-GFP because PMP34 is a validated substrate of MARCH5 and MUL1 and is therefore directly relevant to the degradation pathway investigated in this study).

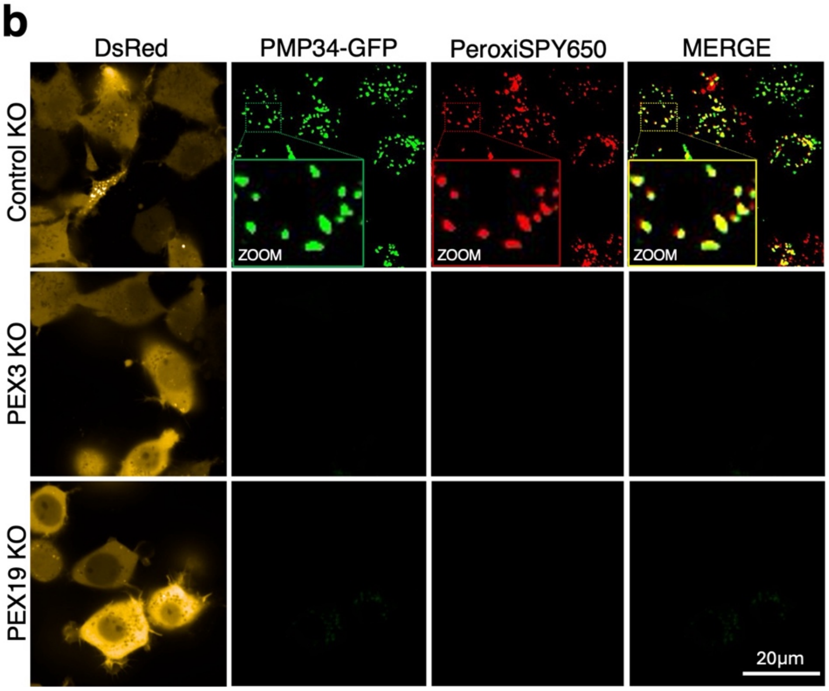

Fig. 2b. Representative fluorescence microscopy images of PMP34-GFP (a representative PMP reporter) and PeroxiSPY650 (peroxisome-specific reagent) in PEX3 or PEX19 KO cells compared to control cells. DsRed serves as an expression control and remains expressed in all cells. MERGE panels show the overlay of PMP34-GFP and PeroxiSPY650 signals. The zoomed regions demonstrate strong colocalization between the PMP34-GFP and PeroxiSPY650 signals. Scale bar, 20 µm.

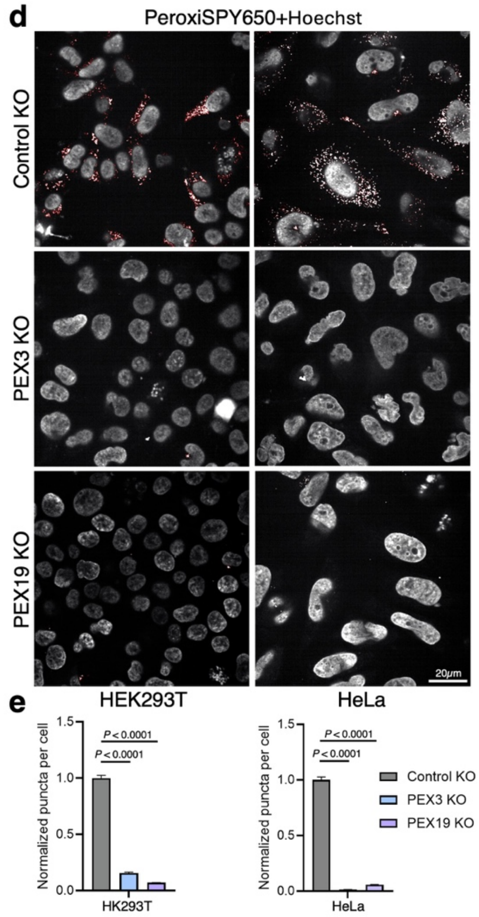

Supplementary Fig. 1d-e. Confocal fluorescence microscopy analysis showing loss of PeroxiSPY650 (peroxisome-specific reagent) signal in live HEK293T (left) and HeLa (right) PEX3 or PEX19 KO cells compared to control cells. Representative overlaid images are shown in (d). Red: PeroxiSPY650. Nuclei were stained with Hoechst. Scale bar, 20 µm. (e) Quantification of PeroxiSPY650 puncta per cell in PEX3 or PEX19 KO cells compared to control cells. n ≥ 1207 cells per genetic background for HEK293T and n ≥ 2215 cells for HeLa cells. Data are presented as mean ± s.e.m. P values were determined by Welch's t-test.

I strongly recommend observing PEX11B-GFP in the presence of bortezomib in PEX3 or PEX19 knockout (KO) cells. What is the expected subcellular localisation of PEX11B in KO cells treated with bortezomib?

Thank you for this suggestion. We have performed the experiment using two PMP-GPS reporters and observed cytoplasmic accumulation of the reporters in PEX3 KO cells treated with the proteasome inhibitor bortezomib (new **Supplementary Fig. 7a,c**). Quantitative analysis confirmed this cytoplasmic localization, showing a significant increase in colocalization (Pearson's r) between the PMP-GFP signal and DsRed, which serves as a nucleocytoplasmic marker and expression reference in the GPS system **(Supplementary Fig. 7b, d).** This observation is consistent with the known mechanism whereby organellar membrane proteins, including those of mitochondria, are ubiquitinated, extracted by AAA ATPases, and subsequently processed by cytosolic proteasomes (PMID: 21705204, 41498289). Notably, aggregates were observed in the cytoplasm of proteasome-inhibited, peroxisome-deficient cells, suggesting that interference with UPS-mediated clearance of PMPs may lead to their aggregation, presumably due to hydrophobic interactions driven by their TMDs. This underscores the importance of PQC of PMPs in preventing toxic accumulation upon mislocalization.

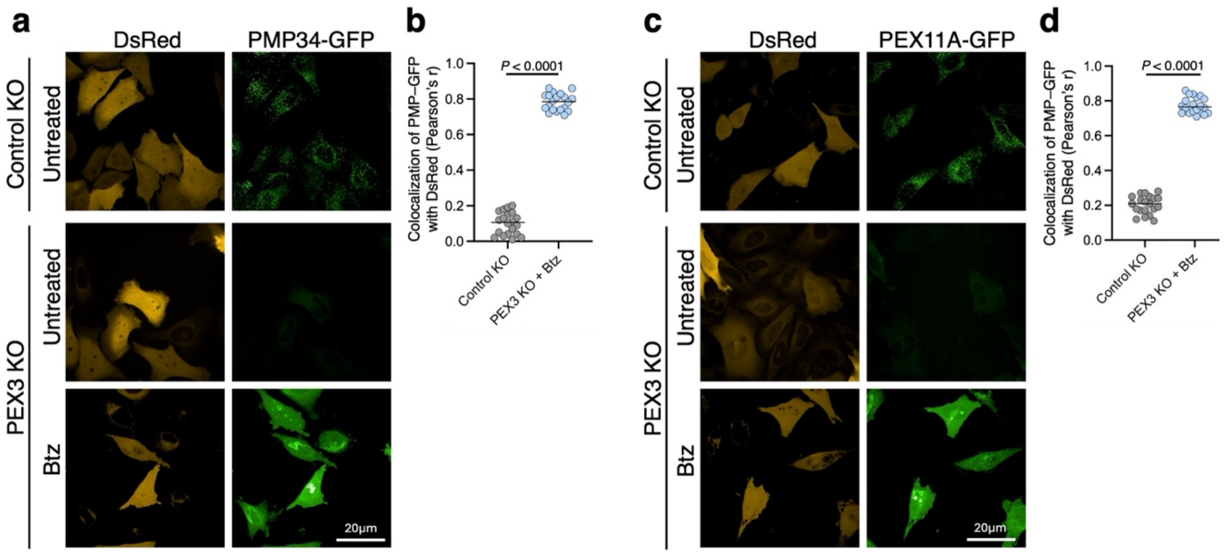

Supplementary Fig. 7. Proteasome inhibition leads to cytoplasmic accumulation of exogenous PMP-GFP reporters. (a-d) The indicated exogenous PMP–GFP reporters were expressed in control or PEX3 KO HeLa cells, either left untreated or treated with the proteasome inhibitor bortezomib (Btz; 1 µM, 16 h). (a,c) Representative confocal fluorescence microscopy images for PMP34-GFP (a) and PEX11A-GFP (c). Orange, DsRed expression control; green, PMP-GFP signal. Scale bar, 20 µm. (b,d) Pearson's correlation coefficient (Pearson's r) analysis of the colocalization between PMP-GFP and DsRed, serving as a nucleocytoplasmic marker, for the conditions shown in (a) and (c). $n \geq 20$ cells were analyzed per sample. The P value was determined by a two-tailed unpaired t-test.

Figure 3d: The authors would demonstrate an in vivo ubiquitination assay for endogenous PMP34 or other PMPs.

Unfortunately, in vivo ubiquitination of endogenous proteins remains a significant technical challenge in the UPS field. This is primarily due to the very low steady-state levels of endogenous substrates undergoing degradation, even under proteasome inhibition. Consequently, such assays traditionally rely on exogenous expression of tagged substrates together with tagged ubiquitin (e.g., HA–ubiquitin) to enable sensitive and reliable detection. In addition, the currently available antibodies against endogenous PMP proteins are not suitable for immunoprecipitation (data not shown), rendering direct assessment of endogenous ubiquitination technically unfeasible. To address the reviewer's suggestion, we have therefore included an additional example of an in vivo ubiquitination assay using exogenously expressed PEX11A, which is now presented in **Fig. 3e.**

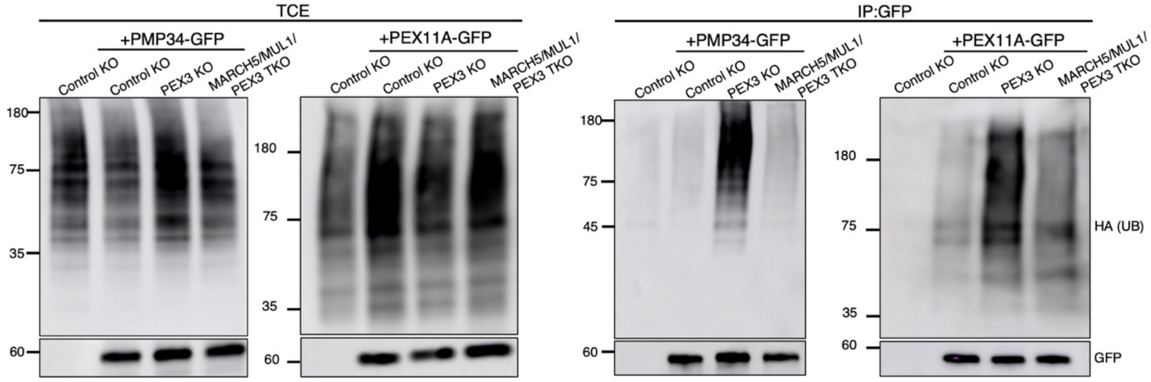

Fig. 3e. In vivo ubiquitination assay. HA-ubiquitin was transfected to control KO, PEX3 KO, and PEX3 KO cells additionally lacking both MUL1 and MARCH5 stably expressing PMP34-GFP or PEX11A-GFP, followed by IP with anti-GFP beads. Control KO cells not expressing PMP-GFP served as a negative control. Immunoblot analysis was performed on both total cell extracts (TCE) and GFP immunoprecipitates (IP:GFP) using anti-HA antibody to detect HA-ubiquitin ("UB") conjugates. Anti-GFP immunoblotting confirmed comparable levels of PMP-GFP in the immunoprecipitates across samples.

In Fig. 3e, changes in PEX2 levels were minimal. The authors could replace this or quantify the change.

All Western blot data throughout the manuscript have now been quantified. Specifically, PEX2 levels in Fig. 3e (now Fig. 3f) are quantified in the new **Fig. 3g**, demonstrating a significant increase in PEX2 levels in MARCH5/MUL1/PEX3 triple KO cells comapred to PEX3 KO cells.

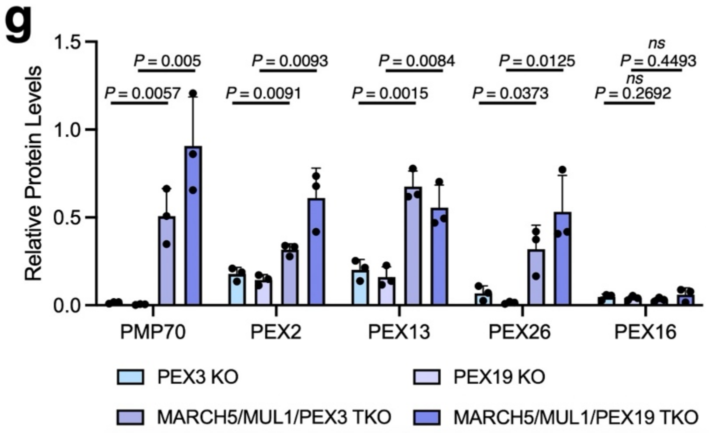

Fig. 3g. Quantitative analyses of the indicated proteins (mean ± s.d.) from n=3 experiments. Quantification was normalized to control KO cells for consistency with analyses throughout the study. P values determined by two-tailed Student's t-test.

Fig. 4a. Again, the authors should verify the absence of peroxisome (complete loss?) in PEX3 KO cells using other peroxisome-specific markers. Mitochondria are scattered throughout the cytoplasm in control KO cells, whereas perinuclear aggregation of mitochondria was observed in PEX3 and TKO cells. This perinuclear aggregation of mitochondria can occur dependent on organelle-specific motor proteins and microtubules. Is it possible that the remaining peroxisomes or PMPs in TKO cells move to the perinuclear region in these stressed conditions?

We have verified the complete loss of peroxisomes in PEX3 KO and TKO cells using the peroxisome-specific marker PeroxiSPY650 (PMID: 38773129) **(Supplementary Fig. 5).** These analyses confirm that in both cell lines, peroxisome abundance dropped to near-zero baseline level, thereby excluding the possibility that residual peroxisomes relocalize to the perinuclear region and contribute to the observed mitochondrial distribution.

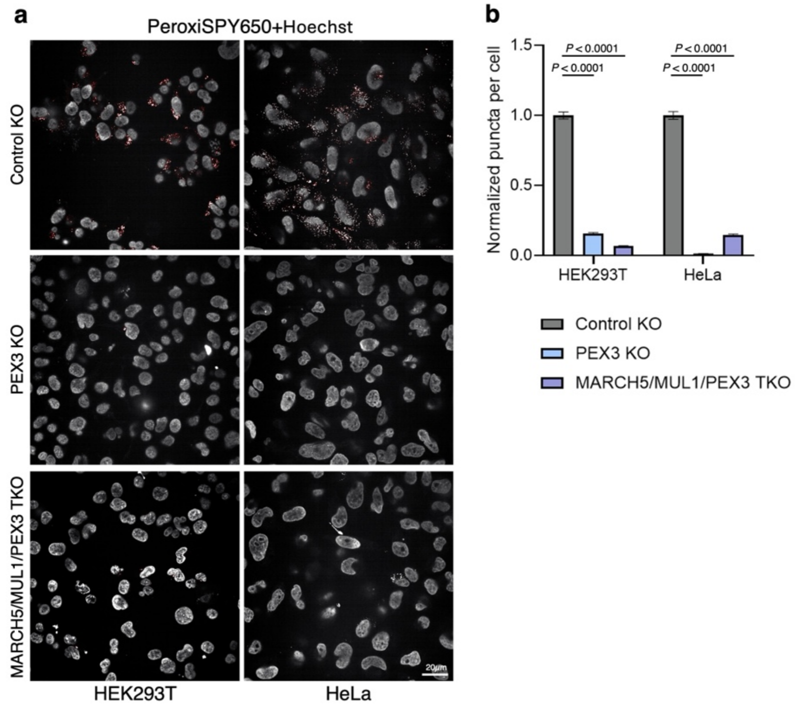

Supplementary Fig. 5. Validation of peroxisome loss in PEX3-deficient cells. (a) Representative confocal fluorescence microscopy images showing loss of PeroxiSPY650 signal in live HEK293T (left) and HeLa (right) PEX3 KO and MARCH5/MUL1/PEX3 TKO cells compared to control KO cells. Red: PeroxiSPY650. Nuclei were stained with Hoechst. Scale bar, 20 µm. (b) Quantification of PeroxiSPY650 puncta per cell, normalized to control cells, to assess relative peroxisome abundance in HEK293T (left) and HeLa (right) PEX3 KO and MARCH5/MUL1/PEX3 TKO cells. $n \geq 1907$ cells per genetic background for HEK293T and $n \geq 1750$ cells for HeLa cells. Data are presented as mean ± s.e.m. P values were determined by Welch's t-test.

To further address the reviewer's concern regarding potential alterations in mitochondrial organization, we quantified mitochondrial branch length using a semi-automated image analysis pipeline in ImageJ with the Mitochondria analyzer plugin (PMID: 31846372). We found no significant differences between control and KO cells in average branch length (**Fig. R1**), indicating that peroxisome loss does not measurably affect overall mitochondrial morphology.

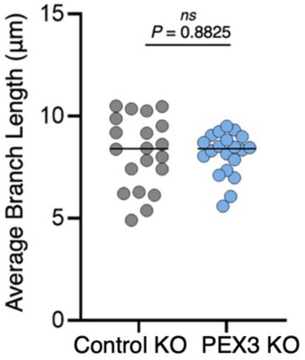

Fig. R1. Quantification of mitochondrial branch length in PEX3 or control KO cells. ns, not significant

Together, these findings argue against a role for residual peroxisomes or PMPs in driving mitochondrial distribution alterations.

Given the method of mitochondrial purification used, it is highly likely that the so-called 'mitochondria fraction' contains both mitochondria and peroxisomes. Indeed, the mitochondrial fraction of the control KO cells in Fig. 4c contained abundant PMP70, PEX13 and PEX14 along with TOM20. Therefore, the authors cannot conclude that PMPs are redirected to mitochondria without conducting further experiments to verify the complete loss of peroxisomes in TKO cells.

We thank the reviewer for pointing this out (also noted by Reviewer 2 (comment #6)). We agree that the fraction obtained represents a mitochondria-enriched rather than a highly purified mitochondrial fraction and may contain peroxisomal contaminants. To address this, we repeated the mitochondrial isolation using a second commercial immuno-purification kit based on an anti-TOM22 antibody (Miltenyi Biotec, #130-094-532). Unfortunately, this kit also produced mitochondria-enriched fractions with residual peroxisomal contamination (data not shown). Beyond the technical limitations of these kits, this contamination may also reflect extensive peroxisome–mitochondria contact sites (PMID: 29720625), which make it inherently challenging to obtain highly pure mitochondria.

Importantly, as noted above (see response to point 4a), we independently validated the complete loss of peroxisomes in PEX3 KO and MARCH5/MUL1/PEX3 TKO cells using PeroxiSPY650 (PMID: 38773129) **(Supplementary Fig. 5)**. Consistent with this, immunoblotting for catalase (CAT), a peroxisomal matrix marker, shows no detectable signal in the mitochondria-enriched fractions from PEX3 KO and PEX3/MUL1/MARCH5 TKO cells **(Fig. 4c below)**

Thus, in the genetic background where peroxisomes are absent, the proteins detected in the mitochondria-enriched fraction originate from mitochondria rather than peroxisomal contamination. This allows us to directly compare PMP levels between PEX3 KO and PEX3/MUL1/MARCH5 TKO cells and to assess whether loss of MUL1 and MARCH5 specifically increases mitochondrial-associated PMPs.

Indeed, endogenous PMP levels were rescued specifically in the mitochondrial-enriched fraction of MARCH5/MUL1/PEX3 TKO cells, whereas PEX14 levels remained unchanged, as expected **(Fig. 4c,d).**

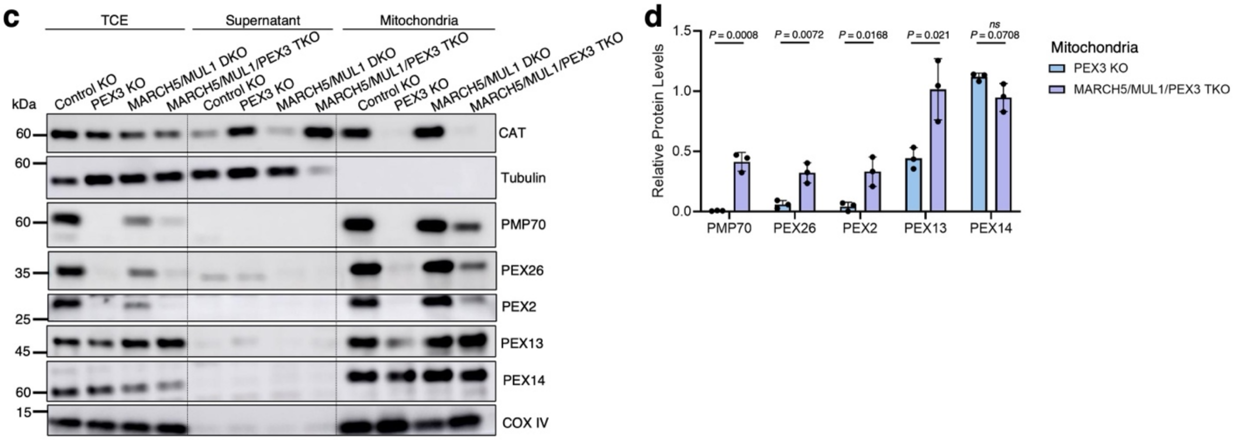

Fig. 4. PMPs are rerouted to mitochondria for proteasomal degradation under peroxisome loss. (c,d) Cellular fractionation of the indicated KO cells followed by immunoblot of endogenous PMPs in the mitochondria-enriched fraction (“Mitochondria”) and the mitochondria-depleted supernatant (“Supernatant”). COX IV and Tubulin serve as

mitochondrial or supernatant protein loading controls, respectively. CAT, a peroxisomal matrix protein, was included to confirm the absence of peroxisomal contamination in the mitochondrial fraction of PEX3-deficeint cells. A representative immunoblot is shown in (c), and quantitative analyses (mean ± s.d.) from n = 3 independent experiments are shown in (d). Quantification was normalized to control KO cells for consistency with analyses throughout the study. P values were determined using a two-tailed Student's t-test.

The authors should also include a co-IP experiment showing the interaction between MARCH5/MUL1 and PMP34 as well as some of other PMPs.

We thank the reviewer for this thoughtful suggestion. We agree that demonstrating a physical interaction by co-immunoprecipitation (co-IP) could further support the proposed relationship between MARCH5/MUL1 and PMPs. However, capturing E3 ligase–substrate interactions is widely recognized as technically challenging due to their transient nature, particularly for membrane-embedded proteins such as MARCH5/MUL1 and PMPs, where preservation of the native membrane environment is critical.

Our data, together with prior knowledge in the field, are consistent with a model in which mitochondrial E3 ligases directly mediate mitochondrial membrane protein degradation. Nevertheless, the conclusions of the current manuscript are based on functional genetics and biochemical evidence, and we have carefully phrased them accordingly. Given the time constraints of the revision process, we prioritized experiments that most directly strengthen the central conclusions of the study. We hope the reviewer agrees that the current data sufficiently support our claims.

Minor

Fig.1c. In PEX3 and PEX19 KO cells, GFP-CAT lost its punctate fluorescence pattern and displayed both diffused cytoplasmic and nuclear fluorescence.

This pattern is consistent with the literature. Catalase is a peroxisomal matrix protein containing a peroxisomal targeting signal type 1 (PTS1) and is not a membrane protein. It is not subjected to degradation upon peroxisome loss (Fig. 4c and Supplementary Data 1), and therefore displays diffuse cytoplasmic and nuclear fluorescence when peroxisomes are absent. We have clarified it in the text.

The left parentheses were missing in the indication of Supplementary figures throughout the text.

Thanks for catching this! We've corrected the missing parentheses.

We are grateful to the reviewers for their positive assessment of the revised manuscript and for acknowledging the improvements achieved through our revisions.

REVIEWER COMMENTS

Reviewer #1 (Remarks to the Author):

The authors addressed all of my concerns convincingly. The manuscript is suitable for publication in Nature Communications.

We thank the reviewer for their positive evaluation and for recognizing the suitability of our manuscript for publication in Nature Communications.

Reviewer #2 (Remarks to the Author):

The authors have adequately addressed all my concerns, and the manuscript has been significantly strengthened as a result. I greatly appreciate the authors' careful and thorough revision.

We thank the reviewer for their kind comments and for recognizing the substantial improvements made to the manuscript.

Reviewer #3 (Remarks to the Author):

I appreciate the authors' endeavor to address the comments by this reviewer. However, it is still missing the important biochemical evidence of interaction and ubiquitylation between PMPs and MARCH5/MUL1. With exogenously expressing GFP- or Myc-tagged MARCH5 with HA-ubiquitin (Yanagi S et al.,), the authors would be able to address these concerns.

We thank the reviewer for this constructive suggestion.

We agree that direct biochemical assays such as co-immunoprecipitation can provide valuable mechanistic insight into E3 ligase–substrate relationships. However, we would like to clarify that such interactions are inherently transient and technically challenging to capture in the context of integral membrane proteins such as PMPs and the E3 ligases MARCH5 and MUL1. Importantly, E3–substrate relationships are frequently established in the field using genetic dependency and functional ubiquitination assays rather than stable co-immunoprecipitation, given the transient nature of ubiquitination events.

With respect to substrate ubiquitination, the use of exogenously tagged substrates together with HA-ubiquitin is a well-established and the gold-standard approach in the ubiquitin–proteasome field. We performed this assay on two PMPs in the revised manuscript (Figure 3e), providing clear evidence of MARCH5/MUL1-dependent ubiquitination of PMPs. The reviewer's request to repeat this assay by monitoring endogenous substrate ubiquitination would not be feasible, given the extremely low abundance of endogenous PMPs, even in the presence of proteasome inhibition, which places them below the detection limit of available antibodies under conditions of peroxisome loss.

To address the reviewer's concern, we have revised the Discussion to explicitly acknowledge this limitation and clarify that while our data demonstrate MARCH5/MUL1-dependent degradation of PMPs, we do not directly demonstrate stable physical interactions between the E3 ligases and their substrates.

Page 17, end of first paragraph, we have added the following sentence (highlighted in red):

"Notably, while we demonstrate MARCH5/MUL1-dependent degradation of PMPs through genetic and functional assays, we do not directly assess physical interactions between the E3 ligases and PMPs due to the challenges associated with purifying membrane-embedded protein complexes, compounded by the transient nature of E3 ligase–substrate interactions".
